# Structure-based screening and a conformational biosensor identify a GPR183 inverse agonist and an activation switch

**DOI:** 10.1038/s41467-026-73857-9

**Published:** 2026-05-30

**Authors:** Louise Andersson, Michele Roggia, Kittikorn Wangriatisak, Rhiannon Skye Kozel, Holly R. Brittain, Sonia Youhanna, Maria Gil, Mathias Haag, Volker M. Lauschke, Karine Chemin, Sandro Cosconati, Paweł Kozielewicz

**Affiliations:** 1 Molecular Pharmacology of GPCRs, Department of Physiology and Pharmacology, Karolinska Institutet, Solna, Sweden; 2 DiSTABiF, University of Campania Luigi Vanvitelli, Caserta, Italy; 3 Division of Rheumatology, Department of Medicine, Solna, Karolinska Institutet, Karolinska University Hospital, Solna, Sweden; 4 Center for Molecular Medicine, Karolinska Institutet, Solna, Sweden; 5 Personalized Medicine and Drug Development, Department of Physiology and Pharmacology, Solna, Sweden; 6 Dr. Margarete Fischer-Bosch Institute of Clinical Pharmacology, Stuttgart, Germany; 7 University of Tübingen, Tübingen, Germany

**Keywords:** G protein-coupled receptors, Receptor pharmacology, Virtual drug screening

## Abstract

GPR183 is a chemotactic GPCR involved in immune cell migration. Using AI-driven virtual screening and biophysical assays, we identify inverse agonists. From 70 compounds and a subsequent hit expansion, compound 78 emerges as a potent inhibitor of constitutive and agonist-induced Gi signaling as well as β-arrestin2 recruitment. Binding within the receptor core is confirmed by a conformational biosensor, molecular dynamics simulations, and mutagenesis. The compound also blocks agonist-driven migration of peripheral blood mononuclear cells ex vivo with very high potency. Additionally, our analyses reveal key features of GPR183 activation, highlighting tyrosine 260 (Y260^6.51^) in transmembrane helix 6 as critical. Mutation of this residue alters compound 78 efficacy as well as induces receptor signaling bias, indicating a switch mechanism. Overall, this study provides tools to probe GPR183 function, identifies a chemical scaffold, and advances understanding of receptor activation, supporting therapeutic targeting in inflammatory, autoimmune, and cancer-related diseases.

## Abstract (web summary)

In this work researchers report a GPR183 inverse agonist with strong effects on immune cell migration and therapeutic potential. Using AI-driven screening and a developed conformational biosensor, the authors reveal how the receptor is activated and identify a switch controlling both inverse agonist efficacy and receptor signaling bias.

## Introduction

G protein-coupled receptors (GPCRs) constitute the largest family of membrane proteins in the human genome, represent key relays of cellular communication, and are the largest group of targets for marketed drugs^1^. Among them, GPR183 (also known as Epstein-Barr virus-induced gene 2, EBI2) is primarily a Gi-coupled receptor^2^, initially identified through its upregulation in B cells infected with Epstein-Barr virus^3^, involved in the guidance of B cells, T cells, and dendritic cells, within lymphoid and non-lymphoid tissues^4–6^. To this end, GPR183 is recognized as a chemotactic receptor for oxysterols^7,8^, with 7α,25-dihydroxycholesterol being the most active^2,8^. While the efficacy of this endogenous ligand has been validated for nanomolar concentrations in multiple studies from different laboratories^2,9–13^, at concentration levels corresponding to physiological levels of oxysterols^14,15^, the receptor is still classified as orphan by the International Union of Basic and Clinical Pharmacology (IUPHAR). In addition to ligand-induced signalling, constitutive activity of GPR183 in the apparent absence of ligand stimulation has been documented following receptor plasmid DNA overexpression as well as at endogenous levels of the receptor’s expression^16^. In this respect, the identification of inverse agonists is particularly relevant for the pharmacological and therapeutic modulation of this receptor.

Mounting evidence continues to implicate GPR183 in various physiological and pathological processes. To this end, high expression and/or activation of GPR183 plays a role in a range of diseases, including cancer^17^, metabolic disorders^18^, inflammatory^19^, and autoimmune diseases^20^. Importantly, GPR183 expression and activity appear to play context-dependent roles, beneficial in some settings, such as Burkitt lymphoma^17^, and detrimental in others, such as in acute myeloid leukaemia^21^. As such, pharmacological modulation of GPR183 is of growing interest, with both synthetic agonists^10,22^ and antagonists/inverse agonists^23–27^ being explored as potential therapeutic tools. Please note that, taking into consideration the dynamic nature of GPCRs and of GPCR–ligand interaction^28^, an observation that pure antagonists are in fact rare^29^ together with the already-mentioned constitutive activity of GPR183, we assume that a lot of the supposed GPR183 antagonists will exhibit negative efficacy (with efficacy being system dependent) and, as such, we will refer to these compounds simply as inverse agonists.

The pursuit of effective GPR183 modulators has led to the development of a few chemical classes of inverse agonists. Early efforts established the piperazine diamide scaffold, exemplified by NIBR189^23^, a selective and orally bioavailable inverse agonist, with an IC_50_ of 9 nM for an endogenous receptor in calcium release assay in U937 cells, that demonstrated preclinical efficacy in models of inflammatory bowel disease (IBD) and viral infections. Other compounds from this class include ML401^30^, which is potent in inhibiting GPR183-mediated chemotaxis of RS11846 cells expressing endogenous levels of GPR183 (IC₅₀ = 6.24 nM), and a benzo[d]thiazole derivative compound 33^25^, which achieved sub-nanomolar potency in cAMP assay using a GPR183-overexpressing HEK293 cells (IC₅₀ = 0.82 nM). In parallel, structurally distinct chemotypes have emerged, e.g., the spirocyclic inverse agonist GSK682753A (IC₅₀ = 56.6 nM in blocking overexpressed GPR183 constitutive activity in CREB-based luciferase reporter assay in HEK293 cells^31^) was instrumental in elucidating the receptor’s inactive state through Cryo-EM studies^2^. More recently, albeit still using NIBR189 as a starting compound, a novel class of difluoro-1,3-benzodioxole-piperazine-pyrimidine inverse agonists was disclosed, with a lead compound 32 (HY-162011) showing high potency in calcium mobilization assay in overexpressing CHO-K1 cells (IC₅₀ = 30 nM) and proving effective in a preclinical model of rheumatoid arthritis^24^. While these represent significant advances, the existing few scaffolds still present limitations that constrain a deeper exploration of GPR183 pharmacology. The discovery of new, structurally diverse GPR183 modulators is therefore essential not only to overcome chemotype-specific liabilities but also to unravel mechanistic details of receptor activation, inverse agonism, and signalling regulation of GPR183.

Here, to address the critical need for novel chemical scaffolds and to shed more light on the activation mechanism of this orphan GPCR, we exploited the predictive power of advanced computational chemistry. We hypothesized that an artificial intelligence (AI)-powered structure-based virtual screening (SBVS) could rapidly and effectively identify GPR183 modulators that traditional screening campaigns might overlook. In particular, in this study, we implemented the PyRMD2Dock approach developed by some of us, which leverages the classification power of the random matrix discriminant (RMD) algorithm to enhance the throughput of virtual screening campaigns for drug discovery^32^. This approach was coupled with the use of established bioluminescence resonance energy transfer (BRET)-based biophysical sensors (biosensors) and the development of a conformational biosensor to identify and characterize GPR183 ligands. Together, this led to the discovery of different chemotypes, of which the hit compound, inverse agonist compound 78, was also evaluated in cell-based migration assays to probe the inhibition of agonist-stimulated GPR183-mediated signalling at endogenous receptor expression. Importantly, our work reveals a specific pharmacological profile of 78 and a key role of Y260^6.51^ located in the orthosteric binding site in governing 78’s efficacy, receptor activation, and signalling bias. These findings establish tools for probing GPR183 function and provide insight for further therapeutic development targeting this receptor.

## Results

### AI-enforced virtual screen

**Fig. 1:**
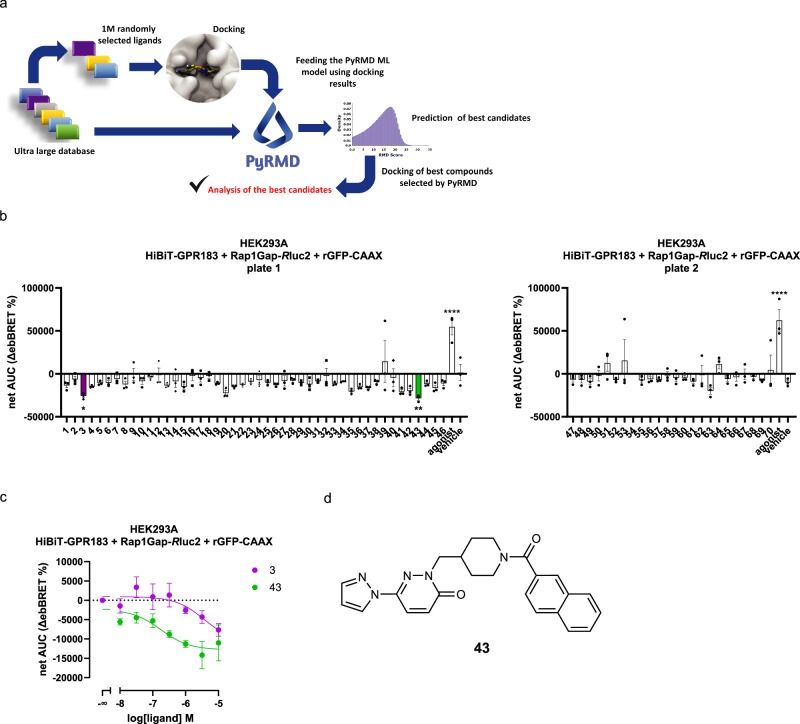
Small-molecule compounds inhibit the constitutive activity of GPR183. **a** The PyRMD2Dock workflow (created in MS Office PowerPoint). **b** The results from the 10 μM compounds screen using the ebBRET-based Gi activation assay with an overexpressed HiBiT-GPR183. The compounds were added to the cells, and the ebBRET was measured over 15 min. The data are presented as net AUC of ΔebBRET % over the three baseline reads measured prior to the compound addition; vehicle (0.1% DMSO) was not subtracted. The data are shown as mean ± SEM of three biological replicates. The data were analysed for differences with one-way ANOVA with Dunnett’s post-hoc analysis; *P  <  0.05; **P  <  0.01; ****P  <  0.0001. The selected compounds, 3 (*P* = 0.022) and 43 (*P* = 0.007), are highlighted. **c** The concentration response curve of compounds 3 and 43 in the same assay. The compounds were added, and the ebBRET signal was measured over 15 min. The data are presented as net AUC of ΔebBRET % over the three baseline reads measured prior to the compound addition; vehicle (0.1% DMSO) was subtracted. The data are shown as mean ± SEM of three biological replicates. **d** Chemical structural formula of 43 (ChemSketch 2021 2.0). Source data are provided as a Source Data file.

To discover other GPR183 binders, the PyRMD2Dock protocol was employed as depicted in Fig. 1a. It combines the ligand-based VS (LBVS) tool PyRMD^33^ with one of the most widely used docking software, AutoDock-GPU (AD4-GPU)^34^. By implementing PyRMD2Dock, it is possible to rapidly screen large chemical databases and identify those with the highest predicted binding affinity to a target protein. The first step of this workflow was to randomly select 1 million compounds from the screened database (in our case, we selected a set of ~10 million compounds from a subset of lead-like purchasable ZINC20 database^35^ accessed in February 2021), and subjected them to docking calculations employing the solved Cryo-EM structure of GPR183 in complex with its endogenous ligand 7α,25-dihydroxycholesterol (PDB 7TUZ^2^). For all the compounds, the predicted lowest binding free energy (Δ*G*_AD4_) was extracted, and the results were analysed based on their Δ*G*_AD4_ value distribution. In this study, compounds were categorized as “active” or “inactive” based on their docking scores (Δ*G*_AD4_). To establish these classes, we defined five distinct “activity” thresholds (≤−9.5, −10.0, −10.5, −10.75, and −11.0 kcal mol^−1^) and five “inactivity” thresholds (≥−4.0, −4.5, −5.0, −5.5, and −6.0 kcal mol^−1^). This created 25 unique datasets by combining each active threshold with each inactive threshold. The performance of our classification model is highly dependent on the ε cutoff values, which define the maximum allowed projection distance for active and inactive compounds within the training’s linear subspace. To optimize these parameters, we performed a comprehensive benchmarking analysis. For each of the 25 datasets, we employed a repeated stratified *κ*-fold cross-validation (5 folds, 3 repetitions) and systematically tested ε cutoff values from 0.01 to 0.99 (in steps of 0.10) for both classes. The optimal model was identified by the best trade-off between the true positive rate (TPR) and the false positive rate (FPR). This was achieved using the dataset defined by an active threshold of ≤−10.0 kcal mol^−1^ and an inactive threshold of ≥−6.0 kcal mol^−1^. The best-performing model, which yielded a TPR/FPR trade-off of 0.69, utilized an ε cutoff value of 0.99 for the active set and 0.80 for the inactive set. The remaining ~9 million compounds from the selected chemical databases were screened using the PyRMD screening module trained with the docking results, employing default settings and the *ε* cutoff values reported above. PyRMD returned a list of compounds classified as possible GPR183 binders with an associated confidence score (RMD score) of its predictions. Subsequently, the best 50,000 GPR183 binders predicted by PyRMD (i.e., the ones with the highest RMD score) were then docked into the GPR183 receptor, and to further refine the selection, we subsequently applied a pose filtering scheme aimed at selecting the ligands that were predicted to directly contact Y116^3.37^, Y260^6.51^, R87^2.60^, and Y112^3.33^ (numbers in the superscript indicate residue positions according to the Ballesteros–Weinstein numbering scheme^2,36^). These selection criteria, and a subsequent visual inspection, led to the selection of 70 GPR183 candidate binders.

### GPR183-mediated Gi activation—compounds from the AI-enforced VS

Based on the VS results, 70 compounds were purchased and analysed in an ebBRET-based assay to measure their effects on the activation of Gi (assessed as dissociation of the heterotrimeric G protein) mediated by the constitutive activity of overexpressed GPR183, at the cell membrane of HEK293A cells. In the first set of experiments, we have evaluated all 70 compounds at a fixed concentration of 10 μM (Fig. 1b). Using the cut-off defined by the statistically significant difference (one-way ANOVA test with Dunnett’s post-hoc analysis, *P* < 0.05) in the ebBRET between the vehicle (0.1% DMSO)—and compound-treated cells over 15 min, we selected two compounds that significantly reduced ebBRET, indicative of the decrease in the constitutive activity of heterologous GPR183: 3 (4-{2-[4-(2-fluorophenyl)−4-hydroxy-octahydro-1H-isoindol-2-yl]−2-oxoethyl}−1,2-dihydrophthalazin-1-one) and 43 (2-{[1-(naphthalene-2-carbonyl)piperidin-4-yl]methyl}−6-(1H-pyrazol-1-yl)−2,3-dihydropyridazin-3-one). Had a less stringent selection cut-off been defined using the LSD-Fisher post-hoc test, an additional 12 compounds would have met the criteria for significant reduction of the ebBRET signal (compounds: 4, 15, 20, 21, 35, 36, 37, 41, 42, 45, 51, 53). This cohort represents 20% of the total compounds evaluated in these assays, which is in line with the success rate expected in structure-based VS campaigns^37^. The representative kinetic plots can be found in Supplementary Fig. 1a, left.

Compounds 3 and 43 were then studied in the next step (Fig. 1c), where the aforementioned ebBRET-based assay was reiterated by a 15 min application of a range of different concentrations of the two molecules, spanning from 10 nM to 10 μM. In these assays, compound 3 inhibited the GPR183-mediated Gi activation with pIC_50_ = 5.44 (95% CI: not converged—6.48), and compound 43 inhibited the GPR183-mediated Gi activation with a higher potency and pIC_50_ = 6.76 (95% CI: 5.93–7.87). To this end, these data supported the selection of compound 43 for further investigations (Fig. 1d).

### Hit expansion, GPR183-mediated Gi activation and β-arrestin2 recruitment

**Fig. 2:**
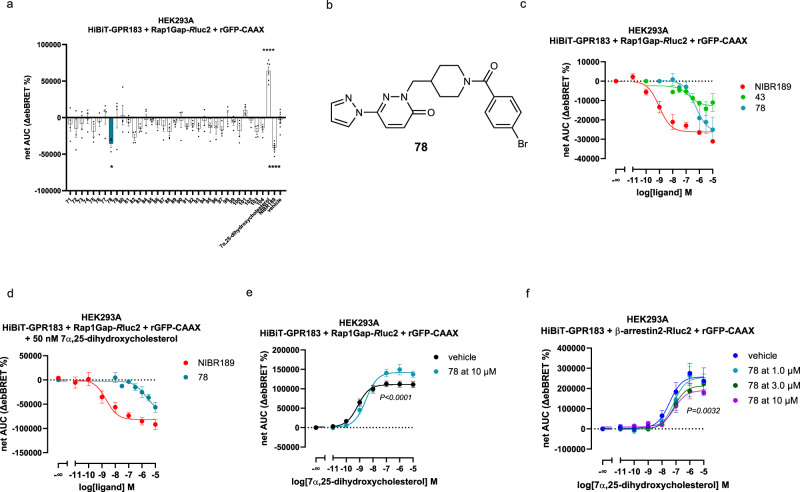
Analogues of compound 43 inhibit constitutive and agonist-stimulated GPR183 activity. **a** The results from the screen with the 34 analogues of 43. The compounds were used at 10 μM in the ebBRET-based Gi activation assay (15 min). The data are presented as net AUC of ΔebBRET % over the three baseline reads measured prior to the compound addition; vehicle (0.1% DMSO) was not subtracted. The data are shown as mean ± SEM of three (compounds 78, 86, 91, 96), four (the other analogues), six (7α,25-dihydroxycholesterol, NIBR189), or seven (vehicle) biological replicates. The data were analysed for differences with one-way ANOVA with Dunnett’s post-hoc analysis; *P  <  0.05; **P  <  0.01. The selected compound 78 is highlighted (*P* = 0.024). **b** Chemical structural formula of 78 (ChemSketch 2021 2.0). **c** The concentration response curve of compounds 43, 78 and NIBR189 in the same assay paradigm. The data are shown as mean ± SEM of three (43, 78) or four (NIBR189) biological replicates; vehicle (0.1% DMSO) was subtracted. The same data for the 43 are also shown in Fig. 1c. **d** The concentration response curve of compounds 78 and NIBR189 following 15 min preincubation and subsequent 15 min stimulation with 50 nM 7α,25-dihydroxycholesterol. The data are shown as mean ± SEM of four biological replicates; vehicle (0.1% DMSO) was subtracted. **e** Preincubation with 10 μM of 78 (6 min) modified agonist-simulated (15 min) GPR183-mediated Gi activation. The data are shown as mean ± SEM of three biological replicates; vehicle (1.0% DMSO) was subtracted. Differences in logEC₅₀ values and in the top or bottom plateaus of the curves were analysed using an *F*-test (one-sided; *P* < 0.0001). **f** Agonist-stimulated HiBiT-GPR183-mediated recruitment of β-arrestin2-Rluc2. The concentration response curve of 7α,25-dihydroxycholesterol ±78. 78 or vehicle was added, and the ebBRET signal was measured over 10 min, followed by the addition of different agonist concentrations. The plate was measured for another 15 min. The data are shown as mean ± SEM of three biological replicates; vehicle (1.0% DMSO) was subtracted. Differences in logEC₅₀ values and in the top or bottom plateaus of the curves were analysed using an *F*-test (one-sided; *P* = 0.0032). Source data are provided as a Source Data file.

To further explore the chemical space around this promising hit, a similarity search was performed to identify derivatives of compound 43. This involved selecting compounds featuring structural similarity with the hit (Tanimoto index > 0.70). Through this process, we identified 34 derivatives (Supplementary Table 1) that were purchased and assessed in the same 15 min ebBRET-based Gi activation experimental setup at a fixed concentration of 10 μM (Fig. 2a and Supplementary Table 1).

Again, using the cut-off defined by the statistically significant difference (one-way ANOVA test with Dunnett’s post-hoc analysis, *P* < 0.05) in the ebBRET between the vehicle (DMSO)-treated cells and compound-treated cells, we have selected compound 78 (2-{[1-(4-bromobenzoyl)piperidin-4-yl]methyl}5.−6-(1H-pyrazol-1-yl)−2,3-dihydropyridazin-3-one) (Fig. 2b). If a less stringent LSD-Fisher post-hoc test had been used to define a selection cut-off, one more compound would have significantly reduced the ebBRET signal (compound 82), equalling 5.9% of the total number of compounds tested in these assays.

Based on the statistical analysis of the in vitro testing of the similarity screen compounds, we proceeded with compound 78. As such, we performed the aforementioned ebBRET-based assay applying a range of different concentrations of the molecule, spanning from 10 nM to 10 μM (raw data can be found in Supplementary Fig. 1a, right). In these assays, compound 78 inhibited the GPR183-mediated Gi activation (15 min measurement) with a pIC_50_ = 6.29 (95% CI: 5.69–6.86). Although the parent compound 43 showed a trend towards higher potency, the difference was not statistically significant (*F*-test *P* = 0.43). However, compound 78 demonstrated significantly higher efficacy (ebBRET_min_) (*P* = 0.01), rendering it a more interesting candidate for further analysis (Fig. 2c). Importantly, compound 78, used at the highest applied concentration of 10 μM, showed very weak to no efficacy and/or presented itself with kinetic traces indicative of assay artefacts, on the cells transfected with ssDNA, CB_1_R (a Gi-coupled receptor), SMO (another Gi-coupled receptor and, similarly to GPR183, it also binds to sterols, which renders SMO the most relevant control) or β_2_AR (a bona fide Gs-coupled receptor) in the same experimental paradigm, pointing towards its good GPR183 selectivity over the endogenous Gi and Gs-coupled receptors^38^ (the assay is sensitive enough to detect signalling from endogenous receptors^39^) and the three selected overexpressed GPCRs in HEK293A cells (Supplementary Fig. 1b). Data for a high-potency GPR183 ligand NIBR189 are shown for comparison (pIC_50_ = 9.01 (95% CI: 8.12–9.85)). Next, we also used 78 in a competition assay where compound 78 was incubated together with the agonist 7α,25-dihydroxycholesterol (50 nM). Following a set of kinetic experiments, with a 15 min incubation of compound 78 followed by a 15 min stimulation with 7α,25-dihydroxycholesterol, we established that compound 78 inhibited the agonist-induced and GPR183-mediated activation of Gi with the pIC_50_ = 5.73 (95% CI: 4.73–6.48) (Fig. 2d). Data for NIBR189 (pIC_50_ = 8.68 (95% CI: 7.84–9.33)) are used for comparison. When a full concentration range of 7α,25-dihydroxycholesterol (10 pM–10 μM) was applied following a 6 min incubation with a fixed 10 μM concentration of compound 78, the expected rightward shift was moderate but statistically significant (pEC_50_ = 9.14 (95% CI: 8.90–9.37) vs. pEC_50_ = 8.53 (95% CI: 8.26–8.79)). Interestingly, in this experimental paradigm, 10 μM of compound 78 increased agonist efficacy at concentrations above 10 nM (efficacy vehicle: 110,901 (95% CI: 104,175–117,694); efficacy with 10 μM of compound 78: 142,214 (95% CI: 132,594–152,109); *P* < 0.0001 for pEC_50_ and efficacy comparison). One potential explanation could be that the addition of 10 µM compound 78 to highly constitutively active GPR183 alters receptor-state occupancy. As such, subsequent agonist addition, particularly at higher agonist concentrations, elicits a larger Gi response than agonist alone relative to the receptor’s constitutive activity level. The data point to a visibly higher constitutive activity at baseline, prior to ligand incubations under the experimental conditions used for the 10 µM compound 78 competition assay (*P* = 0.096; Supplementary Fig. 1c and Fig. 2e). Here, the agonist reached higher efficacy in the presence of 10 μM of compound 78. We propose that this effect can originate from a relief of constitutive signalling that limits the agonist’s dynamic range and/or stabilization of receptor conformations with enhanced ligand-dependent Gi responsiveness. In this experimental paradigm from Fig. 2d, lower basal Gi signalling prior to compound 78 incubation can subsequently lead to an increased compound 78 efficacy and reduced 7α,25-dihydroxycholesterol efficacy at 50 nM. The representative kinetic plots from the 10 µM of compound 78 Gi ebBRET-based competition assays can be found in Supplementary Fig. 1d.

To validate the findings pertaining to the ability of compound 78 to decrease constitutive activity of GPR183, and to simultaneously assure that these results are not due to intrinsic assay artefacts, we have used an orthogonal methodology to measure activation of Gi, employing a Gi-CASE biosensor based on Nluc and cpVenus^40^. In this assay paradigm, a decrease in BRET reflects a receptor-mediated heterotrimeric G protein dissociation. We have confirmed findings from the ebBRET-based setup with a Gi-CASE-based setup with a pIC_50_ = 6.12 (95% CI: 5.24– 7.12) for 15 min incubation with compound 78 (Supplementary Fig. 1e).

Finally, we assessed the ability of compound 78 to inhibit recruitment of β-arrestin2 to the agonist-stimulated receptor using an indirect ebBRET setup (15 min agonist stimulation) that measures the energy transfer between β-arrestin2-Rluc2 and membrane-anchored rGFP-CAAX, which in turn monitors the proximity of this scaffold protein to membrane-embedded and agonist-exposed GPR183. Here, we found that 10 min incubation with 1.0, 3.0 or 10 μM of compound 78 reduced the apparent potency and efficacy of the subsequent agonist response in a concentration-dependently manner (*P* = 0.0032 for pEC_50_ and efficacy comparison for 10 μM of compound 78; Fig. 2f). This reduction in β-arrestin2 efficacy, which is consistent with an insurmountable antagonism-like profile, occurred despite relatively stable recruitment kinetics across agonist concentrations (Supplementary Fig. 1f), indicating a constrained maximal response rather than altered temporal signalling dynamics. Such an effect may reflect incomplete re-equilibration during the pre-incubation period, whereby slow dissociation of compound 78 limits agonist access to β-arrestin2-competent receptor populations. Alternatively, these data may indicate that overexpressed and compound 78-bound GPR183 adopts receptor conformations that are intrinsically less permissive for β-arrestin2 engagement upon agonist stimulation. Taken together with previous data demonstrating Gi protein activation, these results support the conclusions that compound 78 can modulate both Gi and β-arrestin2-responses of GPR183.

### Compound 78 targets GPR183

**Fig. 3:**
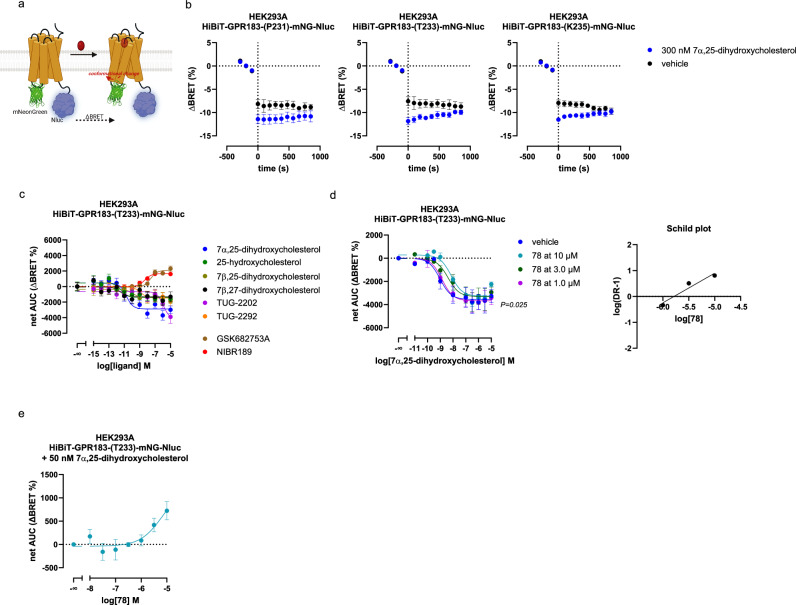
The GPR183 conformational sensor allows pharmacological profiling of receptor ligands. **a** The schematic of the HiBiT-GPR183-mNG-Nluc conformational sensor. Created in BioRender. Kozielewicz, P. (2025) https://BioRender.com/b51zmf6. **b** Agonist stimulation of the three sensors. The 15-min kinetic data are presented as ΔBRET % (over the three baseline reads); vehicle (1.0% DMSO) was not subtracted. The data are shown as mean ± SEM of three biological replicates. **c** GPR183 ligands were used to validate HiBiT-GPR183-(T233)-mNG-Nluc sensor in stably overexpressing HEK293A cells. For the agonists, basal BRET was recorded three times, followed by the addition of the compounds (15 min). For the inverse agonists, the cells were incubated (5 min) with NIBR189 or GSK682753A, and the basal BRET was measured three times. Next, 50 nM of 7α,25-dihydroxycholesterol was added, and the plate was measured (15 min). The data are shown as mean ± SD of three (NIBR189, GSK682753A), four (25-hydroxycholesterol, TUG-2202, TUG−2292), five (7β,25-dihydroxycholesterol, 7β,27-dihydroxycholesterol) or six (7α,25-dihydroxycholesterol) biological replicates. Data from the individual biological replicates were pooled, and the net AUC of ΔBRET % ± SD for each ligand concentration was calculated. Next, data for vehicle (1.0% DMSO) were subtracted, and the final SD was calculated using error propagation. **d** Left: The concentration response curve of 7α,25-dihydroxycholesterol ± 10, 3.0, 1.0 μM of 78 or vehicle (0.1% DMSO). Compound 78 or the vehicle was added, and the plate incubated for 10 min at 37 °C. Three baseline measurements were read and followed by the addition of agonist (15 min). The data are presented as net AUC of ΔBRET % (over the three baseline measurements) of four (compound 78 at 3 μM) or five biological replicates; vehicle (1.0% DMSO) was subtracted. Differences in logEC₅₀ values and in the top or bottom plateaus of the curves were analysed using an *F*-test (one-sided, *P* = 0.025). Right: Schild analysis. **e** The concentration response curve of compound 78. Compound 78 was added (10 min), followed by the addition of 50 nM 7α,25-dihydroxycholesterol (15 min). The data are presented as net AUC of ΔBRET % (over the 10 min-long read); vehicle (1.0% DMSO) was subtracted. The data are shown as mean ± SEM of three biological replicates. Source data are provided as a Source Data file.

In the absence of a reliable and direct ligand-binding assay for GPR183 in living cells, we hypothesized that the assessment of ligand-induced conformational dynamics could be a suitable proxy for quantifying drug–receptor interaction. To analyse the action of GPR183 ligands, including compound 78, in greater detail, we therefore generated a conformational sensor based on intramolecular BRET between mNG located in the ICL3 and Nluc fused to the C-terminus of the receptor (Fig. 3a). First, we screened three potential insertion sites for the mNG in the ICL3, following P231, T233, or K235. While all three sensors responded to a 15 min agonist stimulation by a similar reduction in BRET (Fig. 3b), and all of them were in principle suitable for the pharmacological validation of compound–receptor interaction in a living cell system, the T233 sensor had the biggest apparent signal window and a relatively stable temporal resolution. Thus, this HiBiT-GPR183-(T233)-mNG-Nluc construct was selected for further investigations. In a HEK293 cell line stably expressing the T233 biosensor, this sensor variant exhibited an acceptable, albeit not high-throughput screening (HTS)-applicable, *Z*-factor = 0.28 ± 0.13 (Supplementary Fig. 2a). The sensor was also readily trafficked to the cell membrane (Supplementary Fig. 2b). Nevertheless, as shown for some other tags (e.g. CFP^41,42^), introduction of the mNG tag into the ICL3 likely compromises the constitutive and ligand-stimulated ability of GPR183 to activate Gi as shown for an ebBRET Gi assay-compatible HA-GPR183-(T233)-mNG2_(11)_-HiBiT sensor in which the ICL3 was modified with a much smaller tag (Supplementary Fig. 2c and d). On the other hand, a corresponding to HiBiT-GPR183-(T233)-mNG-Nluc construct sensor based on Halo and Nluc did not respond to the agonist stimulation (Supplementary Fig. 2e). Importantly, the GPR183 sensor was able to respond to endogenous and synthetic ligands of different potencies and efficacies^7–10,23,31^ (Fig. 3c). The full agonist 7α,25-dihydroxycholesterol displayed very high potency and the greatest efficacy (pEC_50_ = 10.74 (95% CI = 9.59–11.53); efficacy = −2881 (95% CI = −2317 to −3521)). In contrast, the other three oxysterols, 25-hydroxycholesterol ((pEC_50_ = 11.39 (95% CI = 10.10–12.36); efficacy = −1402 (95% CI = −1029 to −1792)), 7β,25-dihydroxycholesterol ((pEC_50_ = 9.92 (95% CI = 9.10–10.91); efficacy = −1401 (95% CI = −1155 to −1653)) and 7β,27-dihydroxycholesterol ((pEC_50_ = 11.51 (95% CI = 9.44–14.98); efficacy = −1318 (95% CI = −823 to −1828)) showed a high apparent potency but with much lower efficacy. These findings suggest that prolonged and efficacious stabilization of the active GPR183 conformation may be required to elicit full agonistic behaviour in functional assays. Along these lines, the original publications^7,8^ evaluating multiple oxysterols across several assays (Ca²⁺ mobilization, GTPγS binding, cAMP, radioligand binding) did not establish a consistent potency/affinity rank order among these endogenous compounds (e.g., due to oxysterol metabolism in cellular systems), with 7α,25-dihydroxycholesterol remaining the most efficacious agonist. Next, from the two synthetic agonists, TUG-2292 was visibly more potent but less efficacious (pEC_50_ ≈ 7 and efficacy at 10 μM ≈ 50% of 7α,25-dihydroxycholesterol) than TUG-2202 (pEC_50_ = 6.2 and efficacy at 10 μM ≈ 70% of 7α,25-dihydroxycholesterol) in the GPR183-mediated Gi activation assay^9,10^, which is in line with our biosensor data (TUG-2292: pEC_50_ = 8.89 (95% CI = 7.75–10.83); efficacy = −1476 (95% CI = 1046 to −1931); TUG2202: pEC_50_ = 5.91 (95% CI = 4.99–6.72); efficacy = −4288 (95% CI = −2992 to −7622)). For the inverse agonists, we report that NIBR189 has a higher potency than GSK682753A (NIBR189: pIC_50_ = 8.34 (95% CI = 8.03–8.68); efficacy = 1670 (95% CI = 1523−1820); GSK682753A: pIC_50_ = 7.59 (95% CI = 7.05–8.13); efficacy = 2103 (95% CI = 1683−2534)), which also mirrors previous data from GTPγS studies (NIBR189 p*K*_b_ = 8.6 from ref. ^23^, GSK682753A p*K*_b_ = 7.2 from ref. ^26^). Altogether these results provide validation that the conformational GPR183 biosensor shows differential responses to established GPR183 ligands with different pharmacological profiles.

Given that the conformational dynamics of the receptor is the first direct and very proximal event that follows agonist binding, we interpret that for this experimental setup the potency of that ligand would also be a good approximation of its affinity (EC_50_ ≈ *K*_d_)^43^. Employing the HiBiT-GPR183-(T233)-mNG-Nluc sensor in transiently overexpressing HEK293A cells, we then analysed the effect of compound 78 on the conformational dynamics of the receptor and reported that, upon 10 min of 1.0, 3.0 or 10 μM of compound 78 pre-incubation, the increased fixed concentrations of the ligand, right-shifted (*P* = 0.025 for 10 μM) the agonist concentration–response (15 min) curve with the Schild slope = 1.15, indicating the reversible competition, with p*K*_b_ = 5.79 (Fig. 3d). Furthermore, in a setup, where a full concentration range of compound 78 was used in the presence of 50 nM 7α,25-dihydroxycholesterol (10 min pre-incubation of compound 78 followed by a 15 min stimulation with the agonist), the curve did not reach saturation at 10 μM concentration of compound 78, and thus the pharmacological parameter represents an apparent value calculated with a large confidence interval (ambiguous pIC_50_ = 5.16 (95% CI = ambiguous–6.07)) (Fig. 3e).

### Molecular dynamics simulation and in vitro validation of the compound 78 binding site

**Fig. 4:**
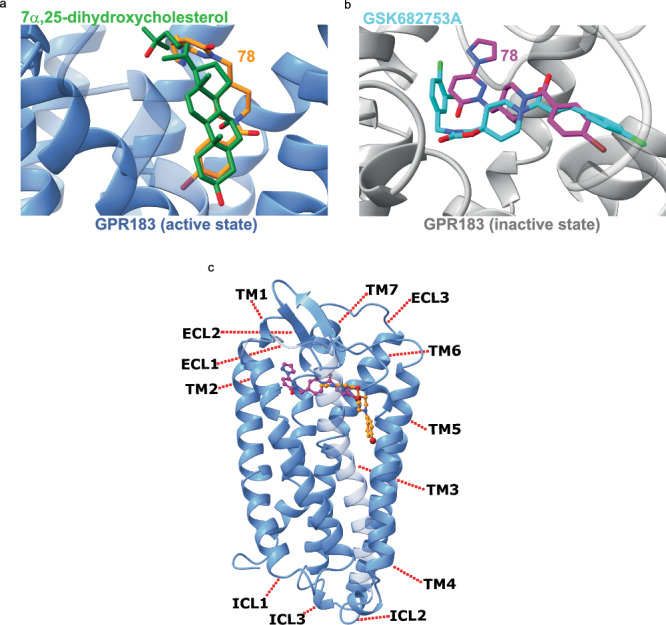
MD-predicted binding poses of compound 78 in the active- and inactive-like GPR183. **a** Overlap between compound 78 and 7α,25-dihydroxycholesterol from the MD simulations using the “active” GPR183. **b** Overlap between compound 78 and GSK682753A from the MD simulations using the “inactive” GPR183. **c** Overview of compound 78 binding mode in its orthogonal and parallel orientations.

To provide an atomistic view of its inverse agonism, molecular dynamics (MD) simulations were performed on GPR183 in complex with compound 78. A central part of this investigation was to understand how compound 78 is recognized by the active (PDB ID: 7TUZ) and inactive (PDB ID: 7TUY)^2^ states of GPR183. These simulations were attained employing the Amber24 software (Amber manual, Amber 2025, University of California, San Francisco). The starting conformations for both complexes were obtained through molecular docking calculations employing AD4-GPU^34^ and subjected to 1.5 μs MD simulations (in three replicas each). Analysis of the ligand root mean square deviation (RMSD) demonstrated that the two poses of compound 78 in the active and inactive state of GPR183 were stable over the full 1.5 μs MD simulation time (Supplementary Fig. 3a–c) and Supplementary Fig. 4a–c), respectively. While the ligand in the active state of the receptor remained stable from the beginning, the pose in the inactive state underwent a conformational relocation in the very initial steps (~10 ns out of 1.5 μs) of the simulation before settling into an equally stable plateau for the rest of the trajectory. The binding poses of compound 78 in the active and inactive states of the receptor closely resemble those of the experimentally determined 7α,25-dihydroxycholesterol (agonist) and GSK682753A (inverse agonist) (Fig. 4a, b), respectively.

In the active conformation, compound 78 binds deep within the central cavity of the GPR183 transmembrane domain (Fig. 4a). It adopts an elongated, parallel orientation (relative to the transmembrane bundle) to establish contacts with TM3 throughout TM7 (Fig. 4c). This pose is stabilized by key aromatic and polar interactions. Specifically, the *p*-bromophenyl moiety forms an extended π-π interaction with the Y116^3.37^ side chain (TM3), which is strengthened by the electron-withdrawing bromine. Simultaneously, the pyrazole moiety forms a hydrogen bond with the Y260^6.51^ side chain, while the compound’s carbonyl oxygen forms hydrogen bonds with both C201^5.43^ and H261^6.52^ (Supplementary Fig. 3d and e).

Conversely, in the inactive state, compound 78 adopts a dramatically different pose (Fig. 4b). It occupies the upper region of the binding pocket, adopting an orthogonal orientation to the transmembrane bundle and overlapping the experimental binding orientation of GSK682753A (Fig. 4c). This pose is stabilized by a separate set of interactions (Supplementary Fig. 4d and e). The *p*-bromophenyl moiety points toward TM5, forming π–π interactions with F187 (ECL2). Additionally, the pyridazinone core and pyrazole moiety are well-positioned within an aromatic pocket formed by Y91^2.64^, Y38^1.39^, and H291^7.36^ (Supplementary Fig. 4e). Hydrogen bonds further anchor this pose to both the Y184 backbone NH (ECL2) and the Q287^7.32^ side chain (TM7) (Supplementary Fig. 4d and e).

**Fig. 5:**
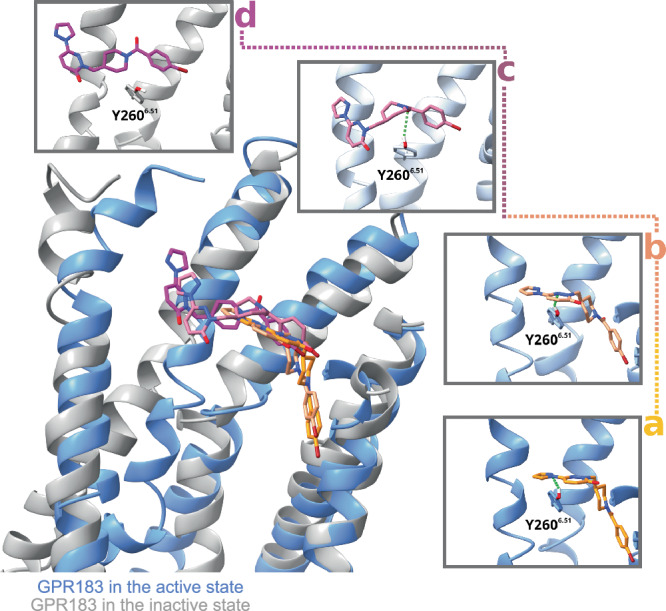
Representative snapshots from the targeted molecular dynamics (TMD) simulation of compound 78 transitioning within GPR183. The sequence (**a–d**) illustrates the ligand’s translocation from the active conformation a towards the outer, inactive-state binding pose d. The simulation highlights how compound 78, while moving outward, maintains contact with Y260^6.51^(TM6) via a “hand-off” mechanism. This interaction effectively drags the residue along, disrupting the stabilizing inter-helical interactions with Y112^3.33^ (TM3) and thereby releasing the constraint on TM3 to allow receptor inactivation.

To investigate how compound 78 translocates from a parallel to an orthogonal pose, thereby inducing receptor inactivation, targeted molecular dynamics (TMD) simulations were employed. As described above, in the starting active conformation, the pyrazole moiety of compound 78 (Fig. 5a) forms a tight hydrogen bond with Y260^6.51^ (TM6). This Y260^6.51^ is, in turn, in contact with Y112^3.33^ (TM3). This Y112^3.33^–Y260^6.51^ inter-helical contact appears to act as a “lock,” stabilizing the receptor in its active form by preventing TM3 from relocating (Supplementary Fig. 5a and b). As the TMD simulation progressed, compound 78 moved from the deep cavity towards the outer, inactive-state-binding pose. Crucially, as compound 78 moved, it did not break its interaction with Y260^6.51^. Instead, it performed a “hand-off”, with this residue’s polar contacts switching from the ligand’s pyrazole to its pyridazinone nitrogen and carbonyl oxygen. This relocation, with compound 78 effectively dragging Y260^6.51^ along for the movement, led to the loss of the Y112^3.33^-Y260^6.51^ inter-helical constraint. This frees the Y112^3.33^ side chain, allowing TM3 to undergo the pivotal rearrangement necessary for the receptor to adopt its inactive conformation.

According to this model, if Y260^6.51^ remains locked to Y112^3.33^, the TM3 rearrangement is prevented, and the receptor is stabilized in its active state, which should account for a compound’s agonist behaviour. The simulations revealed a key role for Y260^6.51^ in enabling the compound 78-induced conformational switch toward the inactive receptor state. Interestingly, a similar pattern of consecutive hydrogen bond acceptor atoms is also present in the reported NIBR189 and GSK682753A inverse agonists, and it is tempting to postulate that the same “hand-off” mechanism is responsible for the receptor inactivation by this ligand. In contrast, the endogenous agonist 7α,25-dihydroxycholesterol, despite having a 25-hydroxyl group, lacks the necessary hydrogen bond acceptors in its predominantly apolar scaffold, thereby being unable to perform this “hand-off” and translocate.

To experimentally validate the binding mode predicted by our MD studies, we sought to confirm the importance of the key hydrogen-bonding residues. As the experiments would be performed on an overexpressed, highly constitutively active receptor, we focused on the predictions from the MD simulations on the active conformation. To this end, we hypothesized that if C201^5.43^, Y260^6.51^, and H261^6.52^ are essential for the binding of compound 78, then mutating these residues should alter the compound’s ability to inactivate GPR183 and/or compete with 7α,25-dihydroxycholesterol. To test this, we engineered three receptor mutants, C201A^5.43^, Y260F^6.51^, and H261A^6.52^, and subjected them to experiments assessing basal and agonist-induced receptor-mediated Gi activation as well as agonist-induced conformational change in the receptor.

**Fig. 6:**
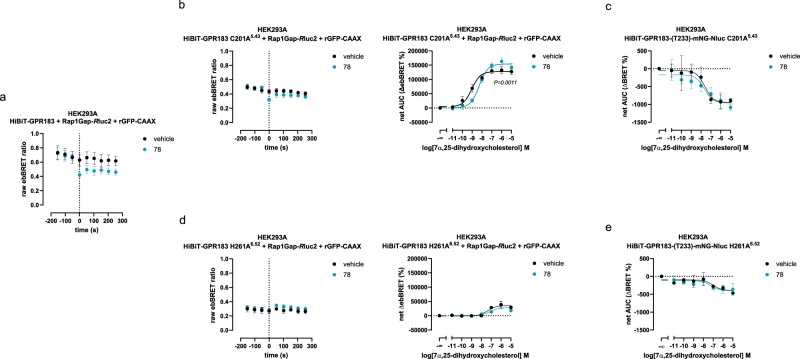
The key interactome of compound 78 in the GPR183: in vitro analysis. **a** Addition of 10 μM of 78 (time = 0) leads to a reduction in the ebBRET for WT. The data are presented as a raw ebBRET ratio; vehicle (1.0% DMSO) was not subtracted. The data are shown as mean ± SEM of four biological replicates. **b Left**: Addition of 10 μM of 78 weakly decreases ebBRET for C201A^5.43^. The data are shown as mean ± SEM of four biological replicates; vehicle (0.1% DMSO) was not subtracted. **Right**: Preincubation (6 min) with 10 μM of 78 modified agonist-simulated C201A^5.43^-mediated Gi activation. The data are presented as net AUC of ΔebBRET % (over three baseline measurements) from 15 min measurements; vehicle (1.0% DMSO) was subtracted. Differences in logEC₅₀ values and in the top or bottom plateaus of the curves were analysed using an *F*-test (one-sided, *P* = 0.0011). The data are shown as mean ± SEM of four biological replicates. **c** The concentration response curve of 7α,25-dihydroxycholesterol on the HiBiT-GPR183-(T233)-mNG-Nluc C201A^5.43^ ± of 10 μM of compound 78. Compound 78 or vehicle was added, and the BRET signal was measured (10 min), followed by the addition of different concentrations of 7α,25-dihydroxycholesterol (15 min). The data are presented as net AUC of ΔBRET % (over the 10 min-long read); vehicle (1.0% DMSO) was subtracted. The data are shown as mean ± SEM of three biological replicates. **d Left**: Addition of 10 μM of compound 78 did not modify ebBRET for H261A^6.52^. The data are shown as mean ± SEM of four biological replicates; vehicle (0.1% DMSO) was not subtracted. **Right**: Preincubation (6 min) with 10 μM of compound 78 did not modify agonist-simulated H261A^6.52^-mediated Gi activation. The data are shown as mean ± SEM of four biological replicates. **e** The concentration response curve of 7α,25-dihydroxycholesterol on the HiBiT-GPR183-(T233)-mNG-Nluc H261A^6.52^. The data are presented as net AUC of ΔBRET % (over the 10 min-long read); vehicle (1.0% DMSO) was subtracted. The data are shown as mean ± SEM of three biological replicates. The mutations also impact the agonist binding kinetics; only the first three min were used in the analysis. Source data are provided as a Source Data file.

C201A^5.43^ mutant presents itself with a lower constitutive activity than the GPR183 WT (Fig. 6a and b). Here, compound 78 led to a less pronounced reduction in the constitutive activity of this GPR183 variant in the Gi activation assay in comparison with the WT (Fig. 6b). Next, we sought to measure the activity of compound 78 in this experimental paradigm for GPR183 C201A^5.43^ by pre-applying compound 78 (6 min) prior to agonist stimulation (15 min). Similarly to WT, we observed a relatively weak but statistically significant difference in agonist potency and efficacy between vehicle- and 10 µM compound 78-treated conditions (pEC₅₀ = 9.04 (95% CI: 8.66–9.42); efficacy = 127,214 (95% CI: 115,911–138,728) vs. pEC₅₀ = 8.29 (95% CI: 8.00–8.55; efficacy = 154,024 (95% CI: 141,973–166,286)); *P* = 0.0011 for pEC_50_ and efficacy comparison; Fig. 6b). To further complement these findings, we evaluated whether 10 µM of compound 78 could modulate agonist-induced conformational changes in the GPR183 C201A^5.43^ mutant using the GPR183-(T233)-mNG-Nluc sensor. In this experimental setup, compound 78 did not significantly modulate agonist potency in this context (pEC_50_ = 7.61 (95% CI: 6.74–8.57) vs. pEC_50_ = 7.67 (95% CI: 6.30–9.42) for vehicle and compound 78-treated conditions, respectively; *P* = 0.96; Fig. 6c).

In the case of the H261A^6.52^ mutant, 10 µM of compound 78 failed to alter the dramatically reduced constitutive activity (compared to GPR183 WT) in the Gi activation assay (Fig. 6d). Given the much lower basal activity of this mutant, we sought to confirm the apparent lack of compound 78 activity in this experimental paradigm by pre-applying the compound (6 min) prior to the agonist stimulation (15 min). No significant difference was observed in agonist potency or efficacy between vehicle and 10 µM of compound 78-treated conditions ((pEC₅₀ = 7.38 (95% CI: 6.93–7.84); efficacy = 35,464 (95% CI: 28,453–42,845) vs. pEC₅₀ = 7.15 (95% CI: 6.42–8.17; efficacy = 28,041 (95% CI: 18,646–38,159)); *P* = 0.2115 for pEC_50_ and efficacy comparison; Fig. 6d). To further validate these findings, we evaluated whether 10 µM of compound 78 could suppress agonist-induced conformational changes in the GPR183 H261A^6.52^ mutant using the GPR183-(T233)-mNG-Nluc sensor. Consistent with the functional Gi-based assay, compound 78 did not significantly inhibit agonist potency in this context (pEC_50_ = 6.90 (95% CI: 4.45–8.23) vs. pEC_50_ = 7.42 (95% CI: 5.83–11.75) for vehicle and compound 78-treated conditions, respectively; *P* = 0.56; Fig. 6e).

### Y260^6.51^ defines compound 78 binding and activation mechanisms of GPR183

**Fig. 7:**
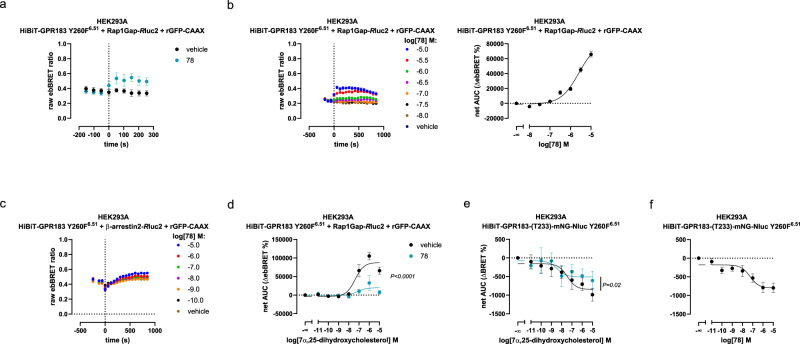
The key interactome of compound 78 in the GPR183: in vitro analysis of Y260F^6.51^. **a** 10 μM of 78 induced Y260F^6.51^-mediated Gi activation. The data are shown as mean ± SEM of four biological replicates; vehicle (0.1% DMSO) was not subtracted. **b Left**: More concentrations of compound 78 were used. The data are shown as mean ± SEM of three biological replicates. **Right**: Compound 78 induces a concentration-dependent increase in ebBRET. The data are presented as net AUC of ΔebBRET % from 15 min measurements (over three baseline measurements) and vehicle (1.0% DMSO) was subtracted. The data are shown as mean ± SEM of four biological replicates. **c** Compound 78 does not induce a measurable recruitment of β-arrestin2. The data are shown as mean ± SEM of three biological replicates. **d** Preincubation (6 min) with 10 μM of compound 78 modified agonist-simulated Y260F^6.51^-mediated Gi activation. The data are presented as net AUC of ΔebBRET % (over the three baseline measurements) from 15 min measurements; vehicle (1.0% DMSO) was subtracted. Differences in logEC₅₀ values and in the top or bottom plateaus of the curves were analysed using an *F*-test (one-sided, *P* < 0.0001). The data are shown as mean ± SEM of four biological replicates. **e** The concentration response of 7α,25-dihydroxycholesterol on the HiBiT-GPR183-(T233)-mNG-Nluc Y260F^6.51^ ±10 μM of compound 78. Compound 78 or vehicle was added, and the BRET signal was measured (10 min), followed by the addition of different concentrations of 7α,25-dihydroxycholesterol (15 min). The data are presented as net AUC of ΔBRET % (over the first 10 min-long read); vehicle (1.0% DMSO) was subtracted. Differences in logEC₅₀ values and in the top or bottom plateaus of the curves were analysed using an *F*-test (one-sided, *P* = 0.020). The data are shown as mean ± SEM of three biological replicates. The mutation also impacts the agonist binding kinetics; only the first three min were used in the analysis. **f** Compound 78 induces a concentration-dependent decrease in BRET in the conformational sensor. The data (3 min) are presented as net AUC of ΔBRET % (over three baseline measurements), vehicle (1.0% DMSO) was subtracted. The data are shown as mean ± SEM of seven biological replicates. Source data are provided as a Source Data file.

Our TMD analysis has established Y260^6.51^ as the key residue in the compound 78-induced transition of the active to an inactive GPR183 conformation. To validate these in silico predictions, we mutated Y260^6.51^ to phenylalanine to remove the hydroxyl group and thereby the predicted hydrogen bond interaction. Interestingly, in the Y260F^6.51^ mutant, the addition of 10 μM of compound 78 increased the GPR183-mediated Gi activation (Fig. 7a). To complement these data, we analysed the activity of compound 78 using a full concentration range and demonstrated that it exhibits a positive efficacy with a pEC₅₀ = 5.59 (95% CI: 5.24–5.92); Fig. 7b). However, compound 78 did not elicit recruitment of β-arrestin2 to the GPR183 Y260F^6.51^ (Fig. 7c), suggesting that it either (1) acts as a biased agonist on this receptor variant or (2) that this receptor variant is incapable of recruiting β-arrestin2. Next, a 15 min stimulation with various concentrations of 7α,25-dihydroxycholesterol following a 10 min pre-incubation with 10 μM of compound 78 resulted in strongly reduced potentiation of Gi activation compared to vehicle control while the agonist’s potency remained unchanged (pEC₅₀ = 7.38 (95% CI: 7.03–7.75) vs. pEC₅₀ = 7.14 (95% CI: 6.05–8.67); *P* < 0.0001 for pEC_50_ and efficacy comparison; Fig. 7d). Similarly, compound 78 did not significantly alter the potency of the agonist to induce the active conformation of GPR183 (pEC_50_ = 8.43 (95% CI: 6.44–9.65) vs. pEC_50_ = 7.44 (95% CI: 5.79–9.23) for compound 78 and vehicle, respectively; *P* = 0.22), but did reduce BRET_min_ (*P* = 0.02), suggesting that compound 78 stabilizes an active receptor conformation, where it competes with the orthosteric agonist and thereby reduces its maximal efficacy due to a ceiling effect (Fig. 7e). Similarly, to the measurements of the Gi activation, we applied the compound 78 in a full range of concentrations on the mNG-Nluc-based conformational sensor. Here, we detected that compound 78 induces a concentration-dependent decrease in BRET, indicative of a conformational change mirroring that of an orthosteric agonist (pEC_50_ = 7.27 (95% CI: 6.48–8.33) (Fig. 7f). Collectively, these data show that compound 78 stabilizes an active, Gi-biased conformation of GPR183 Y260F^6.51^.

Altogether, the findings from the mutagenesis analysis of GPR183 with compound 78 validate the accuracy of our MD-based predictions and underscore the critical roles of C201A^5.43^ H261^6.52^and Y260^6.51^ in anchoring compound 78 within the GPR183 orthosteric binding pocket. To gain further insights into the mode of action of compound 78 on Y260F^6.51^, we designed and obtained an analogue compound (herein referred to as compound 105 (see Supplementary Fig. 6a for structural formula). This ligand differs from compound 78 by replacing its 1H-pyrazol-1-yl substituent with a 1H-pyrrol-1-yl one. This modification was designed to abrogate the initial hydrogen bond interaction with Y260^6.51^ (present in the active state, Supplementary Fig. 3e) and test the viability of the proposed “hand-off” mechanism for receptor inactivation. The central hypothesis was that if this hand-off mechanism is not viable, the loss of this key interaction should fail to promote inactivation in the GPR183 WT, resulting in sustained agonism. This outcome would mimic the effect observed when the original ligand (compound 78) is tested against the Y260F^6.51^ mutant (which also cannot form this hydrogen bond due to the absence of the donor counterpart). Conversely, if the hand-off mechanism is valid, the cooperative presence of adjacent hydrogen bond acceptors in the ligand should counterbalance the loss of that initial interaction in the WT receptor. This would allow the receptor to still traverse its metastable conformation and proceed to inactivation. Thus, this modification was expected to disrupt potential hydrogen bonding to F260^6.51^, thereby reducing agonist efficacy on the Y260F^6.51^ mutant while retaining inverse agonism on the WT receptor. Along these lines, the data in the Supplementary Fig. 6b showed that compound 105 preserved the pharmacological profile of compound 78 on the GPR183 WT as an inverse agonist (compound 105: pIC_50_ = 6.39 (95% CI: 6.17–6.60; compound 78: 6.48 (95% CI: 6.24-6.73), *P* = 0.18) but significantly reduced its agonistic properties on the Y260F^6.51^ (Supplementary Fig 6c); (compound 105: pIC_50_ = 5.11 (95% CI: 4.51–5.46, efficacy = 39,392 (95% CI: 26932-94971); compound 78: pIC_50_ = 5.59 (95% CI: 5.24–5.92), efficacy = 82,414 (95% CI: 65,066–111,372); *P* < 0.0001). Interestingly, no differences between the compounds 78 and 105 were observed using the conformational sensor assay (Supplementary Fig 6d); (compound 105: pEC_50_ = 7.45 (95% CI: 6.29–8.67, BRET_min_ = −678.3 (95% CI: −519.0 to −880.7); 78: pEC_50_ = 7.27 (95% CI: 6.48–8.33), BRET_min_ = −800.3 (95%CI: −627.5 to −989.8); *P* = 0.57 for the comparison of both pEC_50_ and efficacy). Taken together, these data strongly support the proposed “hand-off” mechanism.

Importantly, it has been shown in the literature that Y260^6.51^ constitutes one of the several key residues in the binding pocket of GPR183 and that Y260F^6.51^ fully compromises agonist binding, severely reduces GTPγS binding, and fully blocks agonist-induced β-arrestin recruitment^11^. Here, we have already shown that application of BRET-based tools has allowed us to reliably measure agonist-induced Gi activation (ebBRET-based experiment) and conformational change as a proxy of an agonist binding (mNG-Nluc conformational sensor-based experiments) at Y260F^6.51^. To complete our pharmacological analysis of 7α,25-dihydroxycholesterol at Y260F^6.51^, we analysed agonist-mediated β-arrestin2 recruitment. As shown in Supplementary Fig. 7, Y260F^6.51^ completely abrogated agonist-induced β-arrestin2 recruitment to the receptor (similarly to the results with compound 78). All these results underline that Y260^6.51^ is a key residue not only for compound 78 and 7α,25-dihydroxycholesterol binding but also acts as a key switch in agonist-mediated signalling bias. It should be noted that, while we have shown here that the constitutive Gi activation of the GPR183 WT is higher in comparison with Y260F^6.51^ (Fig. 6a vs. Fig. 7a), at similar cell surface expression levels (Supplementary Fig. 8a), assessments of basal conformational state employing the conformational biosensors reported no differences in basal BRET comparing WT and Y260F^6.51^ across different plasmid transfection amounts, suggesting that differences in basal activation of Gi cannot be explained solely by basal receptor conformation (Supplementary Fig. 8b). Consequently, compound 78 and its analogue compound 105 display indistinguishable behaviour in the conformational sensor assay. Because it is predicted that, similarly to the WT, this sensor is unlikely to couple to Gi (Supplementary Fig. 2c), the system reports global receptor conformation rather than the specific coupling-competent state.

Finally, the key role of Y260^6.51^ could not be observed with NIBR189. Even with the weak basal activity of the Y260F^6.51^ variant, NIBR189 still retained its inverse agonist properties on this variant emphasizing its different mode of action (Supplementary Fig. 9).

### Compound 78 inhibits GPR183-mediated lymphocyte migration

**Fig. 8:**
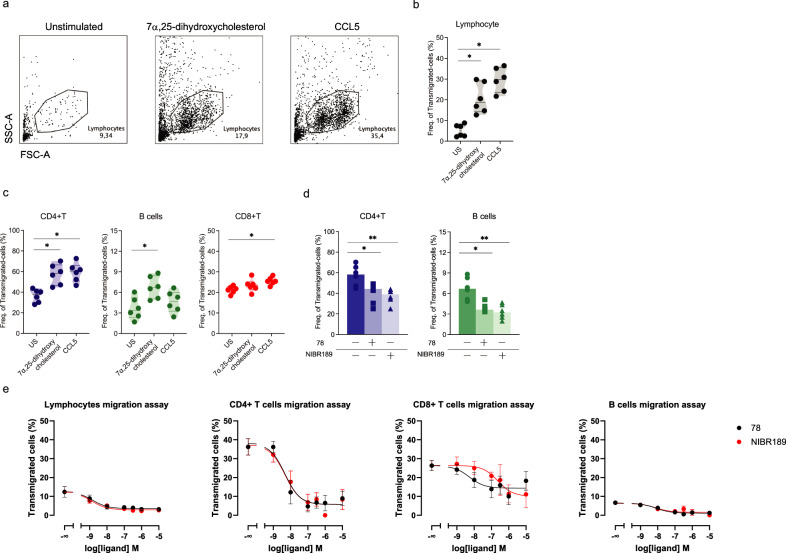
Compound 78 inhibits GPR183-mediated lymphocyte, particularly CD4+ T and B cells, migration. **a** Flow cytometric plot representing transmigrated lymphocytes of one healthy control (HC) after stimulation with 10 μM 7α,25-dihydroxycholesterol (0.1% DMSO final concentration) and CCL5 (50 ng/mL), and unstimulation (US). **b** Frequencies of migratory lymphocytes (*n* = 6). *P* = 0.043 for US vs. 7α,25-dihydroxycholesterol, *P* = 0.015 for US vs. CCL5. **c** Frequencies of migratory CD4+ T, B and CD8+ T cells in HC (*n* = 6) after stimulation. CD4+ T cells: *P* = 0.045 for US vs. 7α,25-dihydroxycholesterol, *P* = 0.011 for US vs. CCL5; B cells *P* = 0.033 for US vs. 7α,25-dihydroxycholesterol; CD8+ T cells: *P* = 0.011 for US vs. CCL5. **d** Frequencies of transmigrated CD4+ T (left panel) and B (right panel) cells after treatment with compound 10 μM of compound 78 and 10 μM NIBR189 in 7α,25-dihydroxycholesterol-stimulated (10 μM) samples from HC (*n* = 6). CD4+ T cells: *P* = 0.014 (*), *P* = 0.005 (**); B cells: *P* = 0.045 (*), *P* = 0.004 (**). All comparisons were analysed by Kruskal–Wallis test and *P* values were corrected by Dunn’s test for multiple comparisons. Only statistically significant *P* values < 0.05 are presented. **e** Compound 78 and NIBR189 induce a concentration-dependent reduction in the 7α,25-dihydroxycholesterol-induced (100 nM) migration of CD4+, B and CD8+ cells. Data come from five biological replicates and are presented as mean ± SEM. Source data are provided as a Source Data file.

Furthermore, we investigated the effect of compound 78 on primary lymphocyte migration using an in vitro migration assay (Fig. 8a). Stimulation with 7α,25-dihydroxycholesterol induced lymphocyte migration (*P* < 0.05), with a magnitude similar to that observed with the positive control CCL5 (*P* < 0.05), in comparison to unstimulated conditions (US) (Fig. 8b). Next, we performed a sub-analysis of the transmigrated cell compartments. Stimulation with 7α,25-dihydroxycholesterol induced transmigration of CD4+ T cells and B cells (*P* < 0.05), while the addition of CCL5 only promoted migration of T (CD4+ and CD8+) cells (*P* < 0.05) (Fig. 8c). This is consistent with the relative expression of GPR183 at the surface of CD4+ and B cells (Supplementary Fig. 10a).

The addition of compound 78 reduced the proportion of transmigrated lymphocytes to the same level as NIBR189 (Supplementary Fig. 10b, left), as compared to the conditions without inhibitor (*P* < 0.05). Of note, treatment with these compounds did not significantly affect CCL5-mediated migration (Supplementary Fig 10b, right) nor cell viability (Supplementary Fig. 10c). Furthermore, this equivalent effect was observed across specific cell types as both compound 78 and NIBR189 reduced the migration of CD4+ T and B cells but had no impact on CD8+ T cells (Fig. 8d and Supplementary Fig. 10d). Having established that 10 μM of compound 78 inhibits GPR183-mediated primary migration of lymphocytes, particularly CD4+ T and B cells, to the same extent as 10 μM NIBR189, we expanded our analysis by performing a concentration-dependent inhibition of 7α,25-dihydroxycholesterol-induced migration of PBMCs. Here, we used the agonist at a lower concentration than in the initial migration experiments (100 nM), which is within the range of oxysterols’ levels reported in human plasma^15^. The results showed that the two synthetic ligands had a similar potency on overall lymphocyte migration (lymphocytes: compound 78: pIC_50_ = 8.82 (95% CI: 8.07–9.48); NIBR189 pIC_50_ = 8.89 (95% CI: 8.00–9.54); *P* = 0.89); (Fig. 8e). When focusing on the CD4+, we did not detect any differences between the ligands’ potencies (CD4+ cells: compound 78: pIC_50_ = 8.41 (95% CI: 7.97–8.83); NIBR189 pIC_50_ = 8.17 (95% CI: 7.57–8.76); *P* = 0.50 (Fig. 8e). Interestingly, whereas neither compound showed any effects on CD8+ cell migration at the higher agonist concentration (Supplementary Fig. 10d), compound 78 tended to be more potent than NIBR189 in the concentration response assays (Fig. 8e). However, these differences in ligand’s potencies did not reach statistical significance (CD8+ cells: compound 78: pIC_50_ = 8.28 (95% CI: 6.69–10.07); NIBR189 pIC_50_ = 6.63 (95% CI: 5.47–8.33); *P* = 0.10). Next, the two compounds were also equally potent in blocking GPR183-mediated migration of B cells: compound 78: pIC_50_ = 7.98 (95% CI: 7.27–8.74); NIBR189 pIC_50_ = 8.29 (95% CI: 6.25–9.27); *P* = 0.62); (Fig. 8e). Importantly, inhibitory effects on cell migration were not due to compounds’ toxicity (Supplementary Fig. 10e).

Because these migration assays were performed within a few hours and using isolated PBMCs, where metabolic turnover is negligible, the observed pharmacological similarities are unlikely to result from differences in compound stability or bioavailability. To further confirm that neither ligand is subject to metabolic degradation, we evaluated their stability in primary human liver spheroids. Primary human liver spheroids remain metabolically active in culture and exhibit a full complement of phase I and phase II metabolic enzymes, which allows for the exact determination of hepatic clearance even of low-clearance compounds^44–46^. To confirm metabolic activity in the given experiment, we incubated the spheroids with the CYP3A4 probe substrate midazolam and observed time-dependent formation of 1OH-midazolam throughout the incubation period (Supplementary Fig. 11a). In parallel, we incubated the 3D liver cultures with NIBR189 and compound 78. Importantly, concentrations of these test compounds remained stable for at least 72 h, demonstrating that the compounds are not subject to extensive metabolism by hepatic phase I or phase II enzymes, including Cytochrome P450s (CYPs), carboxylesterases (CES), UDP-glucuronosyltransferases (UGTs), and sulfotransferases (SULTs) (Supplementary Fig. 11b and c). Next, to complement in vitro metabolic stability data, and our initial BRET-based selectivity assays (against overexpressed SMO, β_2_AR, CB_1_R and endogenous Gi and Gs-coupled GPCRs in HEK293A cells) as well as to provide an initial starting point for potential future optimization, compound 78 (10 μM) was screened against a panel of GPCRs, Lck kinase, ion channels, enzymes, and transporters using the Eurofins SafetyScreen18 Core Panel^47^. Importantly, no off-target activity was observed across the tested GPCRs (Supplementary Data 1). The observed compound 78 binding to hERG remained limited, with a predicted IC_50_ substantially above 10 µM, which, even taking into account differences in assaying methodologies^48^, compares very favourably with that of previously identified lead compounds^24,25^, indicating that compound 78, which in the current study is used as a tool to interrogate GPR183 pharmacology, represents a very good starting point for potential future clinical development. As the binding of compound 78 to an allosteric site of the GABA_A_ receptor was observed, any future structure optimization efforts should take this into consideration. Importantly, this does not affect the mechanistic interpretations, the key role of Y260^6.51^ in GPR183, and the conclusions drawn from this study. Likewise, the data on the interaction of compound 78 with GPR183 are unaffected, as HEK293A cells do not express the GABA_A_ receptor. Moreover, in physiological settings, the GABA_A_ receptor is associated with the central nervous system, whereas GPR183 is primarily expressed on immune cells. In addition, because compound 78 is predicted to have only moderate brain penetration, any potential interaction with GABA_A_ receptors in the brain would likely be limited. Further, calculations for predicted absorption, distribution, metabolism, and excretion (ADME) properties were performed employing the QikProp software (Schrödinger Release 2025-4: QikProp, Schrödinger, LLC, New York, NY, 2025). In addition to predicting molecular properties, QikProp provides ranges for comparing each compound’s property with those of 95% of known drugs. According to these calculations, compound 78 displays no violations of the ranges recommended for each descriptor or property (Supplementary Table 2).

## Discussion

In this study, we not only applied and developed several tools but also introduced a chemical scaffold—compound 78—that enabled detailed investigations of GPR183 activation mechanisms. Functional assays in overexpressed HEK293 cells revealed that compound 78 significantly modulates both constitutive and agonist-induced Gi signalling and agonist-stimulated β-arrestin2 recruitment to overexpressed GPR183 with moderate potencies. In more physiological conditions, compound 78 was much more potent, exhibiting low-nanomolar IC₅₀ activity in blocking agonist-activated GPR183-mediated human primary immune cell migration. Moreover, the combined use of these methods and application of the compound 78 as a tool compound enabled us to uncover mechanistic details of GPR183 activation, highlighting Y260^6.51^ as a key residue. To this end, Y260^6.51^ is proposed as a molecular switch driving an inverse agonist-to-agonist switch of compound 78 and agonist-stimulated receptor signalling bias. Importantly, the relevance of this residue in this context has not yet been demonstrated by others using different assay formats and would not have been evident with NIBR189.

Specifically, this study reports the discovery of a GPR183 inverse agonist, identified through an integrated AI-driven SBVS strategy and validated using several biophysical tools, including a conformational BRET-based biosensor. GPR183, a Gi-coupled chemotactic receptor with roles in immune cell trafficking and disease pathogenesis, remains an underexploited drug target despite mounting evidence linking its activity to various inflammatory, autoimmune, and malignant conditions. Previous efforts to pharmacologically target GPR183 have focused on piperazine diamides and benzo[d]thiazole-based inverse agonists. Our study aimed at expanding the chemical repertoire for GPR183 ligands and identified a series of pyrazolpyridazinones, among which the most interesting one, compound 78, has activity on both Gi and β-arrestin pathways.

Here, the discovery pipeline leveraged PyRMD2Dock, a hybrid AI-docking platform designed to enrich VS hits with favourable predicted binding profiles and contact maps aligned with key GPR183 residues. This approach proved effective in identifying promising binders from a large chemical space, yielding a 20% initial hit rate from the primary 70-compound screen, and subsequently leading to the selection of compound 43 and its more efficacious analogue, compound 78. To this end, we establish that the PyRMD2Dock workflow is suitable for the identification of GPCR-targeting small molecules.

Another technological advance of this study is the development of a conformational biosensor for GPR183, constructed by fusing mNG into the ICL3 and Nluc at the C-terminus of the receptor. Among several sensor configurations tested, the T233 insertion site yielded the most robust and reliable BRET signal changes upon agonist stimulation. This tool, which serves as a reporter of compound-receptor binding in the absence of a bona fide real-time binding assay for GPR183, enabled us to directly monitor real-time, ligand-induced conformational rearrangements of GPR183 in live cells. Notably, the sensor responded to several validated GPR183 ligands in a concentration-dependent manner. The data reported with the sensor were in good agreement with previous studies, underscoring the utility of this biosensor for mechanistic studies (e.g., for 7α,25-dihydroxycholesterol, literature: p*K*_d_ = 9.34^8^; our study: conformational sensor pEC_50_ = 9.02 (transient overexpression), pEC_50_ = 10.74 (stable overexpression)). Next, the sensor allowed the competition binding studies and analysis of compound 78 binding. The application of conformational sensors to orphan GPCRs remains largely underexplored^49^, with the only two very recent notable exceptions: a Halo-Nluc-based sensor for GPR3^50^ and FlAsH-BRET-based sensor for GPR101^51^. While we have not attempted a FlAsH-based tool, in our hands, however, a GPR183-oriented sensor based on a Halo-Nluc principle failed to functionally respond to an agonist stimulation (Supplementary Fig. 2e). This discrepancy highlights the empirical challenges in engineering tailored biosensors. The failure of our Halo-Nluc construct likely stems from its architecture; the bigger HaloTag may have sterically hindered the ICL3’s movement more significantly than a smaller mNG, while the rotational freedom of its fluorophore could have averaged out the BRET signal—a limitation not present with mNG’s fixed chromophore. Ultimately, this finding underscores that the rational design of such tools must be followed by empirical validation of different architectures to identify a functional sensor.

Next, MD simulations, substantiated by mutagenesis data, further supported the predicted binding mode, highlighting key interactions with residues, e.g., C201^5.43^, Y260^6.51^ and H261^6.52^, which appear essential for ligand engagement and receptor stabilization in an inactive conformation. Moreover, MD simulations allowed us to propose the inactivation mechanism induced by compound 78 when binding to the GPR183 active state, outlining the role of specific residues lining the binding pocket in a hand-off routine taking place between the ligand and the receptor counterpart. In addition, this “hand-off” model suggests that for GPR183, a constitutively active GPCR, the development of effective inverse agonists may require a new approach. Rather than focusing solely on affinity for the inactive state, a more promising strategy might involve designing molecules that proficiently bind to the active state and are capable of inducing and triggering this proposed conformational switch.

The combined in silico and in vitro analyses were further supported by site-directed mutagenesis. It has to be noted that, consistently with the literature, H261A^6.52^ and Y260F^6.51^ impaired the pharmacological properties of the agonist^11,52^. In the case of C201A^5.43^, we have detected a decrease in agonist potency and an increase in agonist efficacy (for agonist concentration > 10 nM) in the presence of 10 μM of compound 78, similarly to WT, but the experiments with the conformational sensor of C201A^5.43^ reported the same agonist-induced response in the presence or absence of compound 78. Thus, we hypothesize that the increased efficacy towards Gi activation suggests that a short pre-incubation with compound 78 enhances the efficiency of receptor–Gi coupling also in the C201A^5.43^ background. In this scenario, the receptor reaches the same activated conformation without or with compound 78, but the downstream coupling process becomes more productive, leading to higher signalling output despite unchanged conformational dynamics. Next, focusing on the Y260F^6.51^ variant, our data indicate that compound 78 may in fact function as a partial agonist at this mutant, stabilizing an active conformation of the receptor. First, replacing Y260^6.51^ with phenylalanine converted compound 78 from an inverse agonist into an agonist, underscoring the importance of this residue for compound 78's binding mode and its efficacy profile. Second, the mutation induced a pronounced orthosteric agonist-mediated signalling bias, as the receptor selectively activated Gi-protein signalling while failing to recruit β-arrestin2, highlighting Y260^6.51^ as a critical determinant in the receptor-activation switching mechanism. In this context, it needs to be noted while 6.51 (6 × 51) position has not been ascribed globally for GPCRs as a part of the network of microswitches or other key residues important in guiding ligand potency or efficacy^53,54^, the residues at 6.51 positions are very relevant for agonist activation in serotonin 5-HT_2A_ receptor^55^, M_2_ muscarinic receptor^56^, cannabinoid CB_1_ and CB_2_ receptors^57^ as well as for agonist/antagonist switch in δ opioid receptor^58^. As such, our findings should not only contribute to deepening our understanding of the activation/inactivation mechanism of GPR183 but also to other members of Class A GPCRs.

Next, although compound 78 displayed only moderate potency (but very good efficacy) in pharmacology assays using biosensors in combination with overexpressed receptor, it very potently inhibited agonist-induced migration of human PBMCs at endogenous levels of GPR183. In this assay, compound 78 presented with similar potency as NIBR189. Immune cell migration is the key biological process governed by GPR183 and underlies its physiological and pathological roles. As such, translational evaluation of any GPR183-targeting compound must consider its impact in this context. The strong migratory blockade observed for compound 78 likely reflects system-dependent effects in the PBMC assay, where, in agreement with textbooks (see ref. ^59^, Fig. 3.19, with the description) endogenous receptor levels (small receptor reserve), shift the inverse agonist potency leftward. Not only do signalling machinery and receptor expression differ between primary PBMC, where GPR183 is endogenously expressed, and the HEK293 cell line, where GPR183 is overexpressed, but the assays for these two cell types measure very different biological phenomena with different techniques and levels of intracellular signal amplification. As such, different pharmacological profiles of the same compound across different experimental paradigms are expected^60^, and have already been reported even for GPR183 ligands. Indeed, an agonist 25-hydroxycholesterol was active in GTPγS binding and cAMP assays with approximately 100×-fold difference in potencies^8^. Similarly, the NIBR189-based inverse agonist compound 33 blocked 7α,25-dihydroxycholesterol (20 nM)-driven migration and 7α,25-dihydroxycholesterol (100 nM)-stimulated gene up-regulation with 1000–10,000× fold difference in potencies^25^. In our study, for compound 78 and GPR183, the differences in potency may also indicate that partial inhibition of GPR183-mediated Gi/β-arrestin signalling is sufficient to achieve full suppression of the migratory phenotype. While potent GPR183 inverse agonists have been developed, so far none of them has reached the drug development phase. In this context, the identification of compound 78 might present an opportunity for drug design, unencumbered by the possible constraints of the previous scaffolds. Additionally, preliminary predictions of the physicochemical features coupled with in vitro determination of metabolic stability show no evident liabilities (Supplementary Table 2 and Supplementary Fig. 11). Altogether, the capacity of compound 78 to inhibit GPR183-dependent cell migration in lymphocytes with high potency, with notable effects on CD4+ T cells, CD8+ T cells and B cells, good metabolic stability, and predicted lack of toxicity, underscores its potential functional significance in immune-related contexts and provides a rationale for its therapeutic exploration in inflammatory and autoimmune diseases.

Likewise, structural aspects of compound 78 provide some guidance for developing GPR183 modulators, in principle inverse agonists as shown already here for compound 105. Still, given the results of the GPR183 Y260F^6.51^ assays, it could be possible to engineer balanced agonists and additional biased agonists for GPR183^61^, to optimize their drug-like properties, and to establish a robust safety profile from a superior starting point.

In summary, this work combines computational approaches, chemical biology, and receptor pharmacology to deliver a potent GPR183 modulator and a biosensor system, as well as detailed insights about the GPR183 receptor activation mechanism. Together, they will lead to a more nuanced understanding of GPR183 signalling and pharmacology, and open avenues for the discovery of therapeutic agents targeting this and related GPCRs in cancer, immune, and inflammatory diseases.

## Methods

### Ethics statement

We confirm that our research complies with all relevant ethical regulations. The project involving the in vitro use of blood samples from healthy donors was approved by the Regional Ethics Committee in Stockholm (Dnr 2025-01672-01). All donors gave informed written consent, according to the Declaration of Helsinki. Handling of patient-derived cells for organotypic: the suppliers BioIVT and LifeNetHealth collected informed written consent from each donor or the subject’s legally authorized representative, and the documentation was reviewed and approved by the appropriate regulatory authorities in accordance with US Department of Health and Human Services regulations and Good Clinical Practice (ICH E6). Handling and culture of patient-derived cells was approved by the Swedish Ethics Review Authority (Etikprövningsmyndigheten) under permit number 2024-05808-01.

### Molecular docking

Cryo-EM structures of GPR183 in complex with their endogenous ligand 7α,25-dihydroxycholesterol (PDB 7TUZ) and GSK682753A (PDB 7TUY)^2^ were sourced from the RCSB PDB database and underwent preliminary adjustments for docking purposes using the protein preparation wizard integrated into the Schrödinger suite^62^. The hydrogen atoms were added and minimized, the solvent molecules were removed, and the appropriate protonation and tautomeric state of the protein’s side chains were calculated at physiological pH. Using the AutoDockTools Python^63^ scripts, the GPR183 structures were converted into the AutoDock PDBQT format, where, compared to a standard PDB file, Gasteiger charges are added to the atoms, and the torsional freedoms of the various bonds are described. Then, the receptor grid maps were calculated with the AutoGrid4 software, mapping the receptor interaction energies using every AutoDock atom type as a probe, and the docking grid box was centred on the binding site on the ligand structures (*XYZ* size of the box: 60 × 60 × 60 with a spacing of 0.375 Å). From the ZINC20 database, a set of 1 M randomly chosen compounds was selected, and the molecules’ SMILES strings were converted into 3D structures with the employment of the LigPrep (Schrödinger Release 2024-1: LigPrep, Schrödinger, LLC, New York, NY, 2024) routine in Schrödinger’s Maestro suite. Their tautomeric and protonation states were calculated at physiological pH, and the possible enantiomers were generated. The prepared molecules were exported as PDB files and converted into PDBQTs by making use of the AutoDock suite scripts^63^. For the docking calculations, attained through AD4-GPU^34^, the Lamarckian genetic algorithm (LGA) was employed, encompassing a total of 50 LGA runs. All other settings were maintained at their default values. The docking results were subsequently grouped based on the RMSD criterion, whereby solutions differing by less than 2.0 Å were considered part of the same cluster. The ranking of these clusters was determined based on the calculated free energy of binding (Δ*G*_AD4_).

### AI-enforced virtual screening

To create a machine learning (ML) prediction model, PyRMD was fed with a comma-separated file (.csv) generated by extracting the predicted lowest Δ*G*_AD4_ for each of the 1 million docked compounds randomly chosen from the ZINC20 medium-large database. This led to the construction and preparation of the training dataset. According to their predicted Δ*G*_AD4_, the compounds included in the .csv file were classified into three groups: “actives”, “inactives”, and discarded. Compounds whose predicted Δ*G*_AD4_ falls below the “activity” thresholds of −9.5, −10.0, −10.5, −10.75, and −11.0 kcal mol^−1^ were placed in the active group. Instead, those with a Δ*G*_AD4_ value higher than the “inactivity” threshold of −4.0, −4.5, −5.0, −5.5, and −6.0 kcal mol^−1^ went into the “inactives” group. Moreover, compounds whose Δ*G*_AD4_ value falls above the “activity” threshold and “below-the-inactivity” threshold were discarded. By selecting the MinHash fingerprints (MHFP) for the featurization process, and by varying the *ε* cutoff for “actives” (0.01–0.99 with a 0.10 step) and “inactives” (0.01–0.99 with a 0.10 step), 3025 different models were generated. For all the models, PyRMD returns relevant metrics to evaluate their predictive capability (i.e., TPR, FPR, *F*-Score, ROC AUC, BED ROC, PRC AUC). In this work, the selected model was chosen by maximizing the true positive rate (TPR)/false positive rate (FPR) trade-off. Once the model was generated, PyRMD was used to screen the remaining ~9 million compounds from the ZINC20 database, and it automatically returned all the compounds deemed to be active along with a confidence score of its prediction (RMD score).

### Molecular dynamics

The ligand-receptor complexes obtained from the docking experiments were used to construct a molecular dynamics system. Initially, the complexes were embedded in a 1-palmitoyl-2-oleoylphosphatidylcholine (POPC) membrane and solvated in water within an orthorhombic system with a buffer distance of 10 Å using CHARMM-GUI^64^. Additionally, the overall charge of the system was neutralized, and the salt concentration was set to 0.15 M KCl. Missing Cryo-EM proteins’ extracellular loops were added using AlphaFold^65,66^ (Uniprot^67^ code: P32249). To minimize the fixed structures and adapt the added loops to the initial proteins, 2500 steps with the Steepest Descent method followed by 5000 steps employing a Polak–Ribier conjugate gradient were applied, adding constraints on the non-reconstructed residues. The complexes underwent molecular dynamics simulation using Amber22 (Amber manual, Amber 2025, University of California, San Francisco). The general AMBER force field (gaff2)^68^ was applied to compound 78 through antechamber^69^, while the protein force field (ff19SB)^70^ was employed for the GPR183 structures to create its topology parameters, using tleap^71^. To stabilize the water-counterion systems, energy minimization was conducted. Initially, the solvent’s energy and positions were adjusted through 40,000 steps of steepest descent energy minimization, followed by an additional 10,000 steps of conjugate gradient minimization. Meanwhile, the complex and the membrane remained fixed with a constraint of 10.0 kcal mol^−1^ Å^−2^. Second, the entire system was gradually heated from 0 to 300 K over three different steps of 1 ps each with position restraints applied at constant volume. The SHAKE algorithm was utilized to constrain covalent bonds involving hydrogen atoms, with a relative geometrical tolerance of 0.00001. Then, four different NPγT-MD steps were performed for a total of 840 ps to equilibrate the systems at 300 K and 1 bar, gradually removing the constraints to relax the systems (last 500 ps without constraints). Finally, a 1.5 μs simulation was performed for each complex, maintaining a constant temperature of 300 K and pressure of 1 bar. The trajectory was updated every 5000 fs for further analysis. Analysis of the MD trajectory was attained with the cpptraj library^72^ within AmberTools23. The simulations were performed three times under the same conditions but with random seeds to verify the consistency of the observed results. Supplementary Table 3 reports all the parameters of the MD simulations. Molecular graphics and analyses performed with UCSF ChimeraX 1.10 (developed by the Resource for Biocomputing, Visualization, and Informatics at the University of California, San Francisco, with support from National Institutes of Health R01-GM129325 and the Office of Cyber Infrastructure and Computational Biology, National Institute of Allergy and Infectious Diseases).

### Targeted molecular dynamics

Targeted molecular dynamics (TMD) was performed using the Sander module of the AMBER package. Both the initial structure (active state) and the target structure (inactive state) were derived from coordinates obtained during previously performed molecular dynamics simulations. TMD simulations were conducted in the NPγT ensemble. The temperature was maintained constant at 300 K using Langevin dynamics with a friction coefficient of 1.0 ps⁻¹. The pressure was kept at 1.0 bar using the Berendsen barostat with semi-isotropic coupling. For the interactions, a 9.0 Å cutoff was used for non-bonded interactions. The particle mesh Ewald (PME) method was employed for calculating long-range electrostatic interactions. The SHAKE algorithm was applied to constrain all covalent bonds involving hydrogen atoms, allowing for an integration time step of 0.001 ps (1 fs). The TMD simulation was run by applying a force constant of 1.5 kcal mol^−1^ Å^−2^ to guide the structure towards a 0.0 Å RMSD from the target structure. The RMSD calculation and structural fit were performed using the protein backbone and the protein backbone + ligand, respectively. The simulation was run for a total of 10,000 steps, corresponding to a total duration of 10 ps. Coordinates for trajectory analysis were saved at every single step.

### In vitro cell culture

HEK293A cells (Thermo Fisher Scientific; #R70507; not authenticated in-house) were cultured in DMEM supplemented with 10% FBS (Sigma), 1.0% penicillin/streptomycin, 1.0% L-glutamine (both from Thermo Fisher Scientific) in a humidified CO_2_ incubator at 37 °C. All cell culture plastics were from Sarstedt, unless otherwise specified. Plates were not coated prior to seeding cells. The absence of mycoplasma contamination was routinely confirmed by MycoStrip’s (InvivoGen) patented isothermal PCR detecting 16S ribosomal RNA of mycoplasma in the media after 2–3 days of cell exposure.

Organotypic primary human liver microtissues were cultured as previously described by us^73^. In short, primary human hepatocytes (PHH; BioIVT, USA; not authenticated in-house) and non-parenchymal cells (NPC; LifeNetHealth, USA; not authenticated in-house) were thawed and seeded in 96-well ULA plates at a stoichiometry of 6:1 and a density of 1500 cells/well. Cells were seeded in liver culture medium (William’s E medium supplemented with 2 mM L-glutamine, 11 mM glucose, 100 units/mL penicillin, 100 μg/mL streptomycin, 10 μg/mL insulin, 5.5 μg/mL transferrin, 6.7 ng/mL sodium selenite, and 100 nM dexamethasone) supplemented with 10% foetal bovine serum (FBS). After spheroid formation, FBS was phased out, and on day 7, cells were exposed to the test compounds at a concentration of 1 μM in 100 μL of serum-free PHH medium. Medium was collected at different time points after the exposure (*t*0, 30 min, 1, 2, 24 and 72 h) and stored at −80 °C until analysis.

### DNA constructs, cloning and mutagenesis

HiBiT-GPR183 plasmid DNA was generated with Gibson cloning using a codon-optimized GPR183 from GPR183-Tango plasmid DNA (#66342 Addgene, deposited by Bryan Roth) as an insert and HiBiT-FZD_6_ with a 5-HT_3A_ signal peptide plasmid DNA as a backbone^74^. In the newly generated construct, the GPR183 insert sequence replaced the FZD_6_ insert sequence. In this construct, the N-terminally cloned HiBiT tag (GTGAGCGGCTGGCGGCTGTTCAAGAAGATTAGC) is followed by a GS linker (GGATCC, BamHI site). HiBiT-GPR183-Nluc construct was generated using Gibson cloning, inserting Nluc from Nluc-FZD_6_^75^ onto the C-terminus of HiBiT-GPR183, without a linker. HiBiT-GPR183-mNG-Nluc plasmid DNA constructs were generated using Gibson cloning, inserting mNeonGreen (mNG) from mNeonGreen-APEX plasmid DNA (#202591 Addgene, deposited by Reuben Harris) at three distinct positions into the GPR183 sequence (following P231, T233 or K235) in HiBiT-GPR183-Nluc. HA-GPR183-HiBiT was generated using HA-FZD_5_-HiBiT plasmid DNA and GPR183-Tango plasmid DNA. HA-GPR183-(T233)-mNG2_(11)_-HiBiT plasmid DNA was generated using the HA-GPR183-HiBiT and mNeonGreen2_(1-10)_/mNeonGreen2_(11)_ plasmid DNA (#82611 Addgene, deposited by Bo Huang). HiBiT-GPR183-Halo-Nluc plasmid DNA construct was generated using Gibson cloning, inserting Halo from FZD_5_-Halo-Nluc sensor^76^ in HiBiT-GPR183-Nluc. Plasmid DNA constructs encoding different receptor mutants were generated with a GeneArt Site-Directed mutagenesis kit (Thermo Fisher Scientific). HiBiT-β_2_AR plasmid DNA was generated using Gibson cloning from FLAG-SNAP-β_2_AR (Davide Calebiro, University of Birmingham). rGFP-CAAX plasmid DNA, Rap1Gap1a-Rluc2 plasmid DNA^39,77^ and β-arrestin2-Rluc2 in the pcDNA3.1(+) backbone were synthesized by GenScript. Gαs67-Rluc2, based on the published principle, was synthesized and used by us before^39,77^. HA-FZD_5_-HiBiT, HA-CB_1_R, LgBiT-CAAX^78^, HiBiT-SMO^79^ and Gi-CASE^40^ were kind gifts from Lukas Grätz (University of Bonn) and Gunnar Schulte (Karolinska Institutet). Plasmid DNA encoding an α subunit of Gi_1_ was from cDNA.org. Salmon sperm DNA (ssDNA) was from Thermo Fisher Scientific. Primer DNA sequences can be found in Supplementary Table 4. The constructs were validated by Sanger sequencing (Eurofins GATC).

### Ligands

Endogenous GPR183 agonists: 7α,25-dihydroxycholesterol (#SML0541; referred also as the GPR183 agonist throughout the study), 7β-25-dihydroxycholesterol (#700081P), 7β-27-dihydroxycholesterol (#700025P) and 25-hydroxycholesterol (#H1015) were from Sigma; synthetic GPR183 agonists TUG-2202 and TUG-2292 were characterized before^9,10^. NIBR189 (GPR183 inverse agonist) was from Tocris (#5203). GSK682753A (GPR183 inverse agonist) was from Fischer Scientific (#18793645). We designed and obtained compound 105, see Supplementary Fig. 12 for the synthesis reaction and Supplementary Data 2 for 13C-NMR, 1H-NMR and LC/MS spectra). The compounds were dissolved in DMSO at 5 or 10 mM concentrations, aliquoted, and stored at −20 °C. Each aliquot was used a maximum of three times. For experiments, 7α,25-dihydroxycholesterol and other ligands were used dissolved in 0.1% or 1.0% final DMSO concentration (see the figure legends).

Compounds selected from the virtual and the subsequent similarity search screen (hit expansion) were purchased from Mcule (www.mcule.com), dissolved in DMSO at 1–10 mM concentrations, and stored at −20 °C. Each aliquot was used a maximum of five times. The list of compounds (with the vendors' IDs) from the two screens can be found in the Supplementary Figs. 13 and 14. Spectra of the most active compounds 3, 43, 78, as well as compound 105, are attached as Supplementary Data 2.

### Gi and Gs protein activation with enhanced bystander BRET

HEK293A cells were transiently transfected in suspension using polyethylenimine (PEI, Polysciences). To measure heterotrimeric Gi protein activation/dissociation at the cell membrane interface, a total of ca. 4 × 10^5^ cells were transfected in 1 mL with 200 ng of HiBiT-GPR183 plasmid DNA constructs, 300 ng of rGFP-CAAX plasmid DNA, 100 ng of an α subunit of Gi_1_ plasmid DNA, 40 ng of Rap1Gap-Rluc2 plasmid DNA and 360 ng of ssDNA. To measure Gs protein activation at the cell membrane interface, a total of ca. 4 × 10^5^ cells were transfected in 1 mL with 200 ng of HiBiT-β_2_AR plasmid DNA constructs, 300 ng of rGFP-CAAX plasmid DNA, 50 ng of Gαs67-Rluc2 plasmid DNA and 450 ng of ssDNA. Next, transfected cells (4 × 10^4^ cells in 100 μL) were seeded onto white 96-well cell culture plates. 24 h later, the cells were washed once with 200 µL of HBSS (HyClone). Next, 70–90 μL of HBSS (depending on the assay setup) was added to the wells, and subsequently, 10 μL of coelenterazine 400a (2.5 μM final concentration, Biosynth) or 10 μL of furimazine (1:1000 final concentration, Promega) was added. The plate was incubated for 10 min, which was followed by the addition of the ligand-stimulation setups (keeping the final total solution volume at 100 mL in each well). Next, Rluc2 emission (donor, 360–440 nm, 100 ms integration time) and rGFP emission (acceptor, 505–575 nm, 100 ms integration time) were measured at 37 °C (three measurements for baseline, 10–25 min with the agonist and/or compounds—see the figure legends for detailed description for respective experimental paradigms). The ebBRET ratios were defined as acceptor emission/donor emission. The measurements were performed using a Tecan Spark microplate reader.

### Gi protein activation with Gi-CASE sensor

HEK293A cells were transiently transfected in suspension using polyethylenimine (PEI, Polysciences). To measure Gi protein activation, a total of ca. 4 × 10^5^ cells were transfected in 1 mL with 200 ng of HiBiT-GPR183 plasmid DNA constructs, 10 ng of Gi-CASE plasmid DNA and 790 ng of ssDNA. Next, transfected cells (4 × 10^4^ cells in 100 μL) were seeded onto white 96-well cell culture plates. 24 h later, the cells were washed once with 200 µL of HBSS (HyClone). Next, 80 μL of HBSS was added to the wells, and subsequently, 10 μL of furimazine (1:1000 final concentration) was added. The plate was incubated for 10 min, which was followed by the addition of the ligands. Next, Nluc emission (donor, 460–500 nm, 50 ms integration time) and Venus emission (acceptor, 520–560 nm, 50 ms integration time) were measured. The BRET ratios were defined as acceptor emission/donor emission. The measurements were performed using a Tecan Spark microplate reader.

### β-arrestin2 recruitment with ebBRET

HEK293A cells were transiently transfected in suspension using PEI. To measure β-arrestin2 recruitment to the cell membrane-expressed GPR183 in an ebBRET paradigm, a total of ca. 4 × 10^5^ cells in 1 mL were transfected with 200 ng of HiBiT-GPR183 plasmid DNA, 50 ng of β-arrestin2-Rluc2 plasmid DNA, 300 ng of rGFP-CAAX plasmid DNA and 450 ng of ssDNA. Next, transfected cells (4 × 10^4^ cells in 100 μL) were seeded onto white 96-well cell culture plates. 24 h later, the cells were washed once with 200 μL of HBSS (HyClone). Next, 70 μL of HBSS was added to the wells, and subsequently, 10 μL of furimazine (1:1000 final concentration, Promega) was added. The plate was incubated for 10 min, which was followed by the ligand addition (keeping the final total solution volume at 100 μL in each well). Next, Rluc2 emission (donor, 360–440 nm, 100 ms integration time) and rGFP emission (acceptor, 505–575 nm, 100 ms integration time) were measured at 37 °C (three measurements for baseline, 10 min with the inverse agonists, and then 15 min with the agonist). The ebBRET ratios were defined as acceptor emission/donor emission. The measurements were performed using a Tecan Spark microplate reader.

### GPR183 conformational sensors experiments

HEK293A cells were transiently transfected in suspension using PEI. To measure ligand-induced conformational change in GPR183, a total of ca. 4 × 10^5^ cells were transfected in 1 mL with 50 ng of HiBiT-GPR183-mNG-Nluc plasmid DNA constructs and 950 ng of ssDNA or 10 ng of HiBiT-GPR183-Halo-Nluc plasmid DNA and 990 ng of ssDNA. Next, transfected cells (4 × 10^4^ cells in 100 μL) were seeded onto white or black 96-well cell culture plates. 24–48 h later, the cells were washed once with 200 µl of HBSS (HyClone). Next, 70–80 μL of HBSS was added to the wells, and subsequently, 10 μL of 1:1000 final Nano-Glo substrate/furimazine (Promega) was added for the mNG-Nluc sensors or 50 μL of medium was removed and replaced with 50 μL of 1:2000 final Halo618 substrate (Promega) in the Halo-Nluc setup; the Halo-Nluc plate was washed the following day once with 200 µL of HBSS (HyClone), and subsequently, 10 μL of 1:1000 final Nano-Glo substrate/furimazine (Promega) was added. The plate was incubated for 10 min, which was followed by the addition of the ligand(s) (keeping the final total solution volume at 100 μL in each well). Next, for the mNG-Nluc sensors, Nluc emission (donor, 460–500 nm, 50 ms integration time) and mNG emission (acceptor, 505–545 nm, 50 ms integration time), and for the Halo-Nluc sensor, Nluc emission (donor, 445-470 nm, 50 ms integration time) and Halo618 emission (acceptor, 595–650 nm, 50 ms integration time) were measured at 37 °C (three measurements for baseline, 5–25 min with the agonist and/or compounds—see the figure legends for detailed description for respective experimental paradigms). The BRET ratios were defined as acceptor emission/donor emission. The *Z*-factor was calculated similarly to that in ref. ^80^ using 3 μM of 7α,25-dihydroxycholesterol-treated cells as a positive control and 0.1% DMSO-treated cells as a negative control. The measurements were performed using a Tecan Spark microplate reader.

### Generation of HEK293A cells stably overexpressing HiBiT-GPR183-(T233)-mNG-Nluc

HEK293A cells stably overexpressing HiBiT-GPR183-(T233)-mNG-Nluc were generated following transfection of ca. 4 × 10^5^ cells with 1000 ng of HiBiT-GPR183-(T233)-mNG-Nluc plasmid DNA construct. The cells were seeded onto a six-well plate (8 × 10^5^ cells) and cultured in DMEM (HyClone) supplemented with 10% foetal bovine serum. About 24 h after the transfection, the cells were passaged at 1:10, and 24 h later, the cell medium was supplemented with 1000 μg/mL G418 (Thermo Fisher Scientific). The medium was replaced every 2–3 days to select the cells transfected with the plasmid. The cells were maintained in the presence of the antibiotic for a period of 31 days until a stable culture was established. The stability of protein expression was verified by measuring Nluc luminescence. The stable cell lines were maintained in complete DMEM medium in the presence of 250 μg/mL G418. The cell line was used in the experiments to validate the sensor with GPR183 ligands and to determine its *Z*-factor.

### Bystander BRET to measure the cell surface expression of C-terminally HiBiT-tagged receptors

HEK293A cells at a density of 4 × 10^5^ cells/mL were transfected in suspension using PEI with 500 ng of the indicated receptor plasmid DNA and 500 ng of LgBiT-CAAX plasmid DNA. The cells in a volume of 100 µL were seeded onto a white 96-well plate with a flat bottom. 24 h later, the cells were washed once with 200 µL of HBSS (HyClone). Next, 90 μL of HBSS was added to the wells, and subsequently, 10 μL of furimazine (1:1000 final concentration, Promega) was added. The plate was incubated for 10 min and subsequently Nluc/NanoBiT luminescence (460–500 nm, 50 ms integration time) was measured using a Tecan Spark microplate reader.

### Live-cell ELISA to measure the cell surface expression of N-terminally HiBiT-tagged receptors

HEK293A cells at a density of 4 × 10^5^ cells/mL were transfected in suspension using PEI with 200 ng of the indicated receptor plasmid DNA and 800 ng ssDNA. The cells in a volume of 100 μL were seeded onto a transparent 96-well plate with a flat bottom. 24 h post-transfection, the cells were washed once with ice-cold 1.0% BSA in PBS and incubated with a mouse anti-HiBiT (1 µg/mL; Promega #N7200) in 1.0% BSA/PBS for 1 h at 4 °C. Following the incubation, the cells were washed twice with 1.0% BSA/PBS and incubated with a horseradish peroxidase-conjugated goat anti-mouse antibody (1:3000; Thermo Fisher Scientific #31430) in 1.0% BSA/PBS for 1 h at 4 °C. Subsequently, the cells were washed thrice with 1.0% BSA/PBS, and 50 µL of the peroxidase substrate TMB (3,3’,5,5’-tetramethylbenzidine; Sigma-Aldrich #T8665) was added. The cells were further incubated for 20 min, and upon development of a blue product, 50 µL of 1.2 M HCl was added, and the absorbance was read at 450 nm using a Tecan Spark microplate reader.

### Luminescence-based measurements of cell surface expression of N-terminally HiBiT-tagged receptors

HEK293A cells at a density of 4 × 10^5^ cells/mL were transfected in suspension using PEI with 200 ng of the indicated receptor plasmid DNA (HiBiT-GPR183 wild type (WT) or Y260F^6.51^) and 800 ng ssDNA. The cells in a volume of 100 μL were seeded onto a white 96-well plate with a flat bottom. 24 h post-transfection, the cells were washed once in HBSS and incubated with 100 μL of furimazine (1:100 final concentration, Promega) and LgBiT (1:200 final concentration, Promega) for 10 min. Subsequently, Nluc/NanoBiT luminescence (460–500 nm, 50 ms integration time) was measured using a Tecan Spark microplate reader.

### In vitro cell migration assay

In vitro cell migration was assessed using 96-well HTS transwell plates with an 8 μm pore size (Corning). Briefly, peripheral blood mononuclear cells (PBMC) from healthy donors (*n* = 6) (5 × 10^5^ cells/well) were stimulated with 10 μM or 100 nM 7α,25-dihydroxycholesterol in serum-free RPMI, as indicated in the figure legends. After 4 h incubation at 37 °C with 5% CO_2_, cells that had migrated to the lower chamber were harvested. They were first incubated with a viability dye for 10 min at 4 °C and then stained with fluorescently labelled antibodies (Supplementary Table 5) for 20 min at 4 °C. RANTES (Biotechne #278-RN; CCL5; 50 ng/mL) and unstimulated cells (US) were used as positive and negative controls, respectively. To inhibit GPR183-mediated migration, PBMC were pre-incubated with different concentrations of compound 78 or NIBR189 at 37 °C for 30 min. The cells were then transferred to the transwell plate and processed as mentioned above. Cells were acquired on a Cytek Aurora 3 and 5 L full-spectrum flow cytometer, and flow cytometry analysis was performed using FlowJo version 10.10.

### LC/MS analysis

Liver organotypic cell culture supernatants were thawed, and 30 μL of each sample was mixed with 30 µL methanol, followed by vortexing and centrifugation (5 min, 4 °C, 21,130 g). Quality control (QC) samples were prepared by pooling equal volumes of the supernatants from each sample. 40 µL of the supernatants were transferred into 2 mL glass vials containing 250 µL glass inserts with polymer feet (Agilent Technologies, Waldbronn, Germany). The vials were sealed with preslit polytetrafluoroethylene (PTFE)/silicone screw caps (Agilent Technologies, Waldbronn, Germany), and 1 μL was injected into the LC/MS system. Samples were analysed in randomized order using quadrupole time-of-flight mass spectrometry (QTOF-MS) after chromatographic separation on a Poroshell 120 EC-C18 column (2.7 μm, 2.1 × 100 mm; Agilent Technologies, Waldbronn, Germany). QC samples were analysed after every 10 samples and used to monitor the analytical precision, which was below 15% for all substances. LC/MS parameters were as described previously by us^81^, except that ionization was performed in positive ion mode and the mobile phases consisted of (A) water + 0.1% formic acid and (B) acetonitrile + 0.1% formic acid, and the scan range was *m*/*z* 50–1600. The system was operated using Mass Hunter Data Acquisition Software (version 10.1). Data preprocessing was carried out using Mass Hunter Profinder software (version B.08.00) via a targeted search for the sum formulas of NIBR189 (C21H21BrN2O3), 78 (C20H20BrN5O2), Midazolam (C18H13ClFN3), and OH-Midazolam (C18H13ClFN3O). Peak areas were exported as comma-separated value (CSV) files and visualized using GraphPad Prism (version 10.4.0). The mass spectrometry data can be found here: 10.6084/m9.figshare.30646547.

### Statistical analysis

All data presented in the main figures are based on at least three independent experiments (biological replicates), with each individual experiment typically performed at least in duplicates (technical replicates) for each condition, unless otherwise specified in a figure legend. One biological replicate refers to wells containing cells seeded from the same individual cell culture flasks and measured on the same day. Different biological replicates were transfected using separate transfection mixtures and measured on different days. Technical replicates are defined as individual wells with cells from the same biological replicate. Data presented in the Supporting Information, which indeed play a supporting role, may come from fewer than three biological experiments, and furthermore can be presented in the form of a representative example, therefore without a statistical analysis. Samples were not randomized or blinded during the experiments. Statistical and graphical analyses were performed using GraphPad Prism software (version 10.4.0). Concentration response data were fitted to either a three-parameter or a four-parameter non-linear model selected with the extra sum-of-squares *F*-test. Two datasets were analysed for statistical differences with paired *t*-test or Mann–Whitney test. Three or more datasets were analysed by one-way ANOVA with multiple comparison Dunnett’s post-hoc analysis or Kruskal–Wallis with multiple comparison Dunn’s post-hoc analysis. Significance levels are given and displayed in the figures as: *P  <  0.05; **P  <  0.01; ***P  <  0.001; ****P  <  0.0001. Differences between datasets that did not reach statistical significance are left unmarked or marked with “ns”. Data points throughout the manuscript are indicated as the mean ± standard error of the mean (SEM) unless otherwise stated.

### Reporting summary

Further information on research design is available in the Nature Portfolio Reporting Summary linked to this article.

## Supplementary information

Supplementary Information

Description of Additional Supplementary Files

Supplementary Data 1

Supplementary Data 2

Reporting Summary

Transparent Peer Review File

## Source data

Source Data

## Data Availability

Source data are provided as a Source Data file. The active and inactive GPR183 structures used in this study are available from the Protein Data Bank under 7TUZ and 7TUY. Mass spectrometry data are deposited on Figshare (identifier: 10.6084/m9.figshare.30646547). MD data are publicly available via Zenodo at 10.5281/zenodo.20038630. Source data are provided with this paper.

## Code availability

The virtual screening analyses in this study were performed using PyRMD2Dock, originally developed by some of us and described previously (10.1021/acs.jcim.3c00647). The source code is publicly available at https://github.com/cosconatilab/PyRMD as an open-source tool.
