# Supplementary material for: Structure-based screening and a conformational biosensor identify a GPR183 inverse agonist and an activation switch
Source: Nat Commun. 2026 May 30;17:7020. doi: 10.1038/s41467-026-73857-9 (PMC13392048; doi:10.1038/s41467-026-73857-9)
Supplement: Supplementary file 1 — Supplementary Information [file 41467_2026_73857_MOESM1_ESM.pdf]

Structure-based screening and a conformational biosensor identify a GPR183 inverse agonist and an activation switch

Supplementary Information

Louise Andersson [1#], Michele Roggia [2#], Kittikorn Wangriatisak [3,4], Rhiannon Skye Kozel [1], Holly R. Brittain [1], Sonia Youhanna [5], Maria Gil [3,4], Mathias Haag [6,7], Volker M. Lauschke [5,6,7], Karine Chemin [3,4], Sandro Cosconati [2*], Paweł Kozielewicz [1*]

Affiliations

[1] Molecular Pharmacology of GPCRs, Department of Physiology and Pharmacology, Karolinska Institutet, 171 65 Solna, Stockholm, Sweden

[2] DiSTABiF, University of Campania Luigi Vanvitelli, Via Vivaldi, 43, 81100 Caserta, Italy

[3] Division of Rheumatology, Department of Medicine, Solna, Karolinska Institutet, Karolinska University Hospital, 171 76, Solna, Stockholm, Sweden

[4] Center for Molecular Medicine, Karolinska Institutet, 171 76 Solna, Stockholm, Sweden

[5] Personalized Medicine and Drug Development, Department of Physiology and Pharmacology, 171 65, Solna, Stockholm, Sweden

[6] Dr Margarete Fischer-Bosch Institute of Clinical Pharmacology, 70376 Stuttgart, Germany

[7] University of Tübingen, 72074 Tübingen, Germany

[#] - equal contribution

[*] To whom correspondence should be addressed:

Pawel Kozielewicz – pawel.kozielewicz@ki.se

Sandro Cosconati - sandro.cosconati@unicampania.it

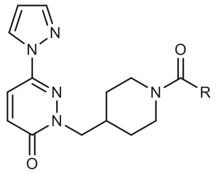

ID	R	ΔebBRET ± SEM (%)	ID	R	ΔebBRET ± SEM (%)
**43**	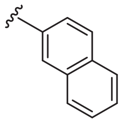	-28476 ± 2218	**88**	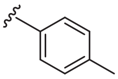	-19435 ± 9915
**71**	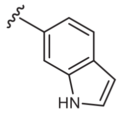	-8949 ± 9602	**89**	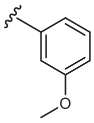	-7404 ± 4478
**72**	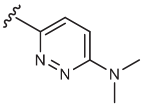	-14883 ± 11911	**90**	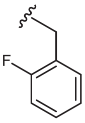	-4102 ± 9248
**73**	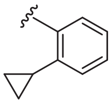	-8149 ± 8278	**91**	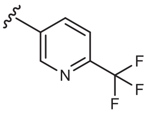	-12357 ± 1916
**74**	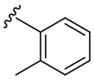	-1497 ± 6662	**92**	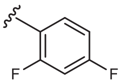	-10567 ± 2899
**75**	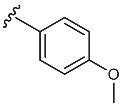	-18827 ± 6881	**93**	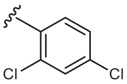	-14975 ± 4035
**76**	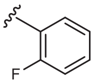	-5744 ± 6372	**94**	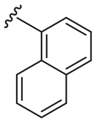	-4422 ± 6517
**77**	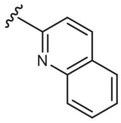	1181 ± 10043	**95**	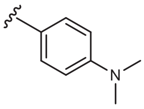	-13929 ± 6537
**78**	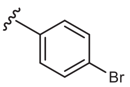	**-35503 ± 5629**	**96**	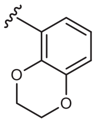	-14164 ± 8583
**79**	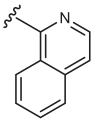	650 ± 14832	**97**	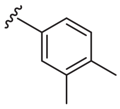	-17020 ± 4582

**80**	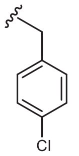	3264 ± 13352	**98**	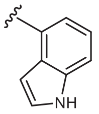	969 ± 6546
**81**	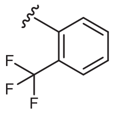	-6734 ± 4842	**99**	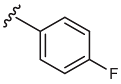	-6398 ± 4005
**82**	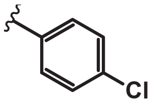	-27065 ± 8373	**100**	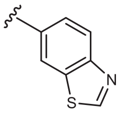	-18018 ± 7267
**83**	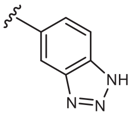	-15270 ± 2562	**101**	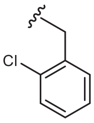	10563 ± 3549
**84**	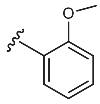	-2921 ± 7809	**102**	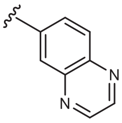	-3513 ± 3341
**85**	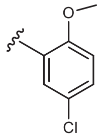	-4578 ± 1768	**103**	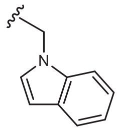	-19090 ± 4321
**86**	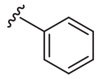	-10167 ±3939	**104**	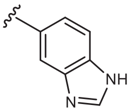	-14289 ± 5544
**87**	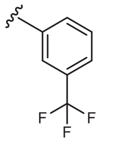	-11432 ± 7356			

Supplementary Table 1. Structures of the 34 analogues of **43,** along with their experimental ΔebBRET (%).

	**78**	**GSK682753A**	**NIBR189**	**Range 95% of Drugs**	**Description**
Molecular Weight	442.314	479.789	429.312	130.0 / 725.0	
No. of Rotatable Bonds	3	5	5	0.0/15.0	
QP log P for octanol/water	4.222	5.489	4.108	-2/6.5	Predicted octanol/water partition coefficient.
QP log S for aqueous solubility	-5.733	-7.437	-5.564	-6.5/0.5	Predicted aqueous solubility, log S. S in mol dm^{-3} is the concentration of the solute in a saturated solution that is in equilibrium with the crystalline solid.
QP log K hsa Serum Protein Binding	0.415	0.661	0.173	-1.5/1.5	Prediction of binding to human serum albumin.
QP log BB for brain/blood	-0.524	-0.341	-0.449	-3.0/1.2	Predicted brain/blood partition coefficient.
No. of Primary Metabolites	0	1	1	1.0/8.0	Number of likely metabolic reactions
Apparent Caco-2 Permeability (nm/sec)	909	1121	1584	<25 poor, >500 great	Predicted apparent Caco-2 cell permeability in nm/sec. Caco-2 cells are a model for the gut-blood barrier.
Apparent MDCK Permeability (nm/sec)	1188M	7088M	2159	<25 poor, >500 great	Predicted apparent MDCK cell permeability in nm/sec. MDCK cells are considered to be a good mimic for the blood-brain barrier.
Lipinski Rule of 5 Violations	0	1	0	maximum is 4	
% Human Oral Absorption in GI (+-20%)	100	100	100	<25% is poor	Predicted human oral absorption on 0 to 100% scale.

Supplementary Table 2. Qikprop properties and description calculated for 78, GSK682753A, and NIBR189**.**

Parameter	Value/Description
**Initial structure**	78-GPR183 complex
**Software**	Amber24 (PMEMD.cuda)
**Simulation box type**	Orthorhombic
**Simulation box volume**	70.2 x 70.2 x 117.7 Å
**Solvent model**	TIP3P
**Salt concentration**	0.15 M KCl
**Neutralization**	K^+/Cl^- ions added to neutralize charge
**Total number of atoms**	53,614
**Total number of water molecules**	11,248
**Force field**	ff19SB (protein), gaff2 (ligand), lipid21 (membrane)
**Simulation time**	1.5 μs per replica
**Recording interval**	50000 steps
**Ensemble class**	NPγT ensemble
**Temperature**	300 K
**Pressure**	1 bar
**Lipid composition**	POPC (1-palmitoyl-2-oleoylphosphatidylcholine)

Supplementary Table 3. Molecular dynamics simulation system setup.

Primer	Sequence (5'-3')
to generate HiBiT-GPR183 construct	
HiBiT_GPR183_backbone_fwd	TCCAGTAATGGGAAGTAATCTAGAGGGCCC
HiBiT-GPR183_backbone_rev	CATCTGGATGTCCATGGATCCGCTAATCTTCTT
HiBiT-GPR183_fwd	AAGAAGATTAGCGGATCCATGGACATCCAGATG
HiBiT_GPR183_rev	GGGCCCTCTAGATTACTTCCCATTACTGGA
to generate HiBiT-GPR183-Nluc construct	
GPR183_Nluc_insert_fwd	AAGAAGATTAGCGGATCCATGGACATCCAGATG
GPR183_Nluc_insert_rev	TTCGAGTGTGAAGACCTTCCCATTACTGGA
GPR183_Nluc_backbone_fwd	TCCAGTAATGGGAAGGTCTTCACACTCGAA
GPR183_Nluc_backbone_rev	CATCTGGATGTCCATGGATCCGCTAATCTTCTT
to generate HiBiT-GPR183-mNG-Nluc constructs	
GPR183_mNG231_ins_fwd	GCCAAGCAAAACCCCGTGAGCAAGGGCGAG
GPR183_mNG231_ins_rv	GCTTTTTCGGTAAGCTTGTACAGCTCGTC
GPR183_mNG231_bb_fwd	GACGAGCTGTACAAGCTTACCGAAAAAGC
GPR183_mNG231_bb_rv	CTCGCCCTTGCTCACGGGGTTTTGCTTGGC
GPR183_mNG233_ins_fwd	CAAAACCCCCTTACCGTGAGCAAGGGCGAG
GPR183_mNG233_ins_rv	GACGCCGCTTTTTCCTTGTACAGCTCGTC
GPR183_mNG233_bb_fwd	GACGAGCTGTACAAGGAAAAAGCGGCGTC
GPR183_mNG233_bb_rv	CTCGCCCTTGCTCACGGTAAGGGGGTTTTG
GPR183_mNG235_ins_fwd	CCCCTTACCGAAAAAGTGAGCAAGGGCGAG
GPR183_mNG235_ins_rv	CTTGTTGACGCCGCTCTTGTACAGCTCGTC
GPR183_mNG235_bb_fwd	GACGAGCTGTACAAGAGCGGCGTCAACAAG
GPR183_mNG235_bb_rv	CTCGCCCTTGCTCACTTTTTCGGTAAGGGG

to *generate HA-GPR183-HiBiT construct*	
GPR183_HiBiT_insert_fwd	GATTATGCGGGATCCATGGACATCCAGATG
GPR183_HiBiT_insert_rev	CCGCCAGCCGCTCACCTTCCCATTACTGGA
GPR183_HiBiT_backbone_fwd	TCCAGTAATGGGAAGGTGAGCGGCTGGCGG
GPR183_HiBiT_backbone_rev	CATCTGGATGTCCATGGATCCCGCATAATC
to generate HA-GPR183-(T233)-mNG2$_{(11)}$-HiBiT construct	
GPR183_mNG11_ins_fwd	CAAAACCCC CTTACCACCGAGCTCAACTTC
GPR183_mNG11_ins_rv	GACGCCGCTTTTTCCATCATATCGGTAAA
GPR183_mNG11_bb_fwd	TTTACCGATATGATG GAAAAAGCGGCGTC
GPR183_mNG11_bb_rv	GAAGTTGAGCTCGGTGGTAAGGGGGTTTTG
to generate HiBiT-GPR183-Halo-Nluc construct	
GPR183_Halo_ins_fwd	CAAAACCCCCTTACCGAAATCGGTACTGGC
GPR183_Halo_ins_rv	GACGCCGCTTTTTCACCGGAAATCTCCAG
GPR183_Halo_bb_fwd	CTGGAGATTTCCGGTGAAAAAGCGGCGTC
GPR183_Halo_bb_rv	GCCAGTACCGATTTCGGTAAGGGGGTTTTG
to generate HiBiT-β$_2$AR construct	
HiBiT_B2AR_backbone_fwd	AATGACTCACTGCTGTAATCTAGAGGGCCC
HiBiT-B2AR_backbone_rev	GCCGTTCCCGGGTTGCCCGGATCCGCTAATCTTCTT
HiBiT-B2AR_fwd	AAGAAGATTAGCGGATCCGGGCAACCCGGGAACGGC
HiBiT_B2AR_rev	GGGCCCTCTAGATTACAGCAGTGAGTCATT
to generate mutant constructs	
GPR183_C201A_fwd	ATACTGCTGGGGGCCGCTTTCATTGGGTACGT

GPR183_C201A_rev	ACGTACCCAATGAAAGCGGCCCCCAGCAGTAT
GPR183_Y260F_fwd	TGTGCTTCACTCCCTTTCATGTCGCCATCAT
GPR183_Y260F_rev	ATGATGGCGACATGAAAGGGAGTGAAGCACA
GPR183_H261A_fwd	TGCTTCACTCCCTATGCTGTCGCCATCATTCA
GPR183_H261A_rev	TGAATGATGGCGACAGCATAGGGAGTGAAGCA

Supplementary Table 4. Primer DNA sequences.

Antibody	Fluorophore	Clone	Company	Dilution	Catalogue No.
**Fixable viability dye**	Green	-	Biolegend	1:2000	423107
**CD3**	PE/Cy5.5	7D6	ThermoFisher	1:100	MHCD0318
**CD4**	BV786	SK3	BD	1:100	563877
**CD8**	BUV395	RPA-T8	BD	1:100	563795
**CD14**	FITC	M5E2	BD	1:100	555397
**CD16**	APC/H7	3G8	BD	1:200	560195
**CD19**	BV421	HIB19	Biolegend	1:50	302234
**CD56**	PE/Cy5	B159	BD	1:100	555517
**GPR183**	APC	SA313E4	Biolegend	1:50	368908

Supplementary Table 5. Antibodies used for chemotaxis assay.

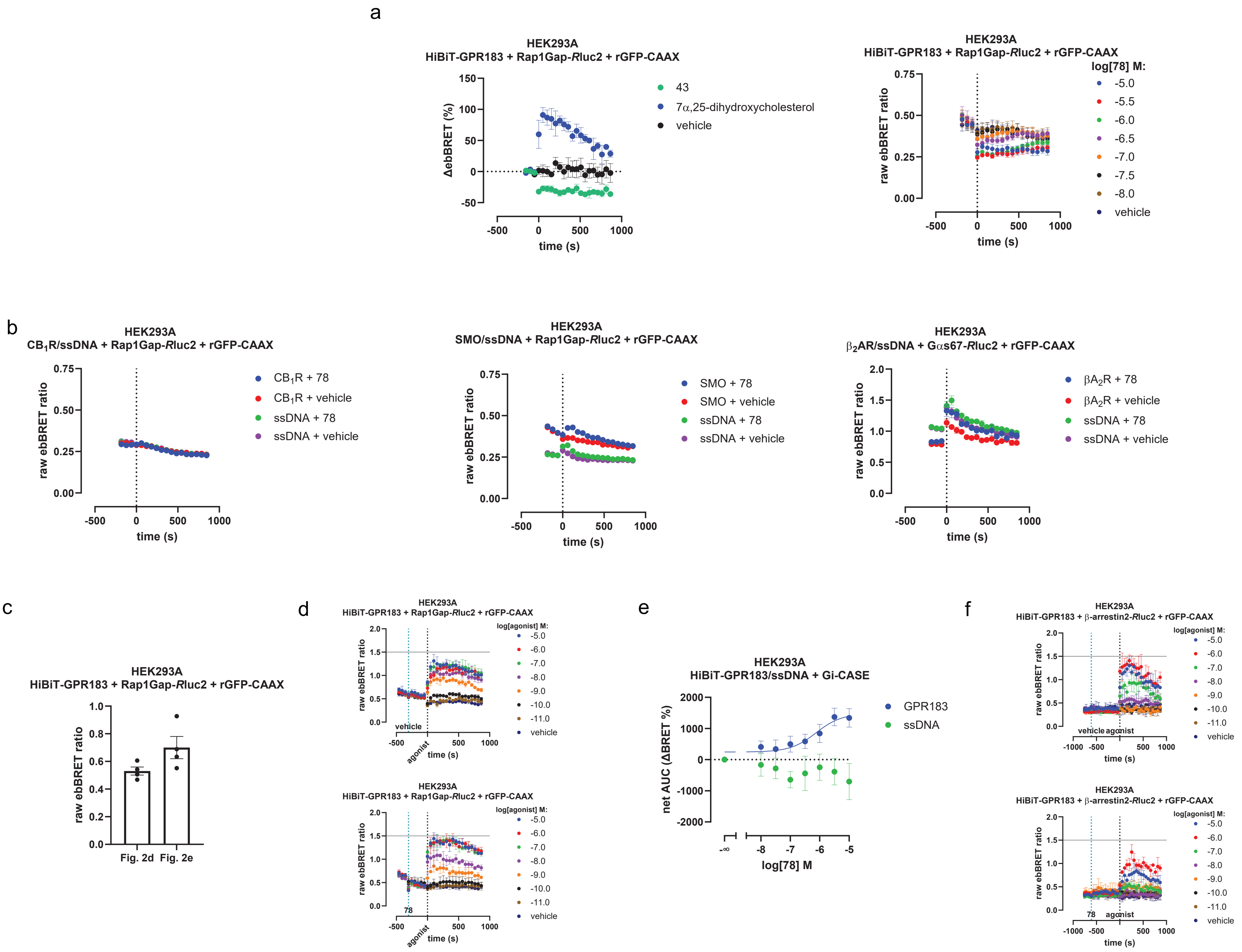

Supplementary Figure 1

Supplementary Figure 1. Assay validation and selectivity profiling. a Left: The kinetic plot from a representative ebBRET-based Gi activation assay. The plate was measured three times to obtain baseline BRET, and then the compounds or the vehicle (0.1% DMSO) were added, and the plate was measured for another 15 min. The data are presented as ΔebBRET % over the three baseline reads measured prior to the compound/vehicle addition; vehicle (0.1% DMSO) was subtracted. The data represent mean ± SEM from three biological replicates. **Right:** The same as in **a** but presented as raw ebBRET for **78**; vehicle (1.0% DMSO) was not subtracted. The data represent mean ± SEM from five biological replicates. **b** The kinetic plot from ebBRET-based Gi activation assays to assess selectivity of compound **78** on different GPCRs. The data represent raw ebBRET ratio and come from three biological replicates and are presented as mean ± SEM. **c** Basal raw ebBRET ratios indicative of constitutive activity of an overexpressed HiBiT-GPR183 from the experiments from the **Fig. 2d** and **2e**. The data represent mean ± SEM from four biological replicates. **d** The kinetic plots from a representative 10 μM 78 competition ebBRET-based Gi activation assay. The plate was measured three times to obtain baseline BRET, and then, as indicated with the left vertical coloured dashed line, the vehicle (1.0% DMSO; **Top**) or 10 μM of 78 (**Bottom**) were added, the plate was measured for another 6 min. Next, as indicated with the right vertical black dashed line, different concentrations of 7α,25-dihydroxycholesterol were added. The plate was measured for 15 min. The horizontal dashed line at ebBRET ratio=1.5 is inserted for easier comparison of maximal signals. The data are presented as raw ebBRET ratio. The data (mean ± SD) come from one representative experiment performed in three technical replicates. **e** Compound **78** induces a concentration-dependent increase in BRET in the Gi-CASE setup indicative of a decrease GPR183-mediated Gi activation. The data are presented as net AUC ΔBRET % over the three baseline reads measured prior to the compound addition; vehicle (1.0% DMSO) was subtracted. The measurement lasted 15 min following the addition of **78**. The data come from three to four

biological replicates and are presented as mean ± SEM. **f** The kinetic plots from a representative 10 μM 78 competition ebBRET-based β-arrestin2 recruitment assay. The plate was measured three times to obtain baseline BRET, and then, as indicated with the left vertical coloured dashed line, the vehicle (1.0% DMSO; **Top**) or 10 μM of 78 (**Bottom**) were added, the plate was measured for another 10 min. Next, as indicated with the right vertical black dashed line, different concentrations of 7α,25-dihydroxycholesterol were added. The plate was measured for 15 min. The horizontal dashed line at ebBRET ratio=1.5 is inserted for easier comparison of maximal signals. The data are presented as raw ebBRET ratio. The data (mean ± SD) come from one representative experiment performed in three technical replicates. Source data are provided as a Source Data file.

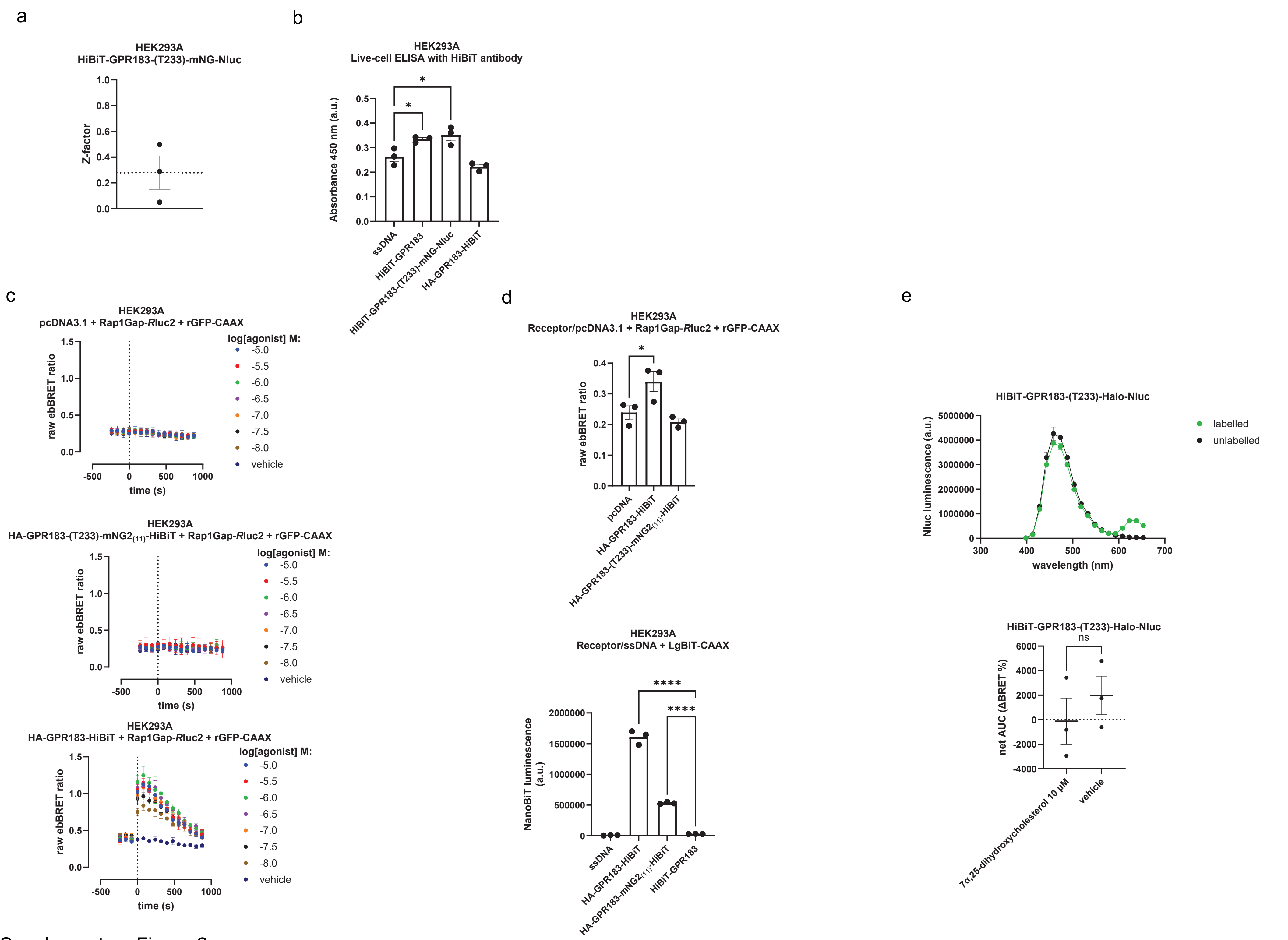

Supplementary Figure 2

Supplementary Figure 2. **Validation of the HiBiT-GPR183-(T233)-mNG-Nluc sensor. a** Z-factor evaluation of the HiBiT-GPR183-(T233)-mNG-Nluc sensor using the HEK293A cells stably overexpressing the construct. The data are presented as the mean ΔBRET% (over the three initial baseline reads) from the 15 minute-long read following the addition of the agonist or vehicle (0.1% DMSO) for three separate plates. **b** HiBiT-GPR183-(T233)-mNG-Nluc is expressed at the cell surface upon overexpression in HEK293A cells. HiBiT-GPR183 was used as a positive control, while ssDNA- and HA-GPR183-HiBiT-transfected cells were used as negative controls. The data represent mean ± SEM of three biological replicates and were analysed for differences using one-way ANOVA with multiple comparison Dunnett's post-hoc analysis; * $P<0.05$. a.u. – arbitrary units. **c Left top**: pcDNA.3.1-transfected cell nor (**left middle**) HA-GPR183-(T233)-mNG2$_{(11)}$-transfected cells do not respond to agonist stimulation to activate Gi in a GPR183-specific manner, as opposed to (**left bottom**) HA-GPR183-HiBiT, which does not have an ICL3-inserted tag. 0.1% DMSO was used as a vehicle. The data come from a representative experiment of three biological replicates and are shown as mean ± SD of three technical replicates. **d Top**: Ligand-unbound HA-GPR183-(T233)-mNG2$_{(11)}$ does not constitutively activate Gi. **Bottom**: HA-GPR183-(T233)-mNG2$_{(11)}$ is trafficked to the cell membrane. The data are shown as mean ± SEM of three biological replicates. The data were analysed for differences using one-way ANOVA with multiple comparison Dunnett's post-hoc analysis; * $P<0.05$, ****$P < 0.0001$. **e Top**: Upon the overexpression of the HiBiT-GPR183-(T233)-Halo-Nluc conformational sensor and addition of the Halo substrate, there is an energy transfer between the Nluc and Halo (detectable a luminescence peak over 600 nm). **Bottom**: The addition of 10 μM of the agonist does not result in a notable change in the BRET signal in comparison with the vehicle (0.1% DMSO). The mean values, which come from three biological replicates, were compared using the Mann-Whitney test. Source data are provided as a Source Data file.

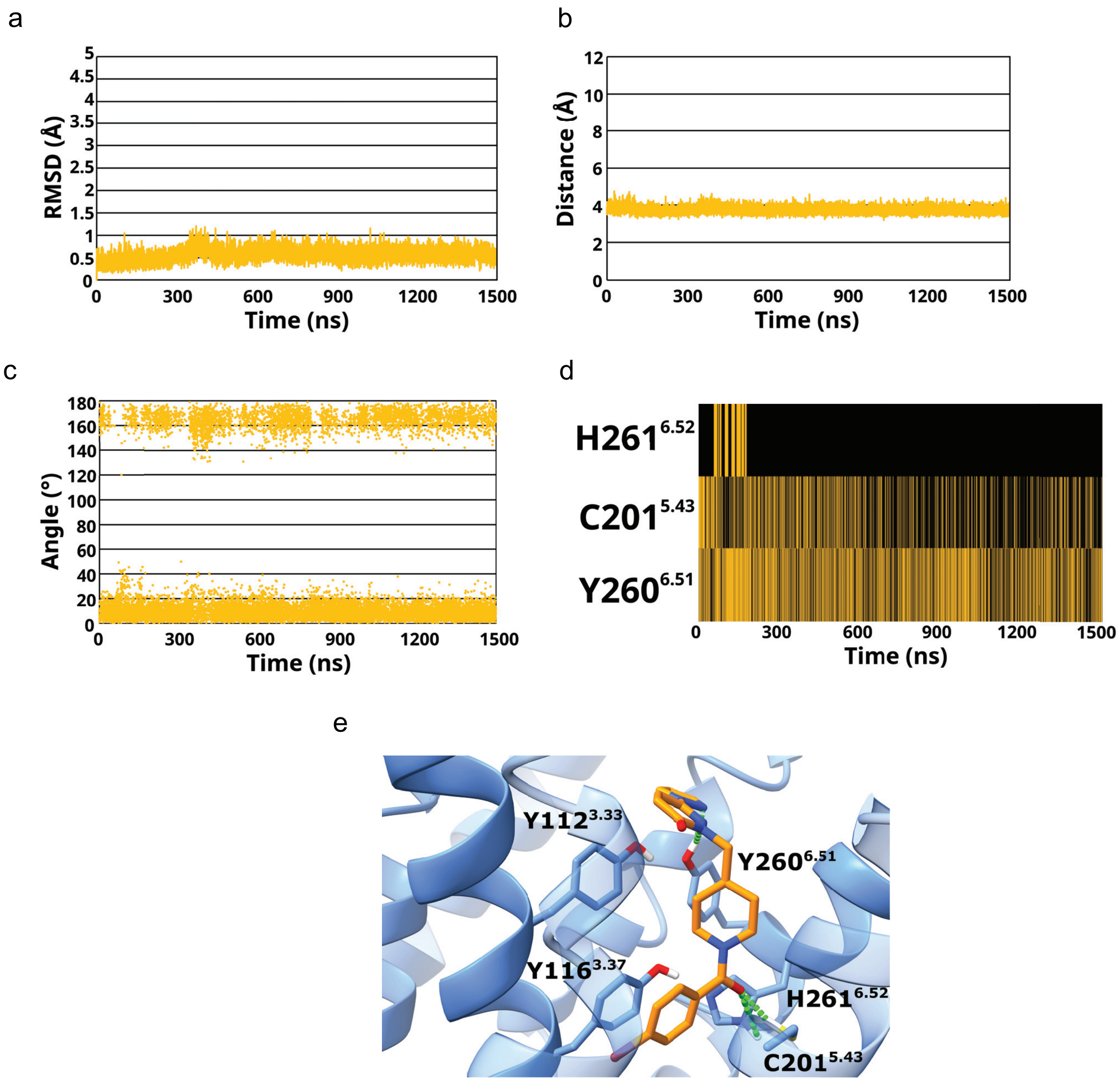

Supplementary Figure 3

Supplementary Figure 3. **Analysis of 78 binding to the active-state GPR183.** Analysis of **a** RMSD, **b** distance, and **c** angles plots over time (ns) of compound **78** in complex with the active state of GPR183. **d** Hydrogen bond interactions vs trajectory time between **78** and C201$^{5.43}$, H261$^{6.52}$, and Y260$^{6.51}$. **e** Representative structure of the single, well-defined cluster of **78** in complex with the GPR183 protein in its active state. The ligand is depicted as orange sticks while the protein is shown as blue sticks and ribbons. Hydrogen bonds are represented as green dashes. Source data are provided as a Source Data file.

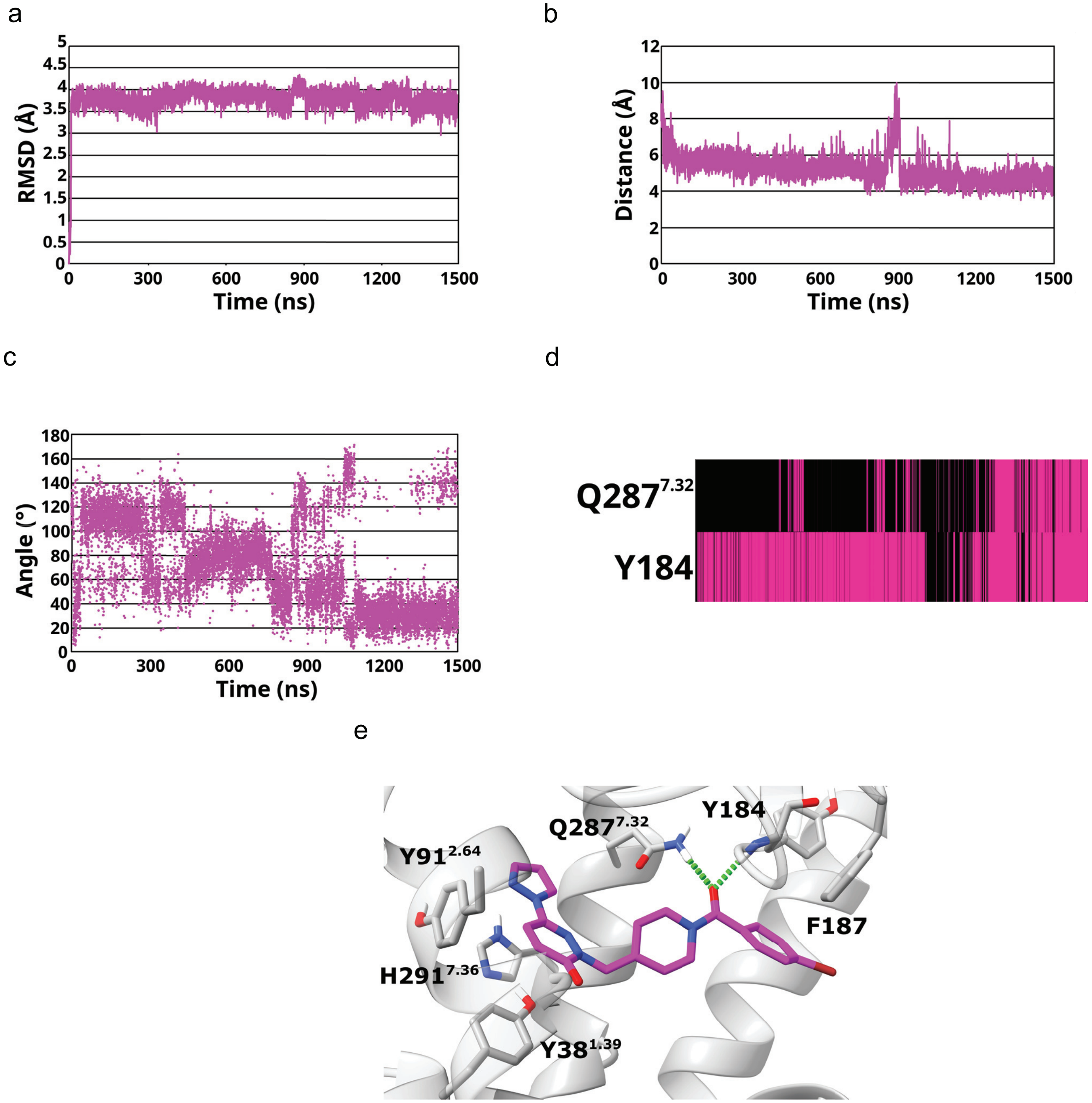

Supplementary Figure 4

Supplementary Figure 4. Analysis of 78 binding to the inactive-state GPR183. Analysis of **a** RMSD, **b** distance, and **c** angles throughout the trajectory (in ns) for compound **78** bound to the inactive state of GPR183. **d** Hydrogen bonds formed between **78** and Q287$^{7.32}$ and Y184 shown versus simulation time. **e** Representative conformer from the most populated cluster of **78** complexed with the GPR183 protein (inactive state). The ligand is rendered as magenta sticks; the protein is visualized as grey sticks and ribbons. Hydrogen bonds are highlighted as green dashed lines. Source data are provided as a Source Data file.

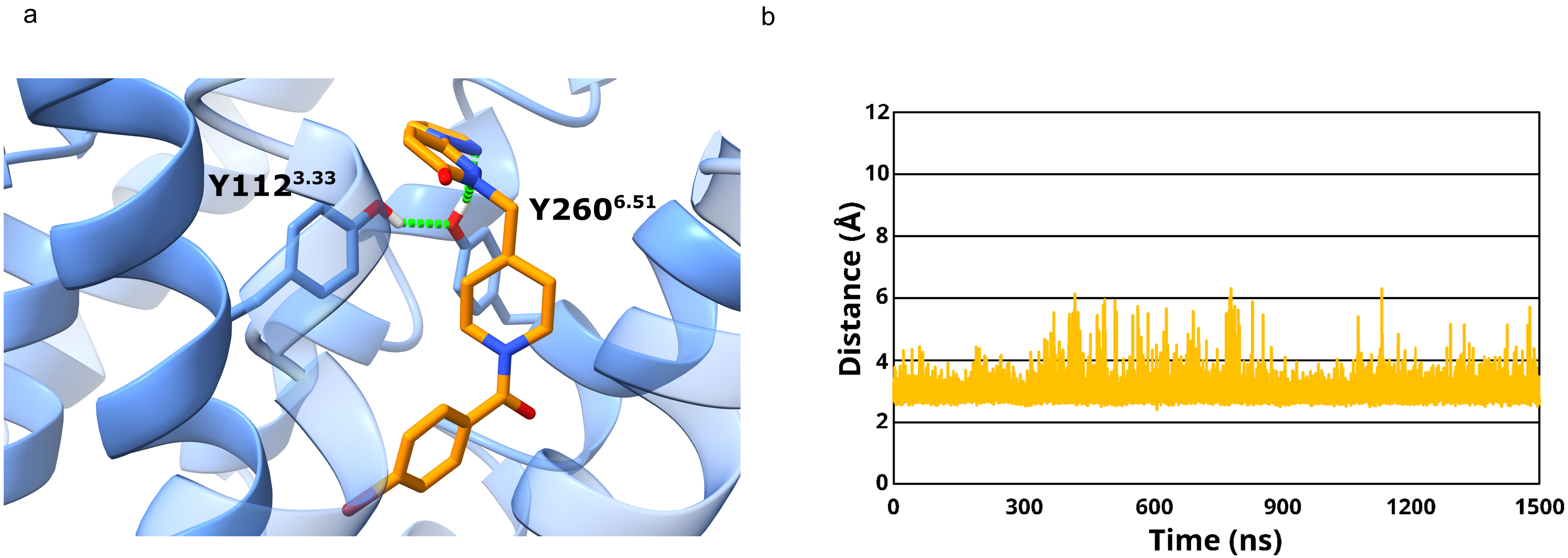

Supplementary Figure 5

Supplementary Figure 5. Analysis of the hydrogen bond network between 78's pyrazole ring in the active-state GPR183. a Representative structure of the single, well-defined cluster of **78** in complex with the GPR183 protein in its active state. The ligand is depicted as orange sticks, while the protein is shown as blue sticks and ribbons. Hydrogen bonds are represented as green dashes. **b** distance between $Y112^{3.33}$ and $Y260^{6.51}$ OH groups throughout the trajectory (in ns). Source data are provided as a Source Data file.

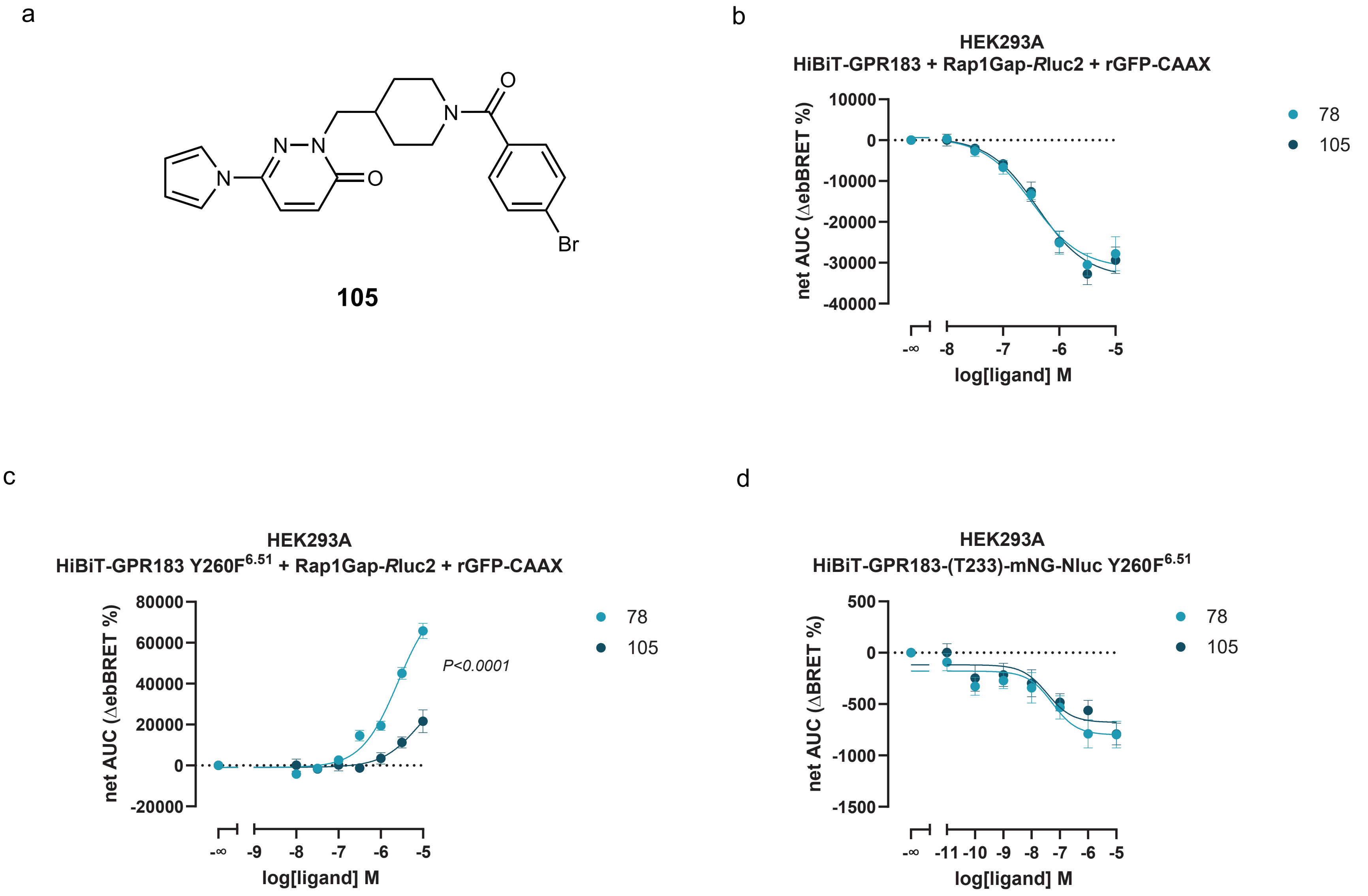

Supplementary Figure 6

Supplementary Figure 6. Pharmacological profiling of compound 105. a Chemical structural formula of **105** (ChemSketch 2021 2.0)**. b 105** induces a concentration-dependent decrease in ebBRET indicative of blockage of GPR183-mediated Gi activation to the same degree as **78**. The data are shown as mean ± SEM of four biological replicates for **78** and five biological replicates for 105 **c 105** induces a concentration-dependent increase in ebBRET indicative of GPR183 Y260F$^{6.51}$-mediated activation of Gi but with a lower potency and efficacy compared with **78;** Differences in $logEC_{50}$ values and in the top or bottom plateaus of the curves were analysed using an F-test; data for **78** are also shown in **Fig. 7b.** The data are shown as mean ± SEM of four biological replicates **d 105** induces a concentration-dependent decrease in BRET using a mNG-Nluc biosensor indicative of a conformational change in the receptor to the same degree as **78**. The data are presented as net AUC of ΔBRET % (over three baseline measurements), vehicle (1.0% DMSO) was subtracted). The data for **78** are also shown in the **Fig. 7f**. The data are shown as mean ± SEM of seven biological replicates. Source data are provided as a Source Data file.

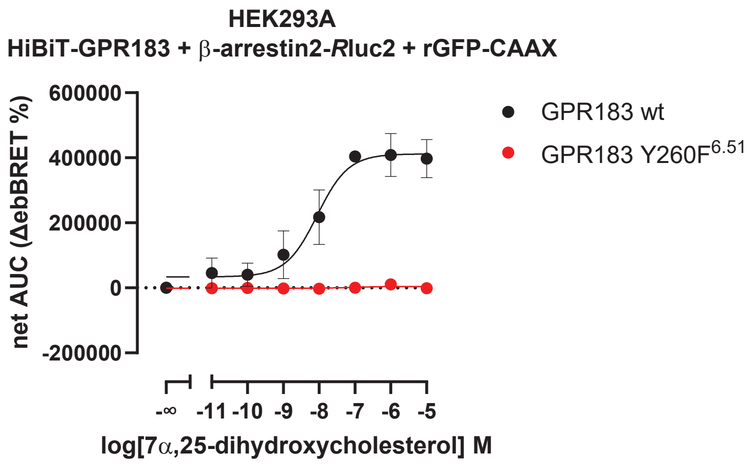

Supplementary Figure 7

Supplementary Figure 7. Y260F$^{6.51}$ mutation abrogates agonist-mediated β-arrestin2 recruitment. 7α,25-dihydroxycholesterol induces a concentration-dependent increase in BRET in the β-arrestin2-*R*luc2 with rGFP-CAAX setup in the presence of the overexpressed GPR183 WT but not the Y260F$^{6.51}$. The data are presented as net AUC ΔebBRET % over the three baseline reads measured prior to the compound addition; 15 minute-long stimulation; vehicle (1.0% DMSO) was subtracted. The data come from three biological replicates and are presented as mean ± SEM. Source data are provided as a Source Data file.

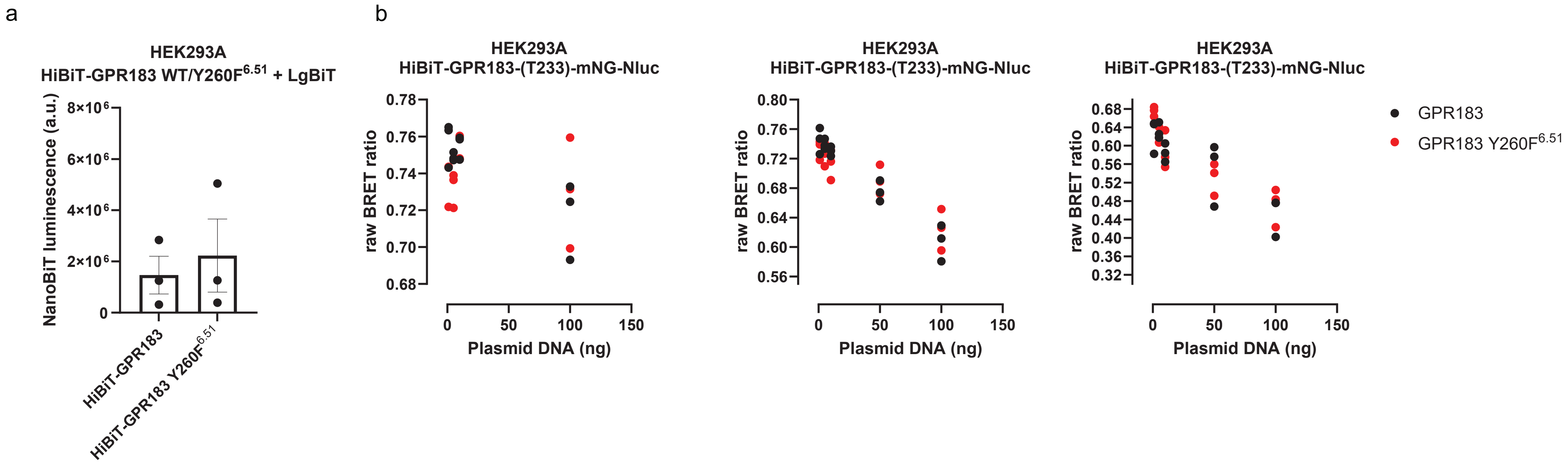

Supplementary Figure 8

Supplementary Figure 8. GPR183 WT and Y260F$^{6.51}$ present themselves with the same levels of the cell surface expression and basal conformational state. a Cell surface expression of the two variants was measured using a HiBiT-LgBiT complementation assay on living cells. The data are presented as mean ± SEM from three biological replicates; a.u. – arbitrary units. **b** Basal raw BRET ratios measured upon overexpressing different amounts of GPR183-(T233)-mNG-Nluc WT and Y260F$^{6.51}$ are alike indicating that the basal, i.e. in the absence of an exogenous ligand, conformation of the two variants are likely to be very similar. Data from all individual wells from three biological replicates are shown. Data in the **b left** and **middle** come from the experiments performed using a black 96-well plate, data in the **b right** come from the experiment performed on a white 96-well plate. Source data are provided as a Source Data file.

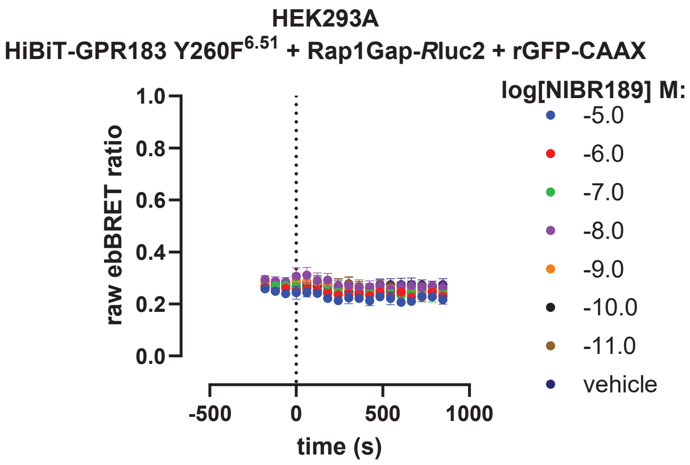

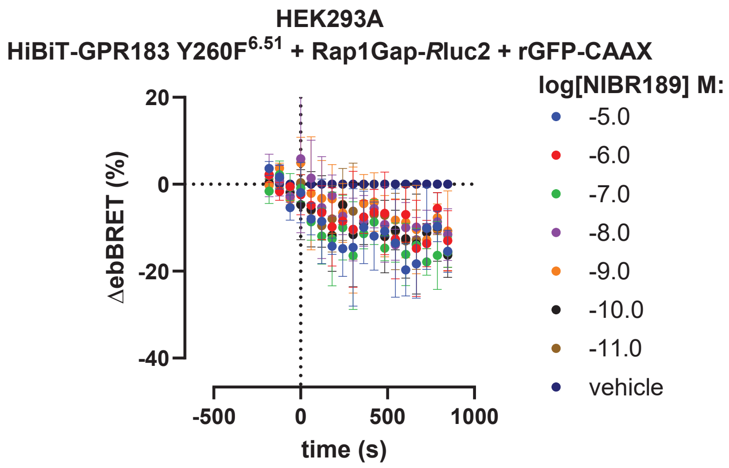

Supplementary Figure 9

Supplementary Figure 9. NIBR189 behaves as an inverse agonist on a GPR183 Y260F$^{6.51}$. NIBR189 induces a subtle concentration-dependent reduction in BRET between Rap1Gap-*R*luc2 and rGFP-CAAX in the presence of an overexpressed GPR183 Y260F$^{6.51}$ as presented with the raw values (l**eft**) as well as baseline and vehicle-subtracted (**right**). The data come from three biological replicates and are presented as mean ± SEM. Source data are provided as a Source Data file.

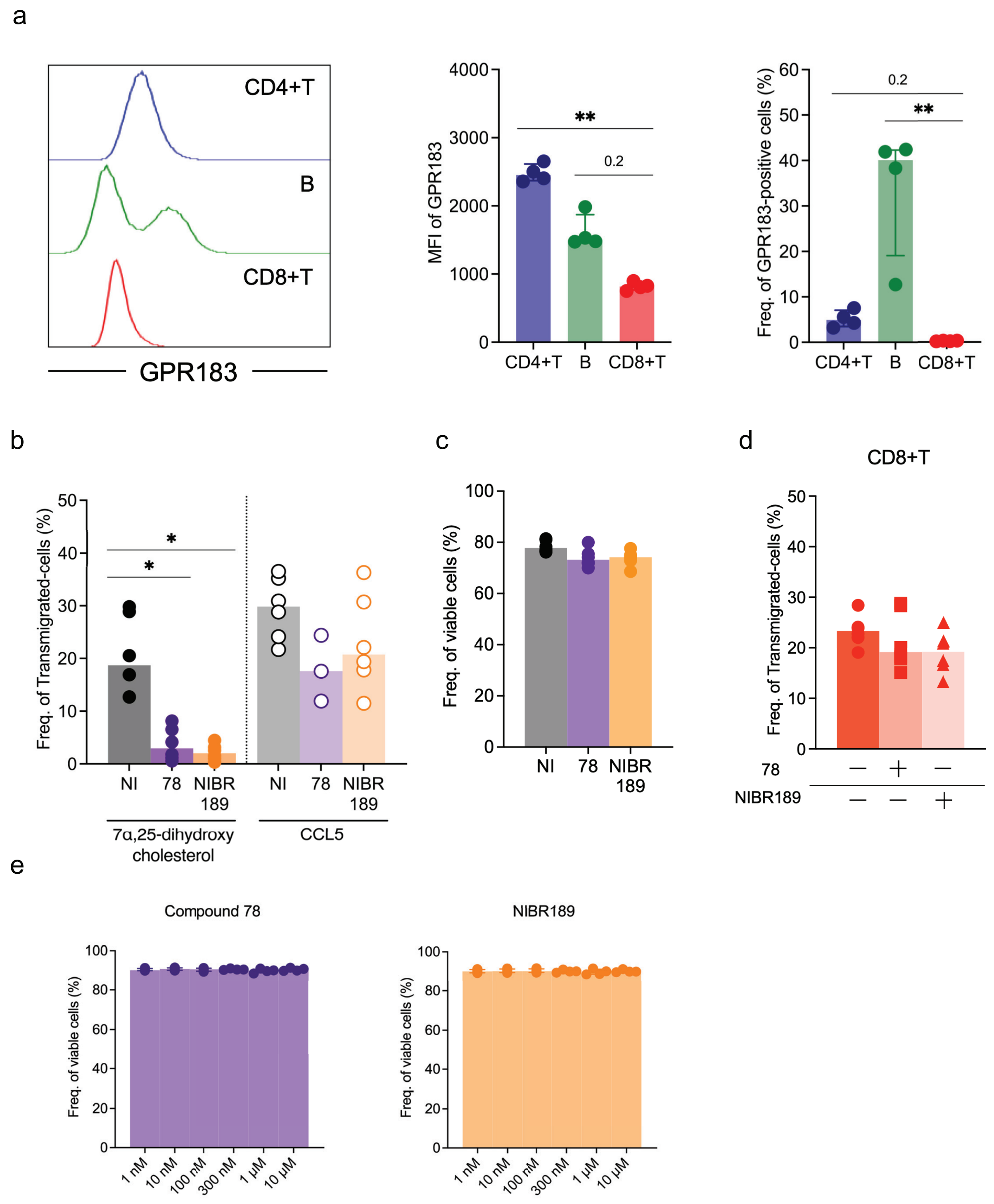

Supplementary Figure 10

Supplementary Figure 10. Supplementary data from migration assays. a Histogram overlays representing GPR183 expression (**left**), median fluorescent intensity (MFI) of GPR183 (**middle**), and frequency of GPR183-positive cells (**right**) of GPR183 in CD4+T, B and CD8+T cells in healthy control (HC, n=4). **b** Frequency of migratory lymphocytes treated with **78**, NIBR189, or no-inhibitor (NI) after stimulation in HC (n=6). **c** Frequency of viable cells treated with **78**, NIBR189, or NI following 7α,25-dihydroxycholesterol stimulation (n=6). **d** Frequency of transmigrated CD8+T cells after addition of **78** and NIBR189 to 7α,25-dihydroxycholesterol-stimulated (0.1% DMSO final concentration) samples from HC (n=6). **e** Different concentrations of **78** or NIBR189 do not induce PBMCs death (n=4). Data come from four to six biological replicates and are presented as mean ± SEM (**b-d**) 7α,25-dihydroxycholesterol = 10 μM. Source data are provided as a Source Data file.

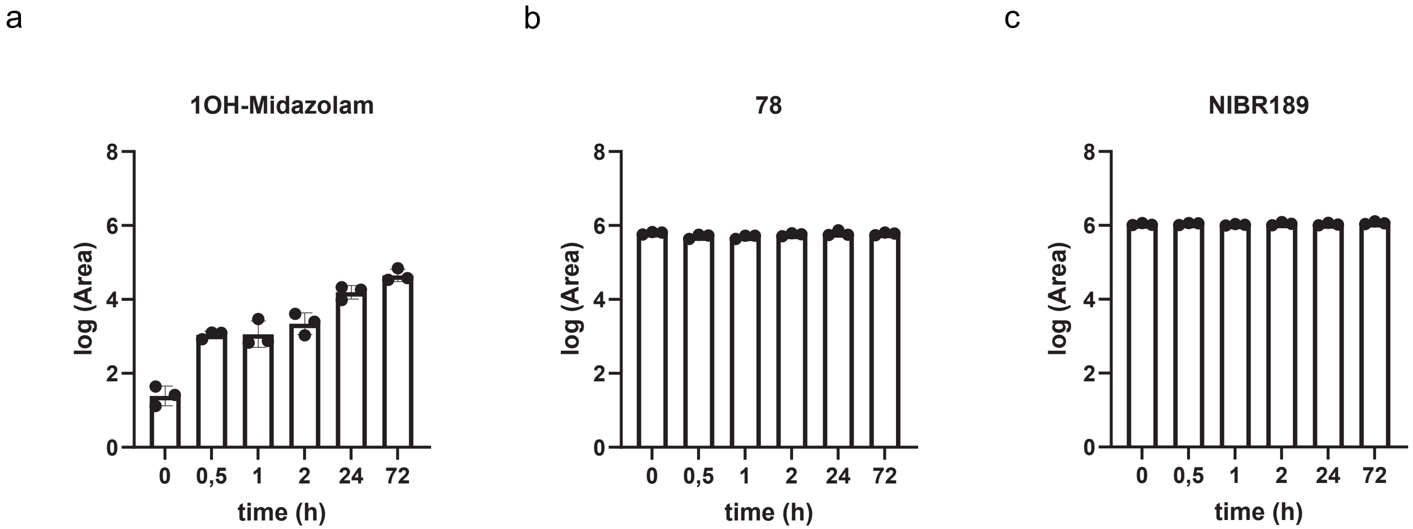

Supplementary Figure 11

Supplementary Figure 11. ***In vitro* hepatic clearance assessments**. **a** Upon incubation with the CYP3A4 substrate midazolam, liver spheroids showed significant time-dependent increases in the metabolic product 1OH-midazolam, confirming metabolic activity of the culture throughout the incubation period. **b** Hepatic clearance of **78** in liver spheroids upon incubation with 1 μM of test article. **c** Hepatic clearance of NIBR189 in liver spheroids upon incubation with 1 μM of test article. The results demonstrate stable concentrations, i.e., the absence of considerable hepatic metabolism. Ordinate values represent the log-transformed areas under the mass spectrometry peaks. For 1OH-midazolam: 341.0732 Da at 3.2 min; for compound **78**: 441.0786 Da at 5.96 min; for NIBR: 428.0719 Da at 6.31 min. Values represent the mean of three biological replicates, and error bars indicate the standard deviation (SD). Source data are provided as a Source Data file.

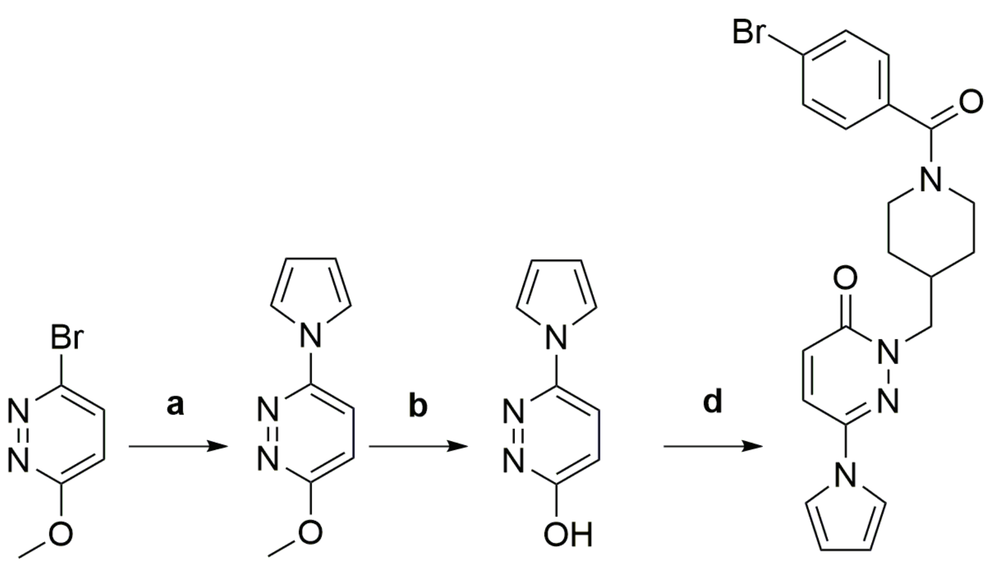

1 **2** **3** **105**

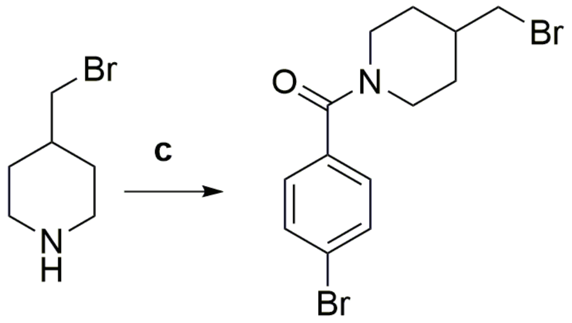

4 **5**

Supplementary Figure 12

Supplementary Figure 12. Chemical synthesis of 105. Please see Supplementary Data 2 for the spectra, NMR assignment: 1H NMR (500 MHz, DMSO) δ 1.14 – 1.29 (m, 2H), 1.35 – 1.87 (m, 2H), 2.07 – 2.23 (m, 1H), 2.64 – 3.22 (m, 2H), 3.35 – 3.70 (m, 1H), 3.95 (d, J = 7.3 Hz, 2H), 4.20 – 4.64 (m, 1H), 6.29 (t, J = 2.3 Hz, 2H), 7.12 (d, J = 9.7 Hz, 1H), 7.32 (d, J = 8.1 Hz, 2H), 7.45 (t, J = 2.3 Hz, 2H), 7.62 (d, J = 8.0 Hz, 2H), 7.97 (d, J = 9.9 Hz, 1H); 13C NMR (100 MHz, DMSO) δ 168.3, 159.0, 140.2, 135.9, 132.5, 131.3, 129.4, 129.1, 123.1, 118.7, 111.8, 55.6, 35.4. **a** A mixture of 3-bromo-6-methoxypyridazine (200.0 mg, 1.06 mmol), 1H-pyrrole (142.36 mg, 2.12 mmol), 1,10-phenanthroline (19.12 mg, 106.17 μmol), tripotassium phosphate (674.76 mg, 3.19 mmol) and copper(I) iodide (20.16 mg, 106.17 μmol) in degassed anhydrous toluene (10 mL) was heated under argon at 100 °C for 16 h. After cooling to room temperature, the reaction mixture was diluted with EtOAc (10 mL) and filtered. The filtrate was evaporated to give the crude 3-methoxy-6-(1H-pyrrol-1-yl)pyridazine (300.0 mg, 1.71 mmol, 70% purity), which was used in the next step without further purification.

b 3-Methoxy-6-(1H-pyrrol-1-yl)pyridazine (300.0 mg, 1.71 mmol), chlorotrimethylsilane (372.11 mg, 3.43 mmol), and sodium iodide (513.41 mg, 3.43 mmol) were mixed in MeCN and stirred at room temperature for 12 h. The reaction mixture was evaporated to give the crude 6-(1H-pyrrol-1-yl)-2,3-dihydropyridazin-3-one (600.0 mg, 3.72 mmol, 65% purity), which was used in the next step without further purification.

c 4-(Bromomethyl)piperidine hydrobromide (500.0 mg, 1.95 mmol) and 4-bromobenzoyl chloride (424.27 mg, 1.95 mmol) were mixed in DCM (20 mL) and cooled to 0 °C. Triethylamine (590.64 mg, 5.84 mmol) was added dropwise. The reaction mixture was stirred at room temperature for 30 min, diluted with DCM (20 mL), and washed with H_2O (3 × 10 mL). The organic layer was separated, dried over Na_2SO_4, and evaporated to give the 1-(4-bromobenzoyl)-4-(bromomethyl)piperidine (600.0 mg, 1.66 mmol, 85.3% yield, 95% purity) as a white solid.

d 6-(1H-pyrrol-1-yl)-2,3-dihydropyridazin-3-one (100.0 mg, 620.89 μmol), 1-(4-bromobenzoyl)-4-(bromomethyl)piperidine (222.86 mg, 620.86 μmol), and cesium carbonate (404.55 mg, 1.24 mmol) were mixed in DMF (5 mL) and stirred at room temperature for 12 h. The reaction mixture was filtered and purified by HPLC to give the 2-[1-(4-bromobenzoyl)piperidin-4-yl]methyl-6-(1H-pyrrol-1-yl)-2,3-dihydropyridazin-3-one (105, 31.5 mg, 71.38 μmol, 11.5% yield, 95% purity) as a white solid (ChemSketch 2021 2.0).

#	Product ID	Supplier name	Mass	Guaranteed purity (%)	Systematic name
1	P-34885727	Alinda Chemical Trade Company Ltd	412,44	90	5-(2,5-dioxo-1-phenylpyrrolidin-3-yl)-7-methyl-2-phenyl-2,3,4,5-tetrahydro-1H-isoindole-1,3-dione
2	P-595924689	Asinex	421,46	90	4-{2-[4-(2-fluorophenyl)-4-hydroxy-octahydro-1H-isoindol-2-yl]-2-oxoethyl}-1,2-dihydrophthalazin-1-one
3	P-595300349	Asinex	399,53	90	1-(3-{6-methyl-4-[(2-methylphenyl)methyl]pyridin-2-yl}piperidin-1-yl)-2-(pyridin-2-yl)ethan-1-one
4	P-595875427	Asinex	420,52	90	(2r,4'r)-4-methyl-N-(3-methyl-1,1-dioxo-1lambda6-thiolan-3-yl)-5-oxo-4,5-dihydro-3H-spiro[1,4-benzoxazepine-2,1'-cyclohexane]-4'-carboxamide
5	P-595296926	Asinex	418,49	90	2-[1-(5-cyclopropyl-1,2-oxazole-3-carbonyl)pyrrolidin-3-yl]-N-(propan-2-yl)quinoline-4-carboxamide
6	P-504640418	ChemBridge	408,54	90	N-[6,6-dimethyl-1-(4-methylphenyl)-4,5,6,7-tetrahydro-1H-indazol-4-yl]-3-(2-oxopiperidin-1-yl)propanamide
7	P-530922246	ChemBridge	392,45	90	N-{[1-(6-aminopyridine-2-carbonyl)piperidin-3-yl]methyl}-1-oxo-2,3-dihydro-1H-indene-4-carboxamide
8	P-427339043	ChemBridge	409,5	90	{1-[2-(4-methylphenyl)quinoline-4-carbonyl]pyrrolidin-3-yl}methanesulfonamide
9	P-530678478	ChemBridge	402,49	90	N-[(1-{2-phenyl-5H,6H,7H-cyclopenta[d]pyrimidin-4-yl}piperidin-3-yl)methyl]furan-2-carboxamide
10	P-7702457	ChemBridge	408,54	90	N-(1-hydroxy-3-methylbutan-2-yl)-1-{2-phenyl-5H,6H,7H-cyclopenta[d]pyrimidin-4-yl}piperidine-4-carboxamide
11	P-7751224	ChemBridge	416,52	90	1-{2-phenyl-5H,6H,7H-cyclopenta[d]pyrimidin-4-yl}-N-[2-(1H-pyrazol-4-yl)ethyl]piperidine-3-carboxamide
12	P-9492859	ChemBridge	405,45	90	N3-{3-[3-(4-methylphenyl)-1,2,4-oxadiazol-5-yl]phenyl}piperidine-1,3-dicarboxamide
13	P-594952895	ChemBridge	399,49	90	2-[(1-{3-[(1H-pyrazol-1-yl)methyl]benzoyl}piperidin-3-yl)methyl]-1H-1,3-benzodiazole
14	P-427013407	ChemBridge	388,5	90	4'-(1-cyanocyclopentyl)-N-[(oxan-2-yl)methyl]-[1,1'-biphenyl]-3-carboxamide
15	P-605512452	ChemBridge	395,49	90	N-{[(1R,3S)-3-[2-(3-methyl-1,2-oxazol-5-yl)acetamido]cyclohexyl]methyl}-2,3-dihydro-1H-indene-2-carboxamide
16	P-427263415	ChemBridge	377,44	90	3-(methoxymethyl)-1-[4'-(1H-1,2,3,4-tetrazol-5-yl)-[1,1'-biphenyl]-3-carbonyl]piperidine
17	P-7905918	ChemBridge	387,52	90	N-[1-(2,3-dihydro-1H-inden-2-yl)piperidin-3-yl]-3-(1H-indol-3-yl)propanamide
18	P-7820930	ChemBridge	394,48	90	1-cyclopentanecarbonyl-N-{4'-fluoro-[1,1'-biphenyl]-3-yl}piperidine-3-carboxamide
19	P-26838663	ChemBridge	380,48	90	4-methyl-3-[({2-[3-(2-methylphenyl)pyrrolidin-1-yl]ethyl}carbamoyl)amino]benzamide
20	P-9324910	ChemBridge	387,47	90	1-(but-3-enoyl)-N-[3-(1H-indol-2-yl)phenyl]piperidine-3-carboxamide
21	P-8663802	ChemBridge	408,54	44	N-(propan-2-yl)-1'-(quinoline-6-carbonyl)-[1,4'-bipiperidine]-4-carboxamide
22	P-8925469	ChemBridge	417,5	90	N-{3'-methyl-[1,1'-biphenyl]-3-yl}-1-[3-(1H-1,2,4-triazol-1-yl)propanoyl]piperidine-3-carboxamide
23	P-427147849	ChemBridge	415,49	90	2-methyl-4-phenyl-N-[3-(2H-1,2,3,4-tetrazol-2-yl)adamantan-1-yl]pyrimidine-5-carboxamide
24	P-530652983	ChemBridge	416,47	90	4-(4-methyl-1-oxo-1,2-dihydrophthalazin-2-yl)-N-{[3-(2-methylpropyl)-1,2-oxazol-5-yl]methyl}benzamide
25	P-504872747	ChemBridge	421,54		N-[1-(7-{[3-(furan-2-yl)phenyl]methyl}-5H,6H,7H,8H,9H-[1,2,4]triazolo[4,3-d][1,4]diazepin-3-yl)-3-methylbutyl]acetamide
26	P-27509019	ChemBridge	419,52	84	N-(2-hydroxyethyl)-1-[1-({2'-methyl-[1,1'-biphenyl]-3-yl}methyl)piperidin-3-yl]-1H-1,2,3-triazole-4-carboxamide
27	P-9085228	ChemBridge	418,46	77	N-[1-(2-fluorophenyl)-4,5,6,7-tetrahydro-1H-indazol-4-yl]-3-(2-oxopyrrolidin-1-yl)benzamide
28	P-8859137	ChemBridge	387,47	68	2-{3-[(3-hydroxy-3-phenylpyrrolidin-1-yl)methyl]phenyl}-3H,4H,5H,6H,7H-cyclopenta[d]pyrimidin-4-one
29	P-4005277	ChemBridge	420,51	90	2-(1,2-benzoxazol-3-yl)-1-{4-[2-methyl-6-(piperidin-1-yl)pyrimidin-4-yl]piperazin-1-yl}ethan-1-one
30	P-5757066	ChemBridge	412,42	90	4-methyl-N-(3-nitrophenyl)-3-[(pyridin-2-yl)sulfamoyl]benzamide
31	P-3911550	ChemBridge	404,46	90	4-(3-hydroxyphenyl)-3-methyl-1-[6-(piperidin-1-yl)pyridazin-3-yl]-1H,4H,5H,6H,7H-pyrazolo[3,4-b]pyridin-6-one
32	P-3506210	ChemDiv, Inc.	391,4	90	N-[3-(1,3-benzothiazol-2-yl)-4-hydroxyphenyl]-3-nitrobenzamide
33	P-34605096	Crea-Chim	423,49	90	N-(3-carbamoyl-4,5,6,7-tetrahydro-1-benzothiophen-2-yl)-7-methoxy-4H,5H-naphtho[2,1-d][1,2]oxazole-3-carboxamide
34	P-569439793	Eximed" Sp z o.o (Poland)	417,42	90	N-[3-(3-oxopiperazine-1-carbonyl)phenyl]-1H,4H-chromeno[4,3-c]pyrazole-3-carboxamide
35	P-579546742	HTS Biochemie Innovationen GmbH	423,89	95	N-{1-[3-(4-chlorophenyl)-1H-pyrazol-5-yl]pyrrolidin-3-yl}-4-acetamidobenzamide
36	P-579562941	HTS Biochemie Innovationen GmbH	395,5	90	N-[1-(2-methyl-5,6,7,8-tetrahydroquinazolin-4-yl)piperidin-4-yl]-4,5,6,7-tetrahydro-1,2-benzoxazole-3-carboxamide
37	P-579567992	HTS Biochemie Innovationen GmbH	424,45	90	1-({[1,1'-biphenyl]-4-yl}methyl)-2-hydroxy-4-methyl-4-[3-(1H-1,2,3,4-tetrazol-1-yl)phenyl]-4,5-dihydro-1H-imidazol-5-one
38	P-579562963	HTS Biochemie Innovationen GmbH	418,49	90	N-[1-(2-methyl-5,6,7,8-tetrahydroquinazolin-4-yl)piperidin-4-yl]-4-oxo-3,4-dihydrophthalazine-1-carboxamide
39	P-579552205	HTS Biochemie Innovationen GmbH	416,43	95	5-{1-[1-(3-methoxybenzoyl)pyrrolidin-3-yl]-1H-1,2,3-triazol-4-yl}-3-phenyl-1,2,4-oxadiazole
40	P-14719069	Life Chemicals Europe GmbH	424,45	90	1-{3-[5-(2H-1,3-benzodioxol-5-yl)-1,3,4-oxadiazol-2-yl]piperidin-1-yl}-3-(3,5-dimethyl-1,2-oxazol-4-yl)propan-1-one
41	P-460699271	Life Chemicals Europe GmbH	423,47	90	N-[2-(1H-indol-3-yl)ethyl]-4-[(4-oxo-3,4-dihydro-1,2,3-benzotriazin-3-yl)methyl]benzamide
42	P-14724018	Life Chemicals Europe GmbH	415,49	90	N-(2-carbamoylphenyl)-1-[6-(2-methylphenyl)pyridazin-3-yl]piperidine-4-carboxamide
43	P-579746759	Life Chemicals Europe GmbH	413,47	90	2-{[1-(naphthalene-2-carbonyl)piperidin-4-yl]methyl}-6-(1H-pyrazol-1-yl)-2,3-dihydropyridazin-3-one
44	P-460970630	Life Chemicals Europe GmbH	416,48	90	1-{5H,6H,7H-cyclopenta[c]pyridazin-3-yl}-N-[2-(6-hydroxypyridazin-3-yl)phenyl]piperidine-3-carboxamide
45	P-14367269	Life Chemicals Europe GmbH	419,43	90	3-methyl-N-{3-[6-(morpholin-4-yl)pyridazin-3-yl]phenyl}-4-nitrobenzamide
46	P-14297035	Life Chemicals Europe GmbH	397,38	90	N-(3-{3-methyl-[1,2,4]triazolo[4,3-b]pyridazin-6-yl}phenyl)-2-oxo-2H-chromene-3-carboxamide
47	P-25773492	Life Chemicals Europe GmbH	409,52	90	N-{1-[1-(3,4-dimethylphenyl)-1H-1,2,3-triazole-4-carbonyl]piperidin-4-yl}cyclohexanecarboxamide
48	P-14678454	Life Chemicals Europe GmbH	417,5	90	1-{4-[2-(4-hydroxypiperidin-1-yl)-2-oxoethyl]phenyl}-3-[(naphthalen-1-yl)methyl]urea
49	P-14631683	Life Chemicals Europe GmbH	423,89	90	3-chloro-N-{1-[1-(2-methylphenyl)-1H-1,2,3-triazole-4-carbonyl]piperidin-4-yl}benzamide
50	P-12972995	OTAVA CHEMICALS MB	424,4	90	N-(9,10-dioxo-9,10-dihydroanthracen-1-yl)-3-(2,5-dioxopyrrolidin-1-yl)benzamide
51	P-3126272	VITAS M CHEMICAL LIMITED	421,58	90	N-cyclooctyl-3-({5,5-dioxo-3aH,4H,6H,6aH-5lambda6-thieno[3,4-d][1,3]thiazol-2-yl}amino)benzamide
52	P-765826	VITAS M CHEMICAL LIMITED	388,37	90	2-(3,4-dimethylphenyl)-5-(3-nitrophenoxy)-2,3-dihydro-1H-isoindole-1,3-dione
53	P-578059675	VITAS M CHEMICAL LIMITED	403,39	90	(8S)-6-{[(3-nitrophenyl)methylidene]amino}-3,6,17-triazatetracyclo[8.7.0.0^{3,8}.0^{11,16}]heptadeca-1(10),11,13,15-tetraene-4,7-dione
54	P-617786942	VITAS M CHEMICAL LIMITED	399,4	90	7-(4-nitrobenzoyl)-4-phenyl-8-oxa-5,6-diazatricyclo[7.4.0.0^{2,6}]trideca-1(13),4,9,11-tetraene
55	P-2081630	VITAS M CHEMICAL LIMITED	417,48	90	4-{[(1,1-dioxo-1lambda6-thiolan-3-yl)carbamoyl]amino}-N-(2-hydroxy-2-phenylethyl)benzamide
56	P-1610467	VITAS M CHEMICAL LIMITED	402,49	90	2-hydrazinyl-3-(3-methoxyphenyl)-4,6-dihydro-3H-spiro[benzo[h]quinazoline-5,1'-cyclohexan]-4-one
57	P-884544	VITAS M CHEMICAL LIMITED	424,4	90	N-(2-methyl-1,3-dioxo-2,3-dihydro-1H-isoindol-5-yl)-4-(2-oxo-2H-chromen-3-yl)benzamide
58	P-35139778	VITAS M CHEMICAL LIMITED	413,47	90	1-(2H-indazol-3-yl)-5-oxo-N-(2,3,4,9-tetrahydro-1H-carbazol-1-yl)pyrrolidine-3-carboxamide
59	P-424784999	VITAS M CHEMICAL LIMITED	408,5	90	N-(6-methoxy-2,3,4,9-tetrahydro-1H-carbazol-1-yl)-4-[(1H-1,2,3,4-tetrazol-1-yl)methyl]cyclohexane-1-carboxamide
60	P-25060286	VITAS M CHEMICAL LIMITED	418,53	90	N-(2,6-dimethylphenyl)-4-{3,5-dioxo-4-azatetracyclo[5.3.2.0^{2,6}.0^{8,10}]dodec-11-en-4-yl}cyclohexane-1-carboxamide
61	P-1984120	VITAS M CHEMICAL LIMITED	406,43	90	2-[2-({3-methyl-6-oxo-6H,7H,8H,9H,10H-cyclohexa[c]chromen-1-yl}oxy)acetamido]benzamide
62	P-25061481	VITAS M CHEMICAL LIMITED	407,42	90	2-(1,3-dioxo-5-phenyl-octahydro-1H-isoindol-2-yl)-N-(3-nitrophenyl)acetamide
63	P-1685883	VITAS M CHEMICAL LIMITED	417,49	90	5-imino-6-[(naphthalen-1-yl)methylidene]-2-[2-oxo-2-(pyrrolidin-1-yl)ethyl]-5H,6H,7H-[1,3,4]thiadiazolo[3,2-a]pyrimidin-7-one
64	P-424719477	VITAS M CHEMICAL LIMITED	415,48	90	2-{3-[(1-methyl-2-oxo-2,3-dihydro-1H-indol-3-ylidene)methyl]-1H-indol-1-yl}-N-[(oxolan-2-yl)methyl]acetamide
65	P-578103928	VITAS M CHEMICAL LIMITED	413,47	90	(1S,7R)-N-[2-(1H-indol-3-yl)ethyl]-4-oxo-3-phenyl-10-oxa-3-azatricyclo[5.2.1.0^{1,5}]dec-8-ene-6-carboxamide
66	P-35115914	VITAS M CHEMICAL LIMITED	396,56	90	8-[(1,3-dimethyl-1H-pyrazol-4-yl)methylidene]-1-hydroxy-9a,11a-dimethyl-hexadecahydro-1H-cyclopenta[a]phenanthren-7-one
67	P-2768531	VITAS M CHEMICAL LIMITED	419,47	90	N-(1-hydroxy-3-methylbutan-2-yl)-2-[2-(1H-indol-3-yl)ethyl]-1,3-dioxo-2,3-dihydro-1H-isoindole-5-carboxamide
68	P-1295773	VITAS M CHEMICAL LIMITED	417,41	90	4-{3,5-dioxo-4-azatricyclo[5.2.1.0^{2,6}]dec-8-en-4-yl}-N-(4-methyl-2-nitrophenyl)benzamide
69	P-35111041	VITAS M CHEMICAL LIMITED	396,56	90	2-[(1,5-dimethyl-1H-pyrazol-4-yl)methylidene]-7-hydroxy-9a,11a-dimethyl-hexadecahydro-1H-cyclopenta[a]phenanthren-1-one
70	P-578085406	VITAS M CHEMICAL LIMITED	393,39	90	N-[(furan-2-yl)methyl]-19-oxo-1,11-diazapentacyclo[10.7.1.0^{2,7}.0^{8,20}.0^{13,18}]icosa-2,4,6,8(20),9,11,13(18),14,16-nonaene-10-carboxamide

Supplementary Figure 13

Supplementary Figure 13. List of the compounds selected from the VS.

#	Product ID	Supplier name	Mass	Guaranteed purity (%)	Systematic name
71	P-460971539	Life Chemicals Europe GmbH	402,45	90	2-{[1-(1H-indole-6-carbonyl)piperidin-4-yl]methyl}-6-(1H-pyrazol-1-yl)-2,3-dihydropyridazin-3-one
72	P-460971542	Life Chemicals Europe GmbH	408,46	90	2-({1-[6-(dimethylamino)pyridazine-3-carbonyl]piperidin-4-yl}methyl)-6-(1H-pyrazol-1-yl)-2,3-dihydropyridazin-3-one
73	P-460971632	Life Chemicals Europe GmbH	403,48	90	2-{[1-(2-cyclopropylbenzoyl)piperidin-4-yl]methyl}-6-(1H-pyrazol-1-yl)-2,3-dihydropyridazin-3-one
74	P-579742584	Life Chemicals Europe GmbH	377,44	90	2-{[1-(2-methylbenzoyl)piperidin-4-yl]methyl}-6-(1H-pyrazol-1-yl)-2,3-dihydropyridazin-3-one
75	P-579742976	Life Chemicals Europe GmbH	393,44	90	2-{[1-(4-methoxybenzoyl)piperidin-4-yl]methyl}-6-(1H-pyrazol-1-yl)-2,3-dihydropyridazin-3-one
76	P-579742702	Life Chemicals Europe GmbH	381,4	90	2-{[1-(2-fluorobenzoyl)piperidin-4-yl]methyl}-6-(1H-pyrazol-1-yl)-2,3-dihydropyridazin-3-one
77	P-579743969	Life Chemicals Europe GmbH	414,46	90	6-(1H-pyrazol-1-yl)-2-{[1-(quinoline-2-carbonyl)piperidin-4-yl]methyl}-2,3-dihydropyridazin-3-one
78	P-579743381	Life Chemicals Europe GmbH	442,31	90	2-{[1-(4-bromobenzoyl)piperidin-4-yl]methyl}-6-(1H-pyrazol-1-yl)-2,3-dihydropyridazin-3-one
79	P-460719106	Life Chemicals Europe GmbH	414,46	90	2-{[1-(isoquinoline-1-carbonyl)piperidin-4-yl]methyl}-6-(1H-pyrazol-1-yl)-2,3-dihydropyridazin-3-one
80	P-579743208	Life Chemicals Europe GmbH	411,88	90	2-({1-[2-(4-chlorophenyl)acetyl]piperidin-4-yl}methyl)-6-(1H-pyrazol-1-yl)-2,3-dihydropyridazin-3-one
81	P-460971635	Life Chemicals Europe GmbH	431,41	90	6-(1H-pyrazol-1-yl)-2-({1-[2-(trifluoromethyl)benzoyl]piperidin-4-yl}methyl)-2,3-dihydropyridazin-3-one
82	P-579742866	Life Chemicals Europe GmbH	397,86	90	2-{[1-(4-chlorobenzoyl)piperidin-4-yl]methyl}-6-(1H-pyrazol-1-yl)-2,3-dihydropyridazin-3-one
83	P-460719112	Life Chemicals Europe GmbH	404,43	90	2-{[1-(1H-1,2,3-benzotriazole-5-carbonyl)piperidin-4-yl]methyl}-6-(1H-pyrazol-1-yl)-2,3-dihydropyridazin-3-one
84	P-579742979	Life Chemicals Europe GmbH	393,44	90	2-{[1-(2-methoxybenzoyl)piperidin-4-yl]methyl}-6-(1H-pyrazol-1-yl)-2,3-dihydropyridazin-3-one
85	P-579743375	Life Chemicals Europe GmbH	427,88	90	2-{[1-(5-chloro-2-methoxybenzoyl)piperidin-4-yl]methyl}-6-(1H-pyrazol-1-yl)-2,3-dihydropyridazin-3-one
86	P-579742510	Life Chemicals Europe GmbH	363,41	90	2-[(1-benzoylpiperidin-4-yl)methyl]-6-(1H-pyrazol-1-yl)-2,3-dihydropyridazin-3-one
87	P-579743183	Life Chemicals Europe GmbH	431,41	90	6-(1H-pyrazol-1-yl)-2-({1-[3-(trifluoromethyl)benzoyl]piperidin-4-yl}methyl)-2,3-dihydropyridazin-3-one
88	P-579742587	Life Chemicals Europe GmbH	377,44	90	2-{[1-(4-methylbenzoyl)piperidin-4-yl]methyl}-6-(1H-pyrazol-1-yl)-2,3-dihydropyridazin-3-one
89	P-579742973	Life Chemicals Europe GmbH	393,44	90	2-{[1-(3-methoxybenzoyl)piperidin-4-yl]methyl}-6-(1H-pyrazol-1-yl)-2,3-dihydropyridazin-3-one
90	P-579742996	Life Chemicals Europe GmbH	395,43	90	2-({1-[2-(2-fluorophenyl)acetyl]piperidin-4-yl}methyl)-6-(1H-pyrazol-1-yl)-2,3-dihydropyridazin-3-one
91	P-460719103	Life Chemicals Europe GmbH	432,4	90	6-(1H-pyrazol-1-yl)-2-({1-[6-(trifluoromethyl)pyridine-3-carbonyl]piperidin-4-yl}methyl)-2,3-dihydropyridazin-3-one
92	P-579742891	Life Chemicals Europe GmbH	399,39	90	2-{[1-(2,4-difluorobenzoyl)piperidin-4-yl]methyl}-6-(1H-pyrazol-1-yl)-2,3-dihydropyridazin-3-one
93	P-579743378	Life Chemicals Europe GmbH	432,3	90	2-{[1-(2,4-dichlorobenzoyl)piperidin-4-yl]methyl}-6-(1H-pyrazol-1-yl)-2,3-dihydropyridazin-3-one
94	P-579746762	Life Chemicals Europe GmbH	413,47	90	2-{[1-(naphthalene-1-carbonyl)piperidin-4-yl]methyl}-6-(1H-pyrazol-1-yl)-2,3-dihydropyridazin-3-one
95	P-579743064	Life Chemicals Europe GmbH	406,48	90	2-({1-[4-(dimethylamino)benzoyl]piperidin-4-yl}methyl)-6-(1H-pyrazol-1-yl)-2,3-dihydropyridazin-3-one
96	P-460971533	Life Chemicals Europe GmbH	421,45	90	2-{[1-(2,3-dihydro-1,4-benzodioxine-5-carbonyl)piperidin-4-yl]methyl}-6-(1H-pyrazol-1-yl)-2,3-dihydropyridazin-3-one
97	P-579742660	Life Chemicals Europe GmbH	391,47	90	2-{[1-(3,4-dimethylbenzoyl)piperidin-4-yl]methyl}-6-(1H-pyrazol-1-yl)-2,3-dihydropyridazin-3-one
98	P-525736689	Life Chemicals Europe GmbH	402,45	90	2-{[1-(1H-indole-4-carbonyl)piperidin-4-yl]methyl}-6-(1H-pyrazol-1-yl)-2,3-dihydropyridazin-3-one
99	P-579742705	Life Chemicals Europe GmbH	381,4	90	2-{[1-(4-fluorobenzoyl)piperidin-4-yl]methyl}-6-(1H-pyrazol-1-yl)-2,3-dihydropyridazin-3-one
100	P-460719100	Life Chemicals Europe GmbH	420,49	90	2-{[1-(1,3-benzothiazole-6-carbonyl)piperidin-4-yl]methyl}-6-(1H-pyrazol-1-yl)-2,3-dihydropyridazin-3-one
101	P-579743211	Life Chemicals Europe GmbH	411,88	90	2-({1-[2-(2-chlorophenyl)acetyl]piperidin-4-yl}methyl)-6-(1H-pyrazol-1-yl)-2,3-dihydropyridazin-3-one
102	P-460719109	Life Chemicals Europe GmbH	415,45	90	6-(1H-pyrazol-1-yl)-2-{[1-(quinoxaline-6-carbonyl)piperidin-4-yl]methyl}-2,3-dihydropyridazin-3-one
103	P-579744896	Life Chemicals Europe GmbH	416,48	90	2-({1-[2-(1H-indol-1-yl)acetyl]piperidin-4-yl}methyl)-6-(1H-pyrazol-1-yl)-2,3-dihydropyridazin-3-one
104	P-460971536	Life Chemicals Europe GmbH	403,44	90	2-{[1-(1H-1,3-benzodiazole-5-carbonyl)piperidin-4-yl]methyl}-6-(1H-pyrazol-1-yl)-2,3-dihydropyridazin-3-one

Supplementary Figure 14

Supplementary Figure 14. **List of the compound selected from the hit expansion.**
