# Supplementary material for: Structure-based screening and a conformational biosensor identify a GPR183 inverse agonist and an activation switch
Source: Nat Commun. 2026 May 30;17:7020. doi: 10.1038/s41467-026-73857-9 (PMC13392048; doi:10.1038/s41467-026-73857-9)
Supplement: Supplementary file 2 — Description of Additional Supplementary Files [file 41467_2026_73857_MOESM2_ESM.docx]

**Description of Additional Supplementary Files**

File name: Supplementary Data 1

Description: Results of the SafetyScreen18 Core Panel – FR.

Fine name: Supplementary Data 2

Description: LC-MS of compounds 3 and 78, and LC-MS and NMR spectra of compounds 43 and 105.
