# Supplementary material for: Structure-based screening and a conformational biosensor identify a GPR183 inverse agonist and an activation switch
Source: Nat Commun. 2026 May 30;17:7020. doi: 10.1038/s41467-026-73857-9 (PMC13392048; doi:10.1038/s41467-026-73857-9)
Supplement: Supplementary file 4 — Supplementary Data 2 [file 41467_2026_73857_MOESM4_ESM.zip › Supplementary_data_file_spectra/43_LCMS.pdf]

-.o.-Syntez Purity Report -.o.-

Agilent 1100 LC/MSD SL
Diodearrey G1315B (DAD1A-215nm; DAD1B-241nm)
Mass Quad G1956B (MSD1-Pos, MSD2-Neg)
ELSD Altech 3300 (ADC1 A, ELSD)

Mobile Phase [illegible] 95 % [illegible]-0.1HCOOH
Separation column:
Rapid Resolutionn HT Cartige 4.6x30mm,
1.8-Micron, Zorbx SB-C18

compound 43

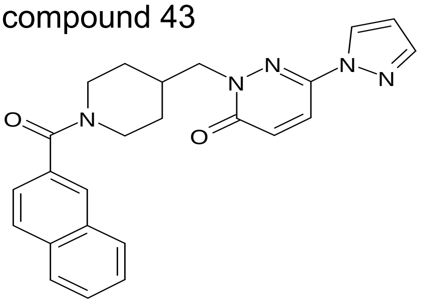

Mol.Weight: 413.48
Salt:

F6656-4129

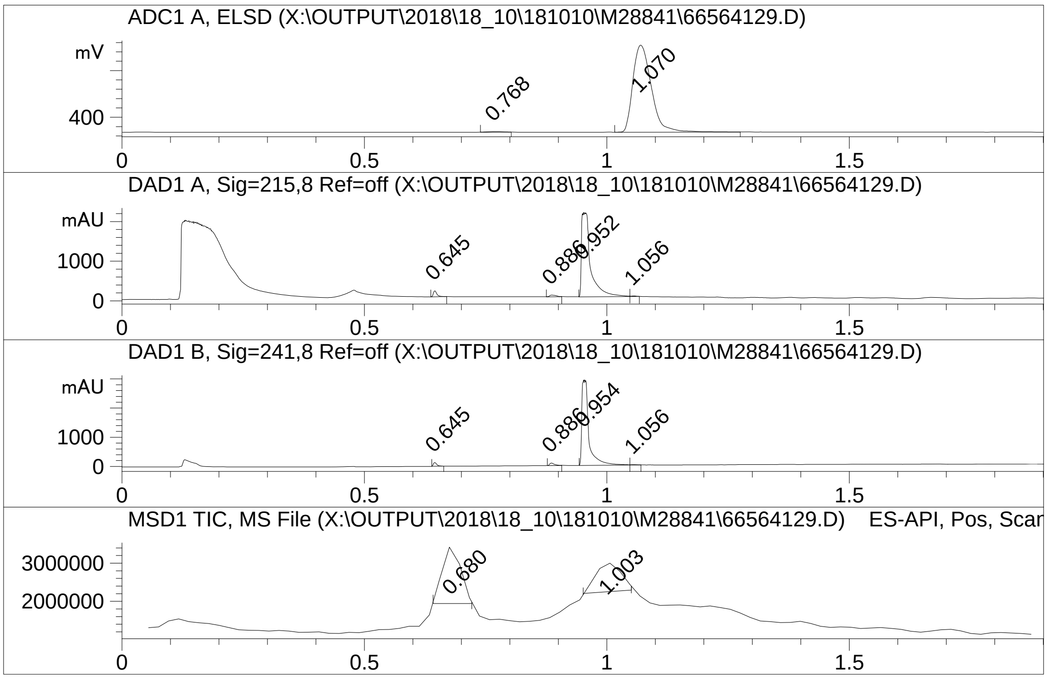

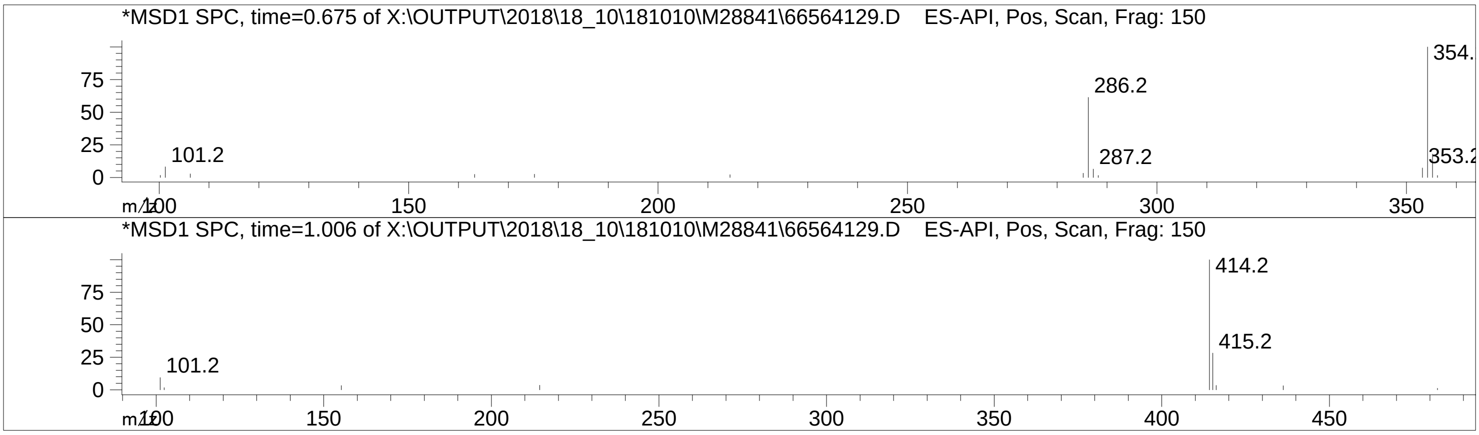

#	Signal	R.Time	Area %
1	ADC1 A, ELSD	0.768	0.523
2		1.070	99.477

#	Signal	R.Time	Area %
1	DAD1 A, Sig=215,8 Ref=off	0.645	2.564
2		0.886	1.406
3		0.952	95.660
4		1.056	0.370

#	Signal	R.Time	Area %
1	DAD1 B, Sig=241,8 Ref=off	0.645	2.126
2		0.886	2.045
3		0.954	95.460
4		1.056	0.368

#	Signal	R.Time	Area %
1	MSD1 TIC, MS File	0.680	57.942
2		1.003	42.058
