# Supplementary material for: Structure-based screening and a conformational biosensor identify a GPR183 inverse agonist and an activation switch
Source: Nat Commun. 2026 May 30;17:7020. doi: 10.1038/s41467-026-73857-9 (PMC13392048; doi:10.1038/s41467-026-73857-9)
Supplement: Supplementary file 4 — Supplementary Data 2 [file 41467_2026_73857_MOESM4_ESM.zip › Supplementary_data_file_spectra/78_LCMS.pdf]

-.o.-Syntez Purity Report -.o.-

Agilent 1200 LC/MSD SL
Diodearrey G1315B (DAD1A-215nm; DAD1B-241nm)
Mass Quad G6140A (MSD1-Pos, MSD2-Neg)
ELSD Altech 3300 (ADC1 A, ELSD)

Mobile Phase:A-H2O+0.1%HCOOH;B-MeCN+0.1HCOOH
Separati 99 %
Rapid Resolutionn HT Cartige 4.6x30mm,
1.8-Micron, Zorbax SB-C18

compound 78

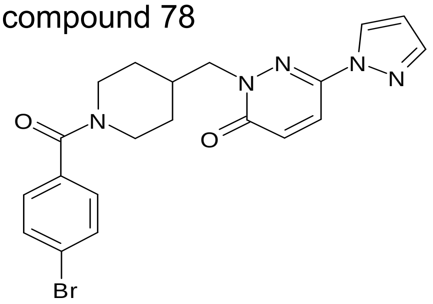

Mol.Weight: 442.32
Salt:

F6656-4127

M28826 ->

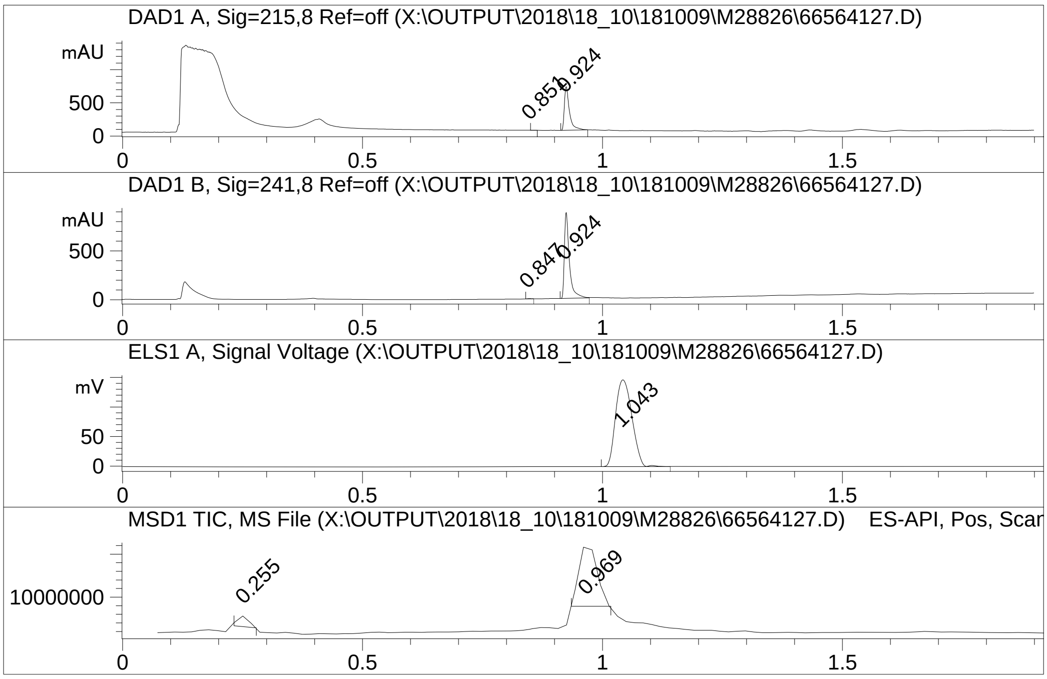

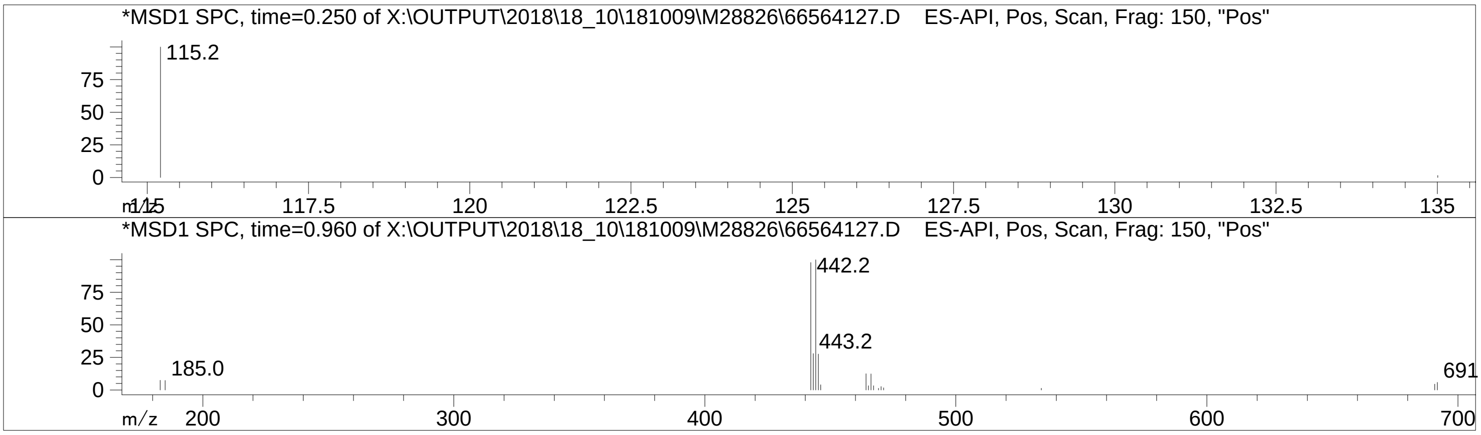

#	Signal	R.Time	Area %
1	DAD1 A, Sig=215,8 Ref=off	0.851	0.322
2		0.924	99.678

#	Signal	R.Time	Area %
1	DAD1 B, Sig=241,8 Ref=off	0.847	0.345
2		0.924	99.655

#	Signal	R.Time	Area %
1	ELS1 A, Signal Voltage	1.043	100.000

#	Signal	R.Time	Area %
1	MSD1 TIC, MS File	0.255	9.392
2		0.969	90.608
