# Supplementary figures and images for: Structure-based screening and a conformational biosensor identify a GPR183 inverse agonist and an activation switch
Source: Nat Commun. 2026 May 30;17:7020. doi: 10.1038/s41467-026-73857-9 (PMC13392048; doi:10.1038/s41467-026-73857-9)

MaxPeak: 100.00%  
Ret\_Time: 1.412 min

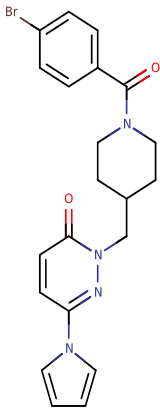

Mol Wt 441.32  
Exact Mass 442.11

| # | Time  | Area%  |
|---|-------|--------|
| 1 | 1.412 | 100.00 |

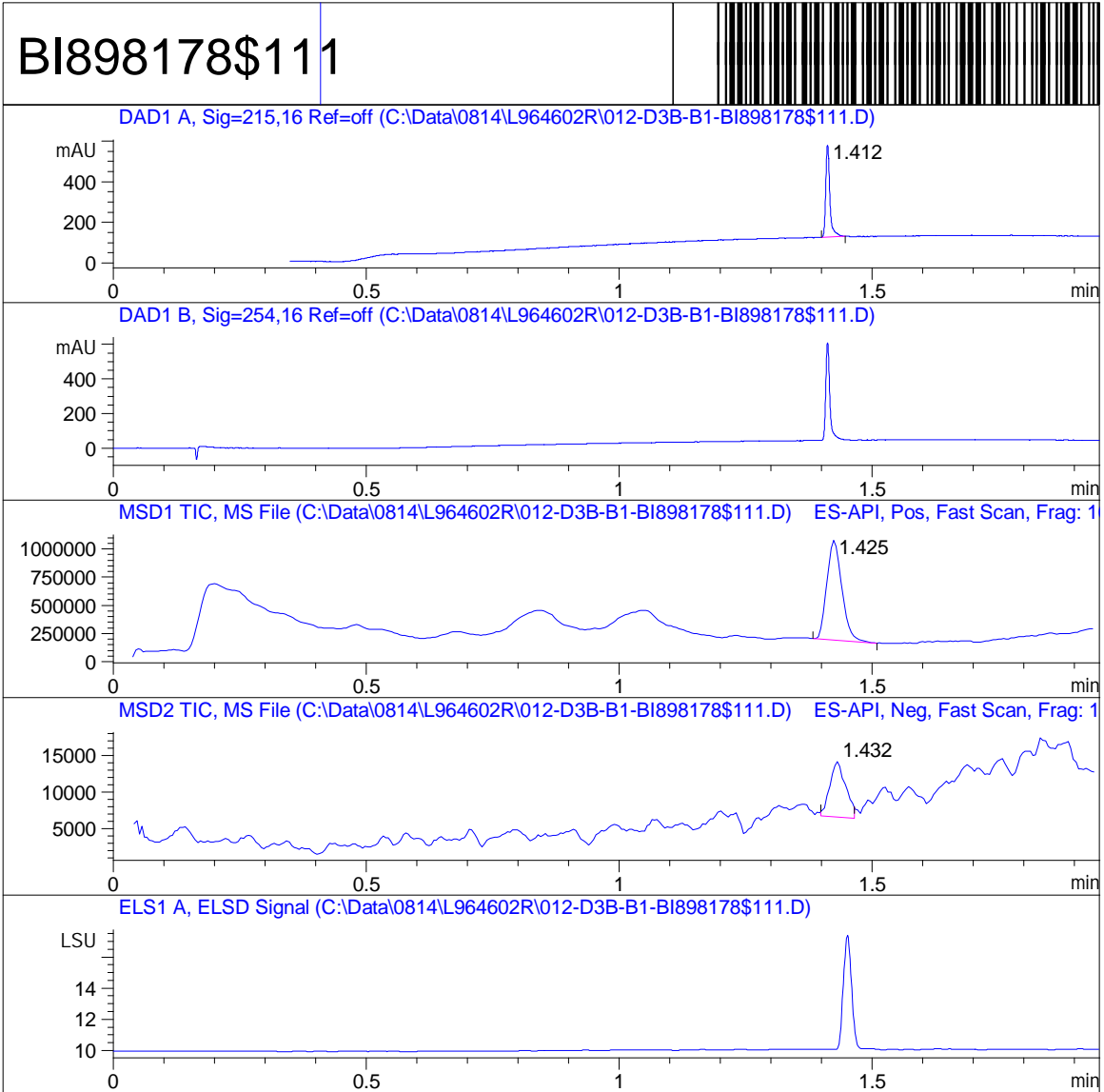

RT 1.425

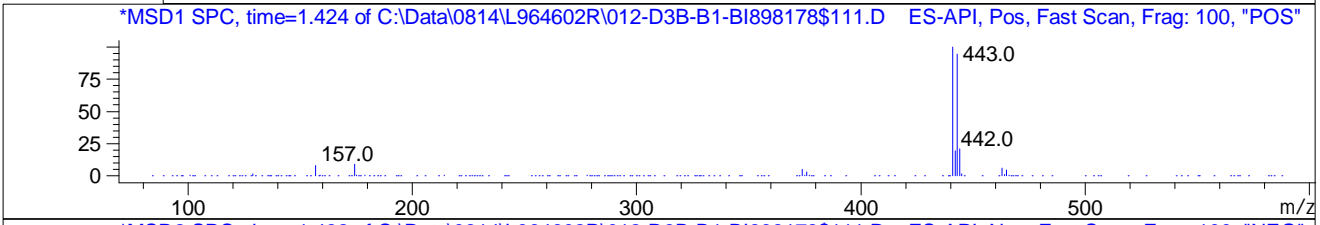

RT 1.432

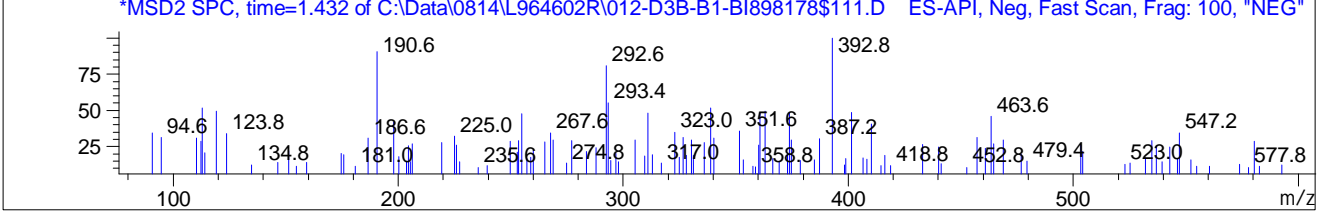

Supplement: Supplementary file 4 — Supplementary Data 2 [file 41467_2026_73857_MOESM4_ESM.zip › Supplementary_data_file_spectra/105_LCMS.PDF]

compound 3

C:\Temp\26204994

22.10.2008 01:26:36

RT: 0.00 - 4.00

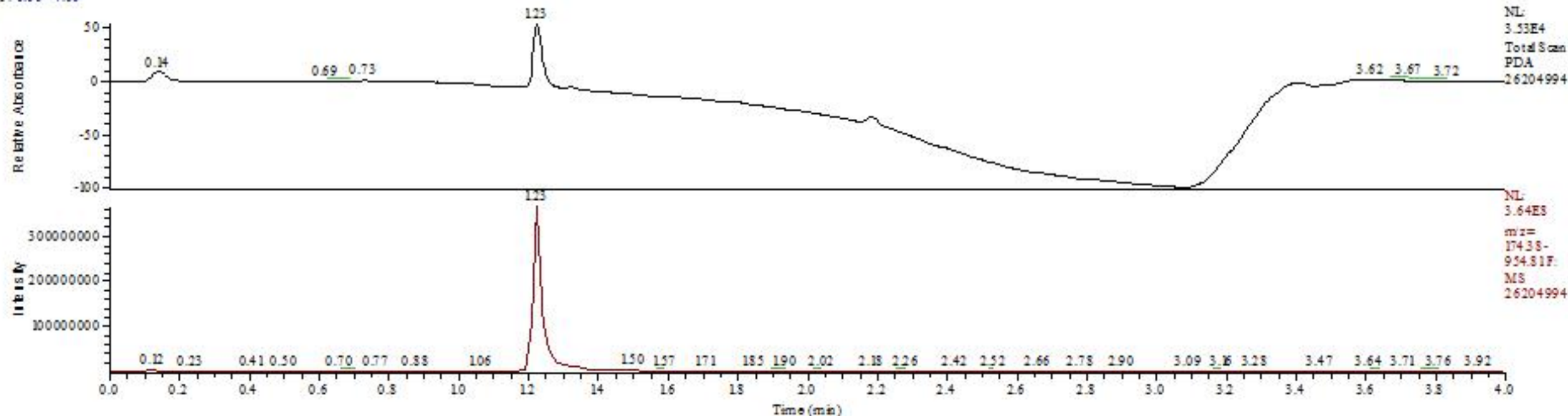

26204994#56-73 RT: 1.18-1.42 AV: E NL: 150E7  
T: +c APC Icorona Fullms [50.00-1000.00]

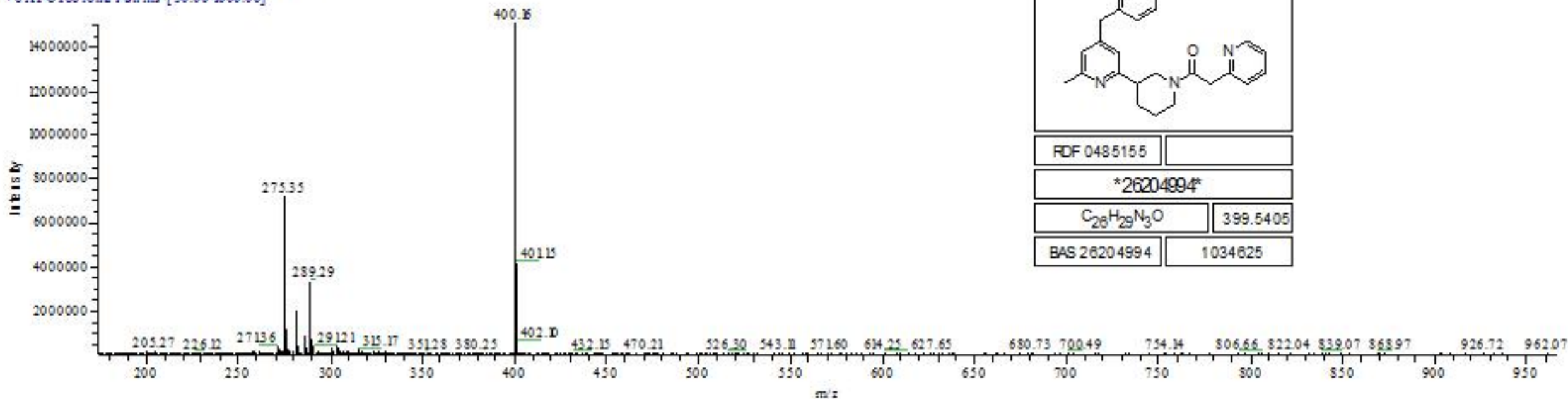

Supplement: Supplementary file 4 — Supplementary Data 2 [file 41467_2026_73857_MOESM4_ESM.zip › Supplementary_data_file_spectra/3_LCMS.pdf]
