# Supplementary material for: Structure-based screening and a conformational biosensor identify a GPR183 inverse agonist and an activation switch
Source: Nat Commun. 2026 May 30;17:7020. doi: 10.1038/s41467-026-73857-9 (PMC13392048; doi:10.1038/s41467-026-73857-9)
Supplement: Supplementary file 6 — Transparent Peer Review File [file 41467_2026_73857_MOESM6_ESM.pdf]

nature portfolio

Peer Review File

Structure-based screening and a conformational biosensor identify a GPR183 inverse agonist and an activation switch

Corresponding Author: Dr Pawel Kozielewicz

This file contains all reviewer reports in order by version, followed by all author rebuttals in order by version.

Version 0:

Reviewer comments:

Reviewer #1

(Remarks to the Author)
The manuscript by Andersson et al. reports the identification of a novel GPR183 ligand ("compound 78") using an AI-driven virtual screening pipeline combined with conformational biosensor assays. The authors also present a new BRET-based GPR183 conformational sensor and apply molecular dynamics plus mutagenesis to propose a binding mode. The integration of computational chemistry, mutagenesis, and functional pharmacology is commendable, and the work addresses a receptor of emerging therapeutic interest.

However, several aspects of the experimental design and interpretation substantially limit the strength of the conclusions and the claim that compound 78 is a "first-in-class" GPR183 inverse agonist. My main points are as follows:

1) In the Gi activation ebBRET assays, Rap1Gap-Rluc2 is employed as part of the readout. It is unclear whether the construct corresponds to Rap1Gap1a or Rap1Gap1b. This is not a trivial distinction: Rap1Gap1b contains both a GoLoco motif and GAP activity, both of which can intrinsically influence Gαi signaling independent of receptor activation. Without clarifying the isoform used and controlling for potential Rap1Gap–Gαi interactions, it is difficult to conclude that the observed effects are solely due to GPR183 modulation.
2) The data suggest that compound 78 reduces ebBRET signals and β-arrestin2 recruitment. However, given the possible confounding effects from the Rap1Gap-based assay design, and the lack of orthogonal validation using an assay free from GoLoco/GAP modulation, the assertion that the compound acts via GPR183-mediated Gαi inactivation may be an overstatement at this stage.
3) The functional potencies (pIC_{50} ~6.3) remain in the high-nanomolar to low-micromolar range, significantly lower than benchmark compounds such as NIBR189. While this is acceptable for a hit-stage molecule, the framing as "first-in-class" could be tempered to reflect the early stage of optimization.
4) The mNG–Nluc sensor is a creative tool for mechanistic probing. However, the reported Z-factor (<0.5) indicates it is not yet suitable for high-throughput or screening applications. The limitations of the sensor should be given more prominence in the main text rather than confined to the study limitations section.
5) Minor concern: the representation of BRET as AUC doesn't make sense. Data should be presented as net BRET and not % of ebBRET.

Recommendations for strengthening the manuscript:
- Clarify the Rap1Gap isoform used, provide rationale for its choice, and discuss its potential influence on basal and ligand-induced Gαi activity.
- Include at least one orthogonal G-protein activation assay that does not rely on Rap1Gap, to confirm that the observed effects are GPR183- and Gαi-dependent.
- Temper claims regarding "first-in-class" status and mechanism until such validation is provided.
- Consider repositioning the novelty of the work towards the identification of a new scaffold and the development of a GPR183 conformational sensor, rather than definitive mechanistic proof of GPR183 inverse agonism.

In summary, the manuscript contains interesting ideas and technical innovations, but the current evidence does not yet fully support the mechanistic claims. With the above clarifications and additional control experiments, the work would be considerably strengthened.

Reviewer #2

(Remarks to the Author)
This MS describes the identification of a GPR183 inverse agonist, compound 78, using a previously described AI-guided virtual screening pipeline, analog screening and some basic pharmacological assays. Compound 78 inhibits both constitutive and agonist-induced Gi signaling and reduces β-arrestin2 recruitment. Binding was supported by a BRET-based conformational sensor, molecular dynamics simulations, and mutagenesis studies, indicating interactions with key residues Y260 and H261. Compound 78 blocked migration of CD4+ T and B cells in response to the GPR183 agonist 7α,25-dihydroxycholesterol. The main novelty of the study is the introduces of a conformational sensor for live-cell monitoring of GPR183 activity. These findings provide a potential starting point for further development of GPR183-targeted compounds, but do not present a breakthrough in the field of GPCRs or in the area of GPR183 medicinal chemistry. The biological activity of the best compound, compound 78, is only very modest compared to several established GPR183 ligands, indicating that a lot of further chemical optimization is required. in fact, the presented study is more a hit finding effort, resulting in (sub)micromolar active molecules. No chemistry was done to optimize any of the molecules. In view of the known, highly active ligands in the field, this new hit can not be seen as breakthrough in the field.
Also selectivity profiling was limited to just one other receptor (CB1) and has therefore very limited value. No in vivo data were collected, so the compound’s pharmacokinetics and efficacy in disease models are unknown.

Technology-wise, the innovation is in my opinion limited. The applied computational tool has been published before, whereas the use of conformational GPCR sensors is also well-known (see e.g. work of Lohse & Shihada), although not for GPR183, but the GPR183 sensor was not of crucial importance for the disovery of the ligands. Actually, the sensor might be of future interest, but can be characterized much better with various different GPR183 ligands and perhaps even be furhter optimized an be used for screening purposes.

Reviewer #3

(Remarks to the Author)
This manuscript describes discovery of a novel inverse agonist for the GPCR GPR183. Using a novel machine learning drug discovery pipeline, the team screened 78 compounds and -- following expansion around the best hit -- found a compound with pIC50 of ~6 (compared to NIBR189's ~8-9). Additionally, the team developed a novel biosensor for rapidly measuring GPR183 inverse agonist activity. Validation of inverse agonist activity was performed using several ebFRET activity assays. A sensor system for agonist activity was additionally developed, matching results from previous assays. Molecular dynamics were used to explore the binding mode, and -- given a putative binding mode -- two mutations were characterized: one knocked out all activity, and the other appeared to have agonist activity, (presumably) binding the receptor in a different conformation.

My concern with this manuscript is that it appears to be two separate stories, one in which a new binder scaffold to GPR183 is discovered, and one in which new methods have been developed. However, both of these stories seem incomplete:
- on the methods side, a bespoke ligand discovery pipeline is created, and coupled with an AI-based virtual screening tool (PyRMD2Dock). But it is only applied to one system, and the role of the pipeline is not clear ... all they show is the pipeline was able to generate two hits out of 70. Further, they are starting with the cryoEM structure of a good binder in an already deep pocket, an easier example for computational drug discovery.
- on the compound side, while the compound is novel, its binding is modest, and the support that binding is matching design is extremely limited .. no structure is determined, and only one mutation is tested that blocks binding; this does not provide strong support that the ligand binds as designed. This lack of confidence in predicted structure makes the use of this as a starting point for lead optimization challenging. I'm not sure the MD data provided gives any additional support.

I think (at least) one of these two stories needs to be fully fleshed out before I can recommend publication.

Version 1:

Reviewer comments:

Reviewer #1

(Remarks to the Author)
The authors have put many efforts in answering to the reviewers' concerns. This has strengthened the manuscript and increased the overall impact of the current study.

Reviewer #2

(Remarks to the Author)
In this revised work, the authors have tried to improve their initial MS and included some new items. Yet, in my opinion this has not made the current MS easier to grasp.

The main addition to the MS are the extra data with cells endogenously expressing GPR183; here, very suprisingly, 78 is now as active as the reference compound NIBR and way more active than on all the shown biosensor assays. The difference is up to a 1000-fold and there is no sound explanation for this at all, except 2 lines in the discussion, talking about "potential system-dependent effects", with no efforts in trying to sort this out. To me, this really increases the confusion

around the activity of 78, which in the biosensor assays is active at micromolar concentrations, but affects migration at nanomolar levels. If this for whatever reason is true, what is then the actual value of the new biosensor?

Some additional minor points:

- in the introduction the authors report on earlier reported, potent GPR183 antagonists and mention what is holding them back in development (off-target effect etc), but claiming that 78 might be an option without showing proper selectivity panel tests (going broader that what is available in their own lab) and PK data is imo a bridge too far, as several of the competitor molecules at least where shown to be effective in in vivo models. Adding values for theoretical descriptors makes not real addition, the 3D liver metabolism data are a good addition, but still only in vitro,

- I mentioned before that the development of the sensor would be the most newest in the first version of the MS and that it deserved more characterization. The authors respond that there is hardly any ligand available and have added one. But please see the MedChem Express website for more.

- Before I also focussed on the lack of optimization of 78. Adding one new cmpd to this MS can certainly not be seen as lead optimization, I am sorry to say so. It does perhaps serve the point to show the interaction wiht Y651, but is no way 78 optimization. Details of chemistry are missing for this new compound (only a synthesis scheme is shown).

- the molecular pharmacology is in my opinion not really well-developed and deserves more attention by the authors. Assays preferably always include control curves for both agonist and antagonist. Some of the NIBR curves are really no curves, but show almost complete inhibition at all concentrations; pEC50/pIC50 values vary quite a lot and are also not very comparable between assays, etc... A typical example of the underdeveloped pharmacology: why use 1 uM of the agonist in the assay to test blockade by 78 and NIBR, while the agonist pEC50 = 9,6? And why is 78 here not able to compete with the agonist, like NIBR can? Also, the very small shift observed in the barrestin assays are not quantified..With 10 uM of 78 used in these assays, one could use the shifts in agonist pEC50 to calculate a pKb values....I think that such numbers will not be over 5 or so and will not match good with other values. Or, why use different concentrations of the agonist (50 or 100 nM) to test NIBR or GSK? This gives pIC50 values that can not be compared. And, there are unfortunatly other examples of pharmacology that does not really reads well.

Reviewer #3

(Remarks to the Author)
The revised manuscript addresses some but not all of my concerns from the original review.

The additional structural characterization and selectivity experiments do strengthen the structural conclusions of the manuscript, and provide more evidence that the molecule is binding as expected. I do believe the development of a novel biosensor has value.

I still think role of the bespoke computational pipeline/MD simulations is unclear, and it's general usefulness is overstated in the current manuscript. I'd like to see the language around this toned down a bit.

Version 2:

Reviewer comments:

Reviewer #4

(Remarks to the Author)
The authors present a nice study describing investigation of an inverse agonist for GPR183, including development of a conformational biosensor, various other established BRET assays, and molecular dynamics simulations and site-directed mutagenesis.
Reviewer 2 described multiple issues they have with the manuscript. Firstly, that it is difficult to grasp. I did not find this to be an issue.
The main issue reviewer 2 has with the manuscript is the varying potencies of compound 78 in different assays. The authors correctly state that potency is system- and assay-dependent, and potency differences across assays systems (as seen in this study) are reasonable and common. This is the concept of signalling bias and is widely understood and accepted within the field. The authors have addressed this well, both to Reviewer 2 and within the manuscript text itself.
That being said, it would be nice to have a table listing the different potencies observed, as this would give clarity to the various potencies across of the different assays. Likewise, all the potency and efficacy values obtained from Fig 3c would be better in a table rather than listed in the text. However, I appreciate that this manuscript has already been revised substantially over multiple iterations, so this is a “nice to have” not a “need to have” and I am not requesting that the authors include this at this late stage.

The other main issue that reviewer 2 has with the manuscript is that there is not enough medicinal chemistry/lead optimisation of compound 78. The authors have addressed this point by clarifying that such optimisation is beyond the scope of the manuscript. This is a reasonable position, and it appears that Reviewer 2 may have misunderstood the intent of the study.
Reviewer 2 has raised several other issues, and I feel the authors have responded reasonably to all these comments.
My only comment, separate to those raised by Reviewer 2, is that the text refers to Fig 3f and Fig 3g, however these are not present in the version of Fig 3 in the manuscript I received.

Open Access This Peer Review File is licensed under a Creative Commons Attribution 4.0 International License, which permits use, sharing, adaptation, distribution and reproduction in any medium or format, as long as you give appropriate credit to the original author(s) and the source, provide a link to the Creative Commons license, and indicate if changes were made.
In cases where reviewers are anonymous, credit should be given to 'Anonymous Referee' and the source.
The images or other third party material in this Peer Review File are included in the article's Creative Commons license, unless indicated otherwise in a credit line to the material. If material is not included in the article's Creative Commons license and your intended use is not permitted by statutory regulation or exceeds the permitted use, you will need to obtain permission directly from the copyright holder.
To view a copy of this license, visit https://creativecommons.org/licenses/by/4.0/

Rebuttal letter

We are grateful to the Editor and Reviewers for their thoughtful comments and constructive suggestions. We have carefully considered each point and undertaken extensive revisions, including new experiments and analyses, to address the concerns regarding mechanistic evidence, novelty, and narrative coherence. We believe that the revised manuscript is now substantially strengthened.

In this revision, we specifically address the three main issues highlighted by the reviewers and summarised in the editorial decision letter dated 19 September 2025.

Major changes to the points raised by the reviewers belonging to different categories can be summarised as:

1) Mechanistic evidence with data validation

a) New mechanistic evidence for ligand-receptor interaction: We added new data confirming the inverse agonist activity of compound 78 on the WT GPR183 and agonist-like behaviour on the Y260F$^{6.51}$ mutant.

b) New mechanistic insights into receptor activation: Y260(F)$^{6.51}$ acts as a molecular switch driving ligand-stimulated receptor bias.

c) Potency under physiological conditions: We have performed migration assays using a full concentration of 78 and reported a very high potency of the compound when the receptor is expressed endogenously.

d) Expanded selectivity profiling: It now includes SMO and β_2AR, along with negative controls in cells lacking overexpressed receptors.

e) New ligand analogue: Designed and synthesised based on the predicted binding mode, confirming the role of Y260$^{6.51}$ in ligand–receptor interactions.

f) New data validating the ebBRET-based experiments: We added an orthogonal Gi-activation assay (Gi-CASE), confirming the inverse agonist activity of compound 78 on GPR183 WT.

2) Novelty

Improved clarity of novelty: The manuscript now emphasises (i) tools: an AI-driven ligand screening platform and the first functional conformational biosensor for GPR183, (ii) a novel chemical scaffold with very high potency in blocking GPR183-mediated response under physiological conditions, and (iii) mechanistic insights into GPR183 activation mechanism and ligand bias.

3) Narrative coherence

Rewritten narrative: The ligand discovery, biosensor development/application, and insights into ligand-receptor interactions, as well as ligand-mediated receptor activation, are now presented as integrated components of a unified mechanistic study.

Our detailed answers to the comments made by each of the three reviewers are found below.

Reviewer #1 (Remarks to the Author):

The manuscript by Andersson et al. reports the identification of a novel GPR183 ligand ("compound 78") using an AI-driven virtual screening pipeline combined with conformational biosensor assays. The authors also present a new BRET-based GPR183 conformational sensor and apply molecular dynamics plus mutagenesis to propose a binding mode. The integration of computational chemistry, mutagenesis, and functional pharmacology is commendable, and the work addresses a receptor of emerging therapeutic interest.

We thank the reviewer for the positive and encouraging comments. We appreciate the recognition of our integrated approach, which combines AI-based screening, conformational biosensors, mutagenesis, and molecular dynamics to advance our understanding of GPR183 pharmacology.

However, several aspects of the experimental design and interpretation substantially limit the strength of the conclusions and the claim that compound 78 is a "first-in-class" GPR183 inverse agonist. My main points are as follows:

1) In the Gi activation ebBRET assays, Rap1Gap-Rluc2 is employed as part of the readout. It is unclear whether the construct corresponds to Rap1Gap1a or Rap1Gap1b. This is not a trivial distinction: Rap1Gap1b contains both a GoLoco motif and GAP activity, both of which can intrinsically influence Gαi signaling independent of receptor activation. Without clarifying the isoform used and controlling for potential Rap1Gap–Gαi interactions, it is difficult to conclude that the observed effects are solely due to GPR183 modulation.

We have used the Rap1Gap sequence previously published by Michel Bouvier's lab in https://elifesciences.org/articles/74101. The construct corresponds to Rap1Gap1a with three mutations to reduce potential Gs interactions (S437A/S439A/S441A). Clarification and reference have been added to the relevant method section.

See page 11

2) The data suggest that compound 78 reduces ebBRET signals and β-arrestin2 recruitment. However, given the possible confounding effects from the Rap1Gap-based assay design, and the lack of orthogonal validation using an assay free from GoLoco/GAP modulation, the assertion that the compound acts via GPR183-mediated Gαi inactivation may be an overstatement at this stage.

We have already included no-GPR183-transfected conditions in the first version of the manuscript, and observed no significant modulation of BRET between Rap1Gap-Rluc2 and rGFP-CAAX.

Additionally, we have now added an orthogonal assay using a Gi-CASE sensor. These results, obtained using the orthogonal assay, confirm the earlier data, which point to compound 78 as a GPR183 ligand.

Please see page 25 and Supplementary Figure 3d.

3) The functional potencies (pIC_{50} ~6.3) remain in the high-nanomolar to low-micromolar range, significantly lower than benchmark compounds such as NIBR189. While this is acceptable for a hit-stage molecule, the framing as "first-in-class" could be tempered to reflect the early stage of optimization.

The first-in-class referred to the conformational biosensor. However, the reviewer is right, and we have generally toned down the language to better reflect the data.

4) The mNG–Nluc sensor is a creative tool for mechanistic probing. However, the reported Z-factor (<0.5) indicates it is not yet suitable for high-throughput or screening applications. The limitations of the sensor should be given more prominence in the main text rather than confined to the study limitations section.

We have added more information to the discussion.

Please see page 26

5) Minor concern: the representation of BRET as AUC doesn't make sense. Data should be presented as net BRET and not % of ebBRET.

Here we respectfully disagree with the reviewer.

We are presenting net AUC from the kinetic curves from the respective experiments. First, we obtain %ΔBRET for each well and for each measurement upon applying baseline correction: 100*(value-baseline)/baseline, where baseline is defined as an average BRET from three BRET measurements prior to ligand addition.

Subsequently, to show ligand-treated data in relation to the vehicle-treated data, vehicle %ΔBRET data were subtracted from %ΔBRET for each ligand concentration/condition.

Using these baseline-corrected, vehicle-corrected %ΔBRET vs time kinetics plots, net AUC (negative peaks are subtracted from positive peaks) is calculated in GraphPhad Prism. This is, among many reasons, to avoid cherry picking of the most "representative" time point, and instead to represent, in our eyes, unbiased data analysis. To this end, we have generally not seen visible differences in kinetic parameters between the ligands.

To this end, the concentration response curves or some bar plots in the manuscript have net AUC (%ΔBRET or %ΔebBRET) on the y-axis – which, in relation to how the data were analysed, is fully correct.

Recommendations for strengthening the manuscript:

- Clarify the Rap1Gap isoform used, provide rationale for its choice, and discuss its potential influence on basal and ligand-induced Gαi activity.

- Include at least one orthogonal G-protein activation assay that does not rely on Rap1Gap, to confirm that the observed effects are GPR183- and Gαi-dependent.

- Temper claims regarding "first-in-class" status and mechanism until such validation is provided.

These comments have been addressed as stated above.

- Consider repositioning the novelty of the work towards the identification of a new scaffold and the development of a GPR183 conformational sensor, rather than definitive mechanistic proof of GPR183 inverse agonism.

We agree with the reviewer, and in light of the new exciting data, the manuscript has undergone a substantial modification. These modifications have been added to the text in various places of the manuscript.

In summary, the manuscript contains interesting ideas and technical innovations, but the current evidence does not yet fully support the mechanistic claims. With the above clarifications and additional control experiments, the work would be considerably strengthened.

Again, we thank the reviewer for positive comments and justified criticism, which, upon addressing, have substantially improved the study.

Reviewer #2 (Remarks to the Author):

This MS describes the identification of a GPR183 inverse agonist, compound 78, using a previously described AI-guided virtual screening pipeline, analog screening and some basic pharmacological assays. Compound 78 inhibits both constitutive and agonist-induced Gi signaling and reduces β-arrestin2 recruitment. Binding was supported by a BRET-based conformational sensor, molecular dynamics simulations, and mutagenesis studies, indicating interactions with key residues Y260 and H261. Compound 78 blocked migration of CD4+ T and B cells in response to the GPR183 agonist 7α,25-dihydroxycholesterol. The main novelty of the study is the introduces of a conformational sensor for live-cell monitoring of GPR183 activity.

We thank the reviewer for the accurate and thoughtful summary of our manuscript. We appreciate the recognition of our integrated approach combining AI-guided virtual screening, pharmacological characterisation, and molecular modelling to identify compound 78 as a GPR183 inverse agonist. We are also pleased that the reviewer highlights the development of our live-cell BRET-based conformational sensor, which we consider a key methodological advance that enables direct monitoring of GPR183 conformational dynamics in real time

These findings provide a potential starting point for further development of GPR183-targeted compounds, but do not present a breakthrough in the field of GPCRs or in the area of GPR183 medicinal chemistry. The biological activity of the best compound, compound 78, is only very modest compared to several established GPR183 ligands, indicating that a lot of further chemical optimization is required. in fact, the presented study is more a hit finding effort,

resulting in (sub)micromolar active molecules. No chemistry was done to optimize any of the molecules. In view of the known, highly active ligands in the field, this new hit can not be seen as breakthrough in the field.

We thank the reviewer for their thoughtful evaluation and agree that compound 78 represents an early-stage hit with a moderate potency in classical pharmacology assays. However, we have now shown that 78 exhibits very high potency in blocking the migration of agonist-stimulated GPR183, in the range of concentrations very similar to NIBR189, at physiological levels of agonist.

See page 36 and discussion

As for the lack of "chemistry" in the optimization step, we would like to outline that in the present work, we leveraged all the intrinsic advantages of running a VS campaign on large databases of commercially available compounds. In particular, after the first VS campaign that lead to the identification of 43 as a GPR183 binder, we used this chemotype as a seed to detect all the commercially available compounds that share a similar scaffold (reported in Supplementary Table 3). This allowed creating a second database enriched with all the compounds similar to the hit 43 found in the first VS. Here, by maintaining the 2-[(piperidin-4-yl)methyl]-6-(1H-pyrazol-1-yl)pyridazin-3(2H)-one scaffold, we explored the SAR around the terminal N-piperidin substituent. Compared to standard, expensive, and time-consuming medicinal chemistry studies (i.e. SARs studies) this approach has the advantage of rapidly providing a library of structural analogs of our initial hit compound and also being highly economically efficient.

Moreover, in the revised version of the manuscript, we have now designed and obtained an analogue 105 of the compound 78 based on its predicted docking pose, which confirms the proposed binding mode and underscores the crucial role of $Y260^{6.51}$ in ligand binding and receptor activation. This experimental result validates the computational pipeline and establishes a clear structure-activity link.

All that, coupled with the other data, clearly shows that 78 is in fact a breakthrough in the field:

- novel scaffold, unrelated to previously published GPR183 ligands,

- 78 can act as an inverse agonist, stabilising the inactive receptor conformation, but also as an agonist stabilising the active receptor conformation of a receptor mutant,

- application of 78 allowed for mechanistic insights into GPR183 and pinpointing $Y260^{6.51}$ as a crucial amino acid – this would not have been possible with e.g. NIBR189,

- 78 indeed shows moderate potency but good-to-very good efficacy in classical pharmacology assays,

- 78 exhibits very high potency in blocking GPR183-mediated PBMC migration.

Also selectivity profiling was limited to just one other receptor (CB1) and has therefore very limited value.

We agree with the reviewer that the initial selectivity panel was limited to CB_1R. We justify this choice mainly due to the nature of CB1R ligands being lipophilic and exhibiting a substantial basal activity. To address this, we have now expanded the profiling to include SMO, which is not only a Gi-coupled receptor but also a sterol-binding GPCR, making it a particularly relevant target for assessing the selectivity of the compound 78. In addition, we have included β_2AR as a representative classical GPCR control (class A).

In these additional experiments, compound 78 did not show a sustained specific Gi/Gs activation/inhibition mediated by these two GPCRs. There is indeed a weak and quickly decaying ebBRET induction for SMO which we interpret as:

1) An experimental artifact of a sample addition,

2) Signal increases, which is the opposite of what we show for GPR183, and since we are testing SMO, these results further point towards the artifact because literature tells us that measurements of agonist-stimulated SMO-mediated Gi activation have been relatively difficult to obtain even for validated SMO ligands, such as SAG1.3 or purmorphamine.

Importantly, in addition to the receptor-overexpressing conditions, we did not see a measurable modulation by compound 78 in the non-receptor overexpressed control, indicating a lack of activity at endogenously expressed receptors in HEK293A cells.

Furthermore, we tested the effect of the compound 78 on CCL5-induced migration of PBMCs and found no inhibition. Since CCL5 acts primarily via CCR5, but also engages CCR1, CCR3, and CCR4, these data further support the selectivity of compound 78.

Taken together, these results strengthen our conclusion that compound 78 is selective for GPR183 within the tested receptor set, and we believe the expanded dataset satisfactorily addresses the reviewer's concern.

See page 25

See Supplementary Fig. 3b

No in vivo data were collected, so the compound's pharmacokinetics and efficacy in disease models are unknown.

As compound 78 was developed primarily as a tool compound to investigate GPR183 activation mechanisms, extensive pharmacokinetic characterisation was not an initial objective. Nonetheless, to address this point of criticism, we have now performed in silico ADME profiling and included a metabolic stability assay in a 3D liver model, directly comparing compound 78 with NIBR189.

Both analyses indicate broadly comparable pharmacokinetic and metabolic stability properties for the two compounds, suggesting that compound 78 could reach the site of action (blood) at the applied concentrations in an intact form, and that it is predicted not to exhibit major ADME liabilities relative to the highest-potency, commercially available inverse agonist.

See page 36

See Supplementary Figure 13 and Supplementary Table 4.

Technology-wise, the innovation is in my opinion limited. The applied computational tool has been published before, whereas the use of conformational GPCR sensors is also well-known (see e.g. work of Lohse & Shihada), although not for GPR183, but the GPR183 sensor was not of crucial importance for the disovery of the ligands.

We thank the reviewer for this comment. We wish to clarify three points of innovation:

a. While the AI-based pipeline (PyRMD2Dock) was already developed by some of us, this manuscript reports its first successful application to a GPCR target. The fact that this pipeline yielded a ~20% hit rate (we identified 14 binders out of the 10M virtually screened ligands and 70 experimentally tested ones) serves as a significant validation of the method for the GPCR field, demonstrating its utility beyond soluble proteins in the case where very few ligands are already known.
b. Regarding the biosensor, we explicitly address the reviewer's point about previous work (e.g., Lohse & Schihada). We argue that the novelty lies precisely in our demonstration that standard sensor designs are not universally applicable. We constructed a GPR183 sensor based on the Halo-Nluc BRET, and found it failed to respond to agonist stimulation (Supplementary Figure 4d). This finding is significant as it underscores that "standard" architectures cannot simply be transferred to every receptor due to specific structural constraints, likely steric hindrance in the case of GPR183, as discussed in the text. Therefore, our development of the functional mNG-Nluc insertion sensor was not merely a routine application of known technology, but a necessary and non-obvious engineering solution required to monitor this specific receptor.
c. Finally, agonist binding to the Y260F$^{6.51}$ mutant could not be detected using conventional radioligand binding approaches, yet our biosensor data clearly demonstrate that binding can occur (as it also does for 78 and 105). Together with the data showing that Y260F$^{6.51}$ activates Gi (basal and agonist-induced) but does not recruit β-arrestin, the sensor was indeed instrumental to pinpointing this tyrosine at a position 260 as key in driving signaling bias. It should also be noted that, while we show here that the constitutive Gi activation of GPR183 WT is higher than that of Y260F$^{6.51}$, at similar cell surface expression levels, assessments of basal conformational state using the conformational biosensors revealed no differences in basal BRET between WT and the mutant. This indicates that differences in basal Gi activation by overexpressed GPR183 cannot be explained solely by basal receptor conformation. These findings would not have been possible without the conformational biosensor generated in this study.

Actually, the sensor might be of future interest, but can be characterized much better with various different GPR183 ligands and perhaps even be furhter optimized an be used for screening purposes.

We thank the reviewer for this constructive comment. We agree that the biosensor could be further characterised using a broader set of GPR183 ligands. However, only a few GPR183 ligands are currently commercially available, with only one agonist: 7α,25-dihydroxycholesterol. To expand the characterisation, we have now included data for

GSK682753A, another inverse agonist of GPR183, which further validates the responsiveness and sensitivity of the biosensor.

Importantly, using the biosensor of the Y260F$^{6.51}$ mutant, we have now included corresponding data for compound 78 and its new analogue 105 – both show agonist response. These additions strengthen the functional validation of the sensor, highlighting its versatility for both agonist and inverse agonist characterisation (only following receptor activation). We hope that these additions and clarifications sufficiently address the reviewer's concern and demonstrate the suitable robustness and versatility of the biosensor.

See Figures: 3, 7, Supplementary Figure 8

Reviewer #3 (Remarks to the Author):

This manuscript describes discovery of a novel inverse agonist for the GPCR GPR183. Using a novel machine learning drug discovery pipeline, the team screened 78 compounds and -- following expansion around the best hit -- found a compound with pIC50 of ~6 (compared to NIBR189's ~8-9). Additionally, the team developed a novel biosensor for rapidly measuring GPR183 inverse agonist activity. Validation of inverse agonist activity was performed using several ebFRET activity assays. A sensor system for agonist activity was additionally developed, matching results from previous assays. Molecular dynamics were used to explore the binding mode, and -- given a putative binding mode -- two mutations were characterized: one knocked out all activity, and the other appeared to have agonist activity, (presumably) binding the receptor in a different conformation.

We thank the reviewer for the accurate summary of our work. We appreciate the recognition of both the novel inverse agonist discovery and the development of the GPR183 biosensor, which together enabled detailed mechanistic insight.

We agree that compound 78 shows lower potency, in assays performed on HEK293 cells, than NIBR189 but provides a new scaffold and a functional tool to probe receptor activation. We are pleased that the reviewer acknowledged our combined use of computational modelling, mutagenesis, and biosensor assays, which together validate the proposed binding mode and receptor conformational changes.

Still, we would like to point out that, opposite to what the reviewer wrote:

- we did not develop the biosensor to measure inverse agonism,

- we did not use ebFRET, and we hope that the reviewer meant ebBRET.

My concern with this manuscript is that it appears to be two separate stories, one in which a new binder scaffold to GPR183 is discovered, and one in which new methods have been developed. However, both of these stories seem incomplete:

We thank the reviewer for this valuable comment. We partially agree that the initial version of the manuscript presented the ligand discovery and biosensor development as somewhat separate parts. To address this, the manuscript has been substantially revised - both to include new data and to improve the flow and coherence of the narrative - so that the ligand discovery and

biosensor characterisation are now presented as integrated components of the same complex study.

- on the methods side, a bespoke ligand discovery pipeline is created, and coupled with an AI-based virtual screening tool (PyRMD2Dock). But it is only applied to one system, and the role of the pipeline is not clear ... all they show is the pipeline was able to generate two hits out of 70. Further, they are starting with the cryoEM structure of a good binder in an already deep pocket, an easier example for computational drug discovery.

We wish to address any lack of clarity regarding the performance of the virtual screening campaign, hit rate, and target complexity.

Regarding the hit rate, the "two hits out of 70" figure refers only to compounds selected based on the most stringent statistical cutoff (one-way ANOVA with Dunnett's post-hoc analysis). However, as detailed in the Results section, applying a less stringent selection cut-off (LSD-Fisher post-hoc test) reveals that an additional 12 compounds met the criteria, yielding a total of 14 active compounds and a 20% hit rate. As we outline in the text, this success rate is fully in line with expectations for structure-based virtual screening campaigns.

Furthermore, we would like to emphasize that this manuscript focuses on the application of the PyRMD2Dock AI pipeline to the biological problem of GPR183 ligand discovery rather than benchmarking the method itself, which has been validated through a thorough statistical analysis elsewhere (REF). As the first application to a GPCR, this successful outcome validates the tool's utility for this transmembrane protein target class.

Finally, we respectfully disagree that GPR183 is an "easy example" simply due to its deep binding pocket. While pocket depth may facilitate binding over shallow ones, our Targeted Molecular Dynamics (TMD) simulations reveal that functional inactivation is complex, requiring a specific "hand-off" mechanism where the ligand must engage and translocate residue Y260$^{6.51}$ to break the Y112$^{3.33}$-Y260$^{6.51}$ lock. Identifying a molecule with the specific arrangement of H-bond acceptors required to trigger this conformational switch is a challenging task that was streamlined by the use of our AI-driven pipeline and subsequent mechanistic validation.

- on the compound side, while the compound is novel, its binding is modest, and the support that binding is matching design is extremely limited .. no structure is determined, and only one mutation is tested that blocks binding; this does not provide strong support that the ligand binds as designed. This lack of confidence in predicted structure makes the use of this as a starting point for lead optimization challenging. I'm not sure the MD data provided gives any additional support.

This is a critical point, and we have added extensive new data to address it. While we do not have a cryo-EM structure of compound 78, we provide a robust validation of the MD-predicted binding model through three lines of evidence:

First, we have now added one more mutation (C201A$^{5.43}$) to validate the MD simulations. Our MD simulations of the active state predicted key H-bonds with C201$^{5.43}$, Y260$^{6.51}$, and H261$^{6.52}$. As predicted, the H261$^{6.52}$ mutant completely abrogated compound 78's activity (see Figure 6d,

e). The C201$^{5.43}$ mutant also significantly modified the compound's effect (see Figure 6b, c). This strongly supports the predicted binding pose.

Second, the Y260$^{6.51}$ mutation did not just "block binding". It validated our Targeted MD (TMD) "hand-off" model presented in the revised version of the manuscript. The model predicted that Y260$^{6.51}$ is the key residue 78 "pulls on" to inactivate the receptor. Removing its H-bond capability (Y260F$^{6.51}$) converted compound 78 from an inverse agonist into a Gi-biased agonist. This switch in pharmacology (see Figure 7) is strong evidence for our model and the specific role of this residue.

Third, we believe the reviewer's concern about validation is definitely addressed by our 'forward engineering' approach. In the revised version of the manuscript, we did not rely solely on mutagenesis. Based on our MD model, we obtained a specific analogue, compound 105, designed to disrupt the predicted H-bond with Y260$^{6.51}$. As predicted by the "hand-off" mechanism, compound 105 (which has modified H-bond acceptors) still functioned as an inverse agonist on the WT receptor but had significantly weaker agonist efficacy on the Y260F$^{6.51}$ mutant compared to compound 78. This new data (Supplementary Figure 8) prospectively validates our computational model.

Taken together, the combination of detailed MD, validation by three distinct point mutations, and prospective validation with a newly synthesized analogue provides strong support for our binding model, far beyond a simple docking pose.

Regarding the reviewer's comment that the compound's binding is "modest," we wish to contextualize this within the scope of biological function.

While the affinity and potency in biophysical BRET assays are indeed in the high nanomolar/low micromolar range, these values do not fully predict the compound's physiological impact. Crucially, in primary human lymphocyte migration assays, which represent the most physiologically relevant readout for this receptor, 78 is equipotent to NIBR189, the current gold-standard high-potency inverse agonist. As shown in Figure 8e, 78 inhibited agonist-induced migration of PBMCs with a pIC_{50} of 8.82, indistinguishable from NIBR189 (pIC50= 8.89). Furthermore, it matched NIBR189's potency specifically in CD4+ T cells and B cells. This discrepancy suggests that complete receptor saturation (as seen with NIBR189) may not be necessary to achieve maximal therapeutic-like effects in complex biological systems. Therefore, despite "modest" biophysical metrics, 78 demonstrates high-value biological activity equal to the best-in-class compound, validating it as a highly relevant functional tool.

I think (at least) one of these two stories needs to be fully fleshed out before I can recommend publication.

We are looking forward to receiving this reviewer’s opinion about the revised manuscript.

Rebuttal Letter 2

We sincerely thank all the reviewers for their valuable comments and constructive criticism, which have significantly improved the quality of the manuscript.

Reviewer #1 (Remarks to the Author):

The authors have put many efforts in answering to the reviewers' concerns. This has strengthened the manuscript and increased the overall impact of the current study.

We thank the reviewer not only for the positive evaluation of our work, but also for the constructive and thoughtful comments on the initial version of the manuscript, which helped us to improve the clarity, rigor, and overall presentation of the study.

Reviewer #2 (Remarks to the Author):

In this revised work, the authors have tried to improve their initial MS and included some new items.

We note that this statement downplays and does not reflect the extensive revisions made in the Version 2, which we outline for clarity below.

1. MD simulations and *in silico* analysis of compound 78 bound to active GPR183 (4.5 µs total simulation time).

2. MD simulations and *in silico* analysis compound 78 bound to inactive GPR183 (4.5 µs total simulation time).

3. Targeted MD simulations supporting a proposed binding mode for 78, including a predicted *hand-off* mechanism.

4. ebBRET Gi activation assay data with compound 78-modulated activity of the GPR183 C201A$^{5.43}$ mutant.

5. Conformational biosensor data with compound 78-modulated activity of the GPR183 C201A$^{5.43}$ mutant.

6. Extended characterisation of Y260F$^{6.51}$ with Gi ebBRET sensor.

7. Extended characterisation of Y260F$^{6.51}$ with a conformational biosensor. *6* and *7* data show that compound 78 acts as an agonist on GPR183 Y260F$^{6.51}$, i.e., a clear switch in ligand efficacy from a negative efficacy on GPR183 WT to a positive efficacy on GPR183 Y260F$^{6.51}$.

8. Concentration–response migration assays showing that nanomolar concentrations of compound 78 and NIBR189 block migration of 7α,25-dihydroxycholesterol–attracted PBMCs (CD4$^+$, CD8$^+$, and B cells).

9. Synthesis and chemistry of compound 105.

10. Implementation of the Gi-CASE assay as an orthogonal readout to Gi ebBRET, directly addressing technical concerns raised by Reviewer 1 in the first round.

11. EbBRET Gi activation data with compound 78 and SMO to show lack of 78-driven SMO-mediated Gi activation.

12. EbBRET Gs activation data with compound 78 and β2AR to show lack of 78-driven β2AR -mediated Gs activation.

13. Concentration response of 7α,25-dihydroxycholesterol in a Gi ebBRET sensor paradigm.

14. Characterisation of an agonist activity of compound 105 on Y260F$^{6.51}$ with Gi ebBRET sensor.

15. Characterisation of an agonist activity of compound 105 on Y260F$^{6.51}$ with a conformational sensor.

16. EbBRET β-arrestin2 recruitment data showing that Y260F$^{6.51}$ does not recruit β-arrrestin2.

17. Cell surface expression data showing that GPR183 WT and Y260F$^{6.51}$ do not significantly differ in their cell surface expression levels.

18. Analysis of basal conformational state of GPR183 WT and Y260F$^{6.51}$ that demonstrated that their basal conformations are similar across different levels of expressions which suggest that the lower activity of Y260F$^{6.51}$ in the Gi activation assay may stem from a specific structural differences.

19. The ebBRET Gi activation data showing that NIBR189 is still acting as an inverse agonist on the Y260F$^{6.51}$ - we show here that using its scaffold we would not have been able to unravel the role of this residue in GPR183.

20. A metabolic stability assay performed in a human 3D liver model showing that NIBR189 and 78 have very similar metabolic stability profile.

21. *In silico* pharmacokinetic predictions for compound 78 and NIBR189.

These additions were presented as new figures and/or tables, and were accompanied by respective text additions to Methods, Results, Discussion and Conclusions sections.

In light of these extensive additions - spanning new simulations, new compounds, new orthogonal assays, expanded mechanistic validation, and functional/physiological readouts - we respectfully suggest that describing the revisions as merely “some new items” materially understates the extent of the work performed in response to the three reviews.

Yet, in my opinion this has not made the current MS easier to grasp.

We regret that the revised manuscript may still not have been fully clear to this reviewer, despite the substantial restructuring, additional explanations, and extensive new data included in Version 2. We respectfully note that the manuscript is intentionally framed as a tool- and mechanism-focused study aimed at elucidating GPR183 activation mechanisms, and signalling bias, rather than as a medicinal chemistry optimisation effort. It is therefore possible that some of the remaining concerns reflect differing expectations regarding the manuscript’s primary objectives and scope. We have nevertheless taken care in Version 3 to further clarify the presentation and strengthen the experimental support, and we hope that the revised manuscript now more clearly conveys both the rationale and the significance of the findings.

The main addition to the MS are the extra data with cells endogenously expressing GPR183; here, very

suprisingly, 78 is now as active as the reference compound NIBR and way more active than on all the shown biosensor assays.

Apparent activity can differ across assay formats due to differences in system gain, including receptor reserve and coupling efficiency. Importantly, endogenous expression typically entails lower receptor reserve than overexpression-based assay systems, which changes the relationship between occupancy and observed response and can shift apparent potency/efficacy between readouts.

As illustrated in Prof Terry Kenakin's - an internationally recognized expert in receptor pharmacology and former senior scientist in pharmaceutical drug discovery - textbook ([1], Figure 3.19), changes in receptor reserve can markedly alter the apparent magnitude of inverse agonism in a system without implying any inconsistency in the underlying ligand–receptor interaction. Accordingly, the differences observed between the endogenous functional endpoint and the biosensor assays reflect expected receptor-reserve/system-amplification effects rather than a technical or conceptual flaw in the dataset.

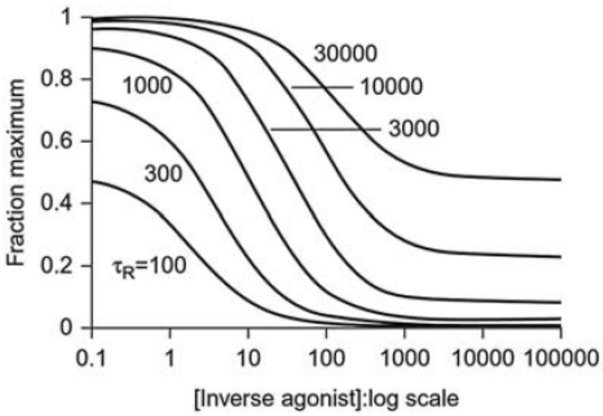

FIGURE 3.19 Inverse agonism as calculated by Eq. (3.22). Shown are curves for $\tau_R = 100$, 300, 1000, 3000, and 10,000 in a system of $L = 10^{-0}[G]/K_G = 10$. The inverse agonist has $\alpha = 0.03$ and $\gamma = 0.1$.

In the Figure 3.19 from the textbook (shared by Pawel Kozielewicz with permission from Elsevier), it is evident that the apparent potency of an inverse agonist decreases as receptor number increases. In this framework, τ_R denotes receptor reserve, which is larger in high-expression systems (e.g., overexpressed HEK293 cells). Thus, differences in apparent potency/efficacy across assay formats are an expected consequence of differing receptor reserve, rather than an inconsistency in the data. The functional validation of 78 in the PBMC migration assay would therefore represent the most leftward curve characterized by low receptor expression level and high inverse agonist potency.

To further support this point beyond textbook principles, we also performed additional experiments; the corresponding data are provided below for both the inverse agonist setup (left panels) and the competition setup (right panels) with 50 nM 7α,25-dihydroxycholesterol for 78 and NIBR 189.

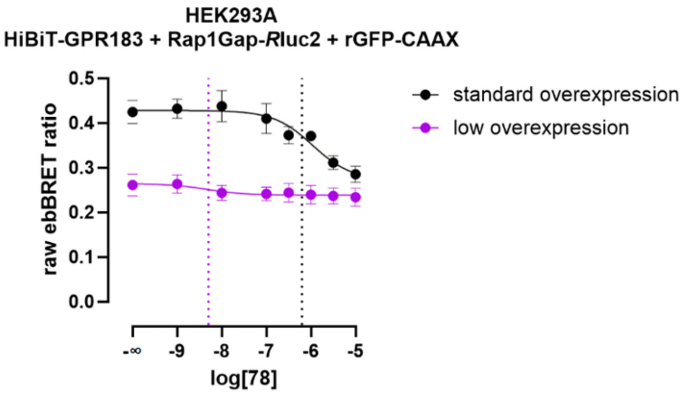

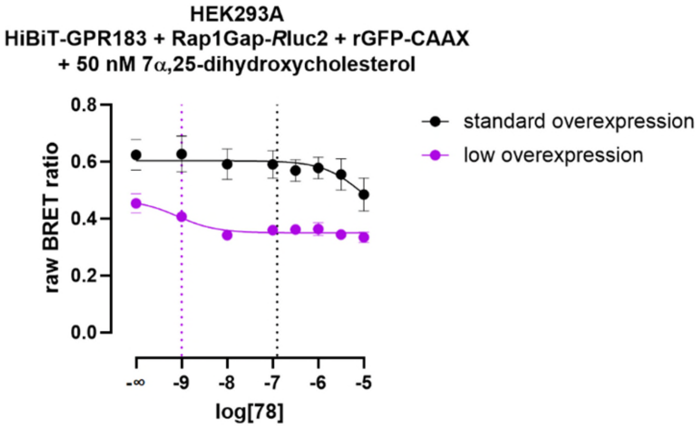

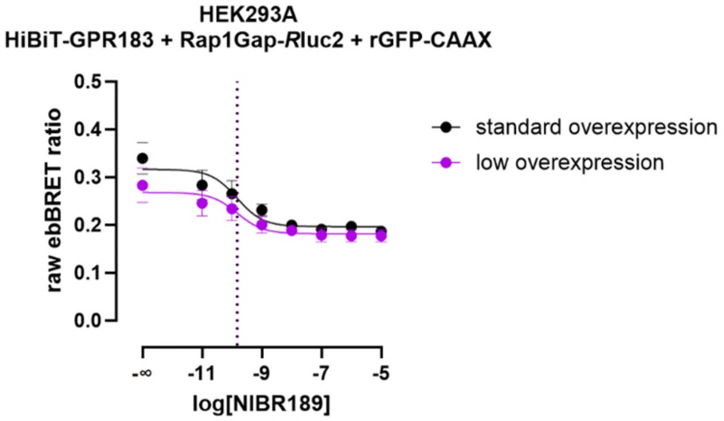

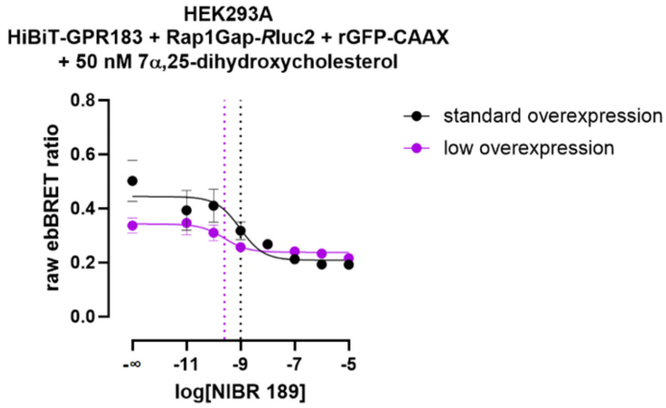

In these experiments, higher receptor expression is reflected by higher basal Gi activation (higher raw ebBRET ratios). The black and purple dashed lines mark the corresponding $logIC_{50}$ values. The data closely mirror the receptor-reserve principles illustrated in [1] (e.g., Fig. 3.19): changing receptor abundance changes system gain and therefore the apparent potency and efficacy of ligands in functional assays.

Consistent with this, compound 78 shows a marked leftward shift when receptor expression is lowered, i.e., it has a lower potency with visible partial inverse agonism at higher receptor expression levels, then it appears substantially more potent under conditions of reduced receptor reserve. By contrast, NIBR189 exhibits little or only a modest shift between expression levels, consistent with it already operating near the high-sensitivity end of the assay (high apparent potency under standard overexpression), such that further changes in receptor reserve have limited impact on the fitted IC_{50} under these conditions. We would also like to point the Reviewer to our previous publication in the *British Journal of Pharmacology* where we demonstrate and discuss changes in ligand binding profile in native vs overexpressed conditions [2].

Next, please also note that the pharmacology differences are not purely driven by the receptor reserve. In our study, we are measuring fundamentally different biological layers (e.g., receptor conformational

change or Gi activation vs cell migration), which occur on different timescales and are shaped by distinct degrees of signal amplification, detection methodology, cellular context, and, again, receptor reserve. Consistent with this, even assays reporting relatively proximal events can show marked potency shifts: for example, in FZD_5 activation/signalling, Figure S8 in [3] already shows ~1.5-log differences in agonist potency across readouts.

A more directly relevant example comes from a recent *Journal of Medicinal Chemistry* paper on GPR183 ligands, which we believe Reviewer 2 refers to as one of "our competitors" ([4]). Here, the hit compound 33 inhibits 20 nM agonist–induced migration with an IC_{50} of 0.73 nM, yet does not reach 50% inhibition of 100 nM agonist–induced TNFα upregulation even at 10 µM (more than 10000x/4-log shift; Figure 6C and 6E). This illustrates that endpoint-dependent differences - in apparent potency and in the ability to fully suppress responses - are common in GPCR pharmacology and reflect system/assay dependence (coupling efficiency, amplification, receptor reserve, and cellular context), rather than indicating an inherent inconsistency in the dataset. To clarify this point, we have now expanded the relevant discussion part.

The difference is up to a 1000-fold and there is no sound explanation for this at all, except 2 lines in the discussion, talking about "potential system-dependent effects", with no efforts in trying to sort this out.

We agree that, for some comparisons in Version 2, the difference approaches ~1000-fold. However, even this magnitude is not inherently anomalous in GPCR pharmacology, as already depicted with our data pasted into this Rebuttal Letter 2 and textbook information, and not even in GPR183 pharmacology (e.g. in [4] for compound 33 in migration vs qPCR assays, Figure 6C and 6E*;* in [5] Figure 1A and Figure 2C for 25-OHC). Large assay-dependent shifts can arise from well-established system-dependent factors, including receptor reserve/system gain, coupling efficiency, signal amplification, and differences in cellular context (endogenous vs. overexpression), and profile of a ligand, e.g. ligands with pathway bias are expected to display markedly higher activity in one signalling readout than another, sometimes spanning orders of magnitude (see review: [6]). Accordingly, the observed differences are fully consistent with established GPCR pharmacology rather than indicative of inconsistency or experimental artefact.

Moreover, the statement that this has "no sound explanation" and is addressed only in "two lines" is inaccurate. To be precise, we discussed these system-dependent effects in the Discussion in four lines (pasted below), and, now in Version 3, we extend it with a textbook knowledge and literature examples.

GPR183-targeting compound must consider its impact in this context. The strong migratory blockade observed for **78** likely reflects system-dependent effects in the PBMC assay, where endogenous receptor levels, signalling machinery, and expression balance differ from recombinant systems. It may also indicate that complete inhibition of GPR183-mediated Gi/β-arrestin signalling is not required to achieve full suppression of the migratory phenotype. While potent GPR183

To me, this really increases the confusion around the activity of 78, which in the biosensor assays is active at micromolar concentrations, but affects migration at nanomolar levels.

Reviewer 2 appears to treat the absolute IC_{50} values as directly comparable across fundamentally different assay systems. We respectfully disagree with that premise. In GPCR pharmacology, potency and efficacy are not intrinsic constants of a ligand; they are system- and assay-dependent and can shift substantially with receptor reserve/system gain, coupling efficiency, signal amplification, assay format (proximal conformational sensor vs downstream functional endpoint), and cellular context (endogenous vs. overexpression) ([7] particularly chapter 4). Accordingly, while it might be initially surprising, it is entirely plausible for compound 78 to show micromolar apparent potency in a conformational biosensor or ebBRET biosensor format yet produce nanomolar effects in a functional migration assay.

More generally, numerical values are contingent on experimental conditions (e.g., receptor expression level, assay window/dynamic range, incubation time, ligand depletion, and batch-to-batch variability). We are again referring to Terry Kenakin who wrote "*This is important as the type of detection system for drug activity determines which (or the absence of which) signal will be found, how much signal will be observed and how the signal producer will be characterized. Ultimately, the efficacy discovered with any given assay determines how the drug will be classified and what its use will be. Thus, efficacy is linked to the assay used to observe it, an effect described by Galandrin and Bouvier as pluridimensional efficacy*" ([8]). Thus, comparing compound activities across disparate readouts as if they were intrinsic ligand properties is not appropriate and can be misleading.

If this for whatever reason is true, what is then the actual value of the new biosensor?

The value of the biosensor is that it allows us to precisely measure ligand engagement with the orphan receptor. Next, it provides a proximal, conformational readout of GPR183 that is complementary to downstream functional assays. Specifically:

1. It enables direct detection of receptor conformational changes in real time, which has not previously been demonstrated for GPR183.

2. It allows detection of mutation-specific conformational signatures, including conformational changes in GPR183 $Y260F^{6.51}$, which likewise has not been shown before.

3. It reports the basal (ligand-unbound) conformational state of different receptor variants, enabling mechanistic comparisons that are not accessible with standard endpoint assays - or even with other conformational biosensor approaches (e.g., cpGFP, which is not ratiometric and typically requires stimulation to distinguish conformation-driven fluorescence changes from cell number/expression-driven effects).

Accordingly, the biosensor's value is not strictly to "match" IC_{50} values obtained in other functional assays, but to provide a mechanistic window into receptor conformational states and transitions that underpin signalling and bias.

More broadly, biosensors are used because they report different layers of receptor biology in live cells. Ligand-binding assays report occupancy/affinity; conformational biosensors report early structural transitions; and G protein/β-arrestin biosensors report partner engagement and transducer coupling. These readouts are not expected to yield identical potencies because they differ in coupling, amplification, and kinetics. Biosensors are also scalable, quantitative and kinetic, making them well-suited for screening and profiling ligands across receptor variants and signalling modalities. Please see here for reviews on these: e.g. [9], [10].

Some additional minor points:

- in the introduction the authors report on earlier reported, potent GPR183 antagonists and mention what is holding them back in development (off-target effect etc)

We agree that our original Introduction - by discussing previously reported potent GPR183 inverse agonists and development limitations - may inadvertently have conveyed a drug-discovery emphasis. To avoid any misunderstanding of the manuscript's aims, we have rewritten the Introduction to clearly foreground that this is a tool- and mechanism-driven study (with compound 78 used strictly as a tool compound), and we have toned down/removed framing around "development" considerations (e.g., off-target liabilities) that are not central to the work.

but claiming that 78 might be an option without showing proper selectivity panel tests (going broader that what is available in their own lab) and PK data is imo a bridge too far, as several of the competitor molecules at least where shown to be effective in in vivo models.

Reviewer 2's argument appears to be based on two recent medicinal chemistry lead-optimization papers on NIBR189-derived GPR183 ligands ([11] and [4]). Those studies indeed include broad selectivity/PK and *in vivo* efficacy packages - because their stated goal is to advance development candidates, e.g. "*Herein, we conducted a structural elaboration focusing on improving its PK and safety profile based on a reference antagonist NIBR189*" (*[4]*)

However, that is not the scope of our manuscript. Our work is explicitly tool- and mechanism-driven: we develop and apply a toolbox to identify and characterise compound 78 as a tool compound and to uncover mechanistic features of GPR183 activation and bias (including the Y260$^{6.51}$ switch and the efficacy switch of compound 78 on the corresponding mutant). As such, requests for *in vivo* PK studies or additional off-target profiling without any clear rationale for what constitutes a "suitable" panel size or target set, in our eyes, go beyond the scope of our work. Please note that we selected SMO, CB_1R and β2AR as selectivity controls with suitable justification, which can be found in Version 2 and in the Rebuttal Letter 1.

Still, to address Reviewer 2's request, we have now completed external commercial profiling of 10 μM compound 78 through Eurofins SafetyScreen 18 Core Panel. No notable off-target effects were observed across the tested GPCRs (Supplementary Data 2).

Regarding the other screened targets, the observed binding to hERG remained limited, with a predicted IC_{50} substantially above 10 μM, and compares very favourably with that of previously identified lead compounds. We observed that only $GABA_A$ ligand-gated ion channel might be affected by the compound 78. We have added this to the manuscript text: "*Next, to complement in vitro metabolic stability data, and our initial BRET-based selectivity assays (against overexpressed SMO, $β_2AR$, CB_1R and endogenous Gi and Gs-coupled GPCRs in HEK293A cells) as well as to provide an initial starting point for potential future optimisation, compound 78 (10 μM) was screened against a panel of GPCRs, kinase, ion channels, enzymes, and transporters using the Eurofins SafetyScreen18 Core Panel [67]. Importantly, no off-target activity was observed across the tested GPCRs (Supplementary Data 2). The observed 78 binding to hERG remained limited, with a predicted IC_{50} substantially above 10 μM, which, even taking into account differences in assaying methodologies [68], compares very favourably with that of previously identified lead compounds [24, 25], indicating that compound 78, which in the current study is used as a tool to interrogate GPR183 pharmacology, represents a very good starting point for potential future clinical development. As binding to an allosteric site was*

observed for the $GABA_A$ receptor, any future structure optimisation efforts should take this into consideration. Importantly, this does not affect the mechanistic interpretations, the key role of Y260 in GPR183 and the conclusions drawn from this study. Likewise, the data on the interaction of compound 78 with GPR183 are unaffected, as HEK293A cells do not express the $GABA_A$ receptor. Moreover, in physiological settings, the $GABA_A$ receptor is associated with the central nervous system, whereas GPR183 is primarily expressed on immune cells. In addition, because compound 78 is predicted to have only moderate brain penetration, any potential interaction with $GABA_A$ receptors in the brain would likely be limited."

Please see p. 39.

Adding values for theoretical descriptors makes not real addition, the 3D liver metabolism data are a good addition, but still only in vitro,

While we agree that assessments of metabolic stability in 3D human liver microtissues cannot fully replace *in vivo* PK studies, the presented data does demonstrate that the compound is metabolically stable with respect to human hepatic clearance, thereby paving the way for further preclinical development. This is particularly true as the utilized 3D human liver system has been shown to reliably detect hepatic clearance even for slowly metabolized drugs ([12, 13])

More broadly, the field is actively moving toward human-relevant experimental platforms to complement or, where possible, replace animal studies due to the increasing appreciation that rodent metabolism may have limited predictive value for human exposure, metabolism, and safety.

- I mentioned before that the development of the sensor would be the most newest in the first version of the MS and that it deserved more characterization. The authors respond that there is hardly any ligand available and have added one. But please see the MedChem Express website for more.

The reviewer's claim that we "added one" ligand is inaccurate – in fact incorrect. In Version 2 we added two additional compounds - GSK682753A and compound 105 - specifically to expand the pharmacological panel for sensor characterisation. Moreover, we tested compound 78 and compound 105 on the $Y260F^{6.51}$ mutant, where they behave as agonists, providing an additional and mechanistically informative validation scenario.

Reviewer 2 suggests consulting the MedChemExpress catalogue for additional ligands. We note, however, that MedChemExpress already lists compound 78 as a GPR183-targeting inverse agonist ("GPR183 inverse agonist-1" https://www.medchemexpress.com/gpr183-inverse-agonist-1.html, based on our preprint). This is therefore a somewhat contradictory suggestion: the reviewer expresses reservations about the activity/potency of compound 78 by writing "*To me, this really increases the confusion around the activity of 78*", yet directs us to a commercial source that independently markets the same compound as a GPR183 ligand. We do not cite vendor listings as scientific evidence, but this supports the selection of compound 78 as an accepted ligand.

Still, we have addressed the underlying request by expanding the agonist panel now in Version 3. Specifically, we added data for five additional agonists: three different oxysterols (from two original studies where multiple sterols were tested against GPR183 and several ascribed as its ligands [5, 14]) and two synthetic agonists (through collaboration). This substantially strengthens the sensor characterisation within the constraints of ligand availability and directly addresses the reviewer's call for broader testing. The manuscript now contains testing the mNG-Nluc biosensor with 10 GPR183 ligands (four hydroxycholesterols, TUG-2202, TUG-2292, NIBR 189, GSK682753A, 78, 105). Please see the new Fig. 3c.

- Before I also focussed on the lack of optimization of 78. Adding one new cmpd to this MS can certainly not be seen as lead optimization, I am sorry to say so.

We would like to reiterate that the manuscript is not focused on a lead-optimisation/medicinal chemistry. We do not present compound 78 as a lead to be optimised, and we do not claim that adding compound 105 constitutes "lead optimisation." Rather, the additional compound was included as a tool compound to strengthen the binding/activation hypothesis and to support the role of $Y260^{6.51}$ (including the efficacy switch on the mutant). Accordingly, the relevant question is whether the added compound(s) strengthen the mechanistic conclusions, not whether they represent a medicinal chemistry optimisation campaign.

It does perhaps serve the point to show the interaction wiht Y651,

We note first that the correct residue is $Y260^{6.51}$. Compound 105 was included specifically to test the proposed interaction network involving $Y260^{6.51}$ and to strengthen the mechanistic model (including the functional consequences of the $Y260F^{6.51}$ mutation). We therefore consider this point to be directly relevant and mechanistically informative.

but is no way 78 optimization.

This is not "compound 78 optimization" - because the manuscript does not claim to be a lead-optimisation or medicinal chemistry programme. The additional compound was included as a mechanistic tool to test specific hypotheses (not to improve ADME/PK, safety, or developability), and its role is to strengthen the mechanistic model involving $Y260^{6.51}$ and signalling bias rather than to "optimise" 78. To further address this point, we also refer to our comment from the previous rebuttal (Rebuttal Letter 1) copied below for convenience:

"As for the lack of "chemistry" in the optimization step, we would like to outline that in the present work, we leveraged all the intrinsic advantages of running a VS campaign on large databases of commercially available compounds. In particular, after the first VS campaign that lead to the identification of 43 as a GPR183 binder, we used this chemotype as a seed to detect all the commercially available compounds that share a similar scaffold (reported in Supplementary Table 3). This allowed creating a second database enriched with all the compounds similar to the hit 43 found in the first VS. Here, by maintaining the 2-[(piperidin-4-yl)methyl]-6-(1H-pyrazol-1-yl)pyridazin-3(2H)-one scaffold, we explored the SAR around the terminal N-piperidin substituent. Compared to standard, expensive, and time-consuming medicinal chemistry studies (i.e. SARs studies) this approach has the advantage of rapidly providing a library of structural analogs of our initial hit compound and also being highly economically efficient."

Details of chemistry are missing for this new compound (only a synthesis scheme is shown).

Reviewer 2's statement is incorrect. We have provided the structure of compound 105, a synthesis scheme with an accompanying written description (Supplementary Fig. 1), and we did attach the corresponding NMR and HPLC data in the data files submitted with the Version 2.

In addition, in Version 3 (accompanied with this Rebuttal Letter 2) we have now further strengthened the documentation by adding an assigned NMR description (peak assignments) for compound 105. This provides full transparency and exceeds the level of chemical characterisation typically expected for a

mechanistic/tool compound in this type of study – please see Methods and Supplementary Figure 1 legend.

- the molecular pharmacology is in my opinion not really well-developed and deserves more attention by the authors.

We note that this reviewer did not raise concerns about the molecular pharmacology in their comments on Version 1. These points are raised only in the second-round review.

Assays preferably always include control curves for both agonist and antagonist.

We had already addressed this. As described in the Methods and figure legends, responses are baseline- and vehicle-subtracted (as also explained in our response to Reviewer 1 in Rebuttal Letter 1). As such, there are "control curves" missing; rather these were utilized in the data analysis process.

In our study, ligand-based experiments are presented in two standard formats:

1. Single-compound conditions, where we show stimulation by agonists or reduction of constitutive activity by inverse agonists (we note that "antagonist" is a functional description that is assay/context-dependent).

2. Competition conditions, using a fixed concentration of the reference ligand - either an agonist (50 nM) or an inverse agonist (1, 3, or 10 µM) - while titrating the second compound.

In the competition plots, the $-\infty$ datapoints (as indicated in the respective figures) correspond to the conditions where only one of the two ligands is present at the stated concentration. Thus, the relevant agonist and inverse-agonist reference conditions are included within each dataset, and the necessary controls are explicitly defined in the legends and Methods.

Some of the NIBR curves are really no curves, but show almost complete inhibition at all concentrations;

This is a fair point. Some of the NIBR189 concentration - response traces are too steep/near-saturated over the tested range and therefore do not present as well-defined "curves." While our study is not focused on the well-established ligand NIBR189 and these traces do not affect the central mechanistic conclusions, we agree that clearer reference curves improve presentation and interpretability. We have therefore repeated these experiments using an expanded concentration range and additional intermediate concentrations to generate proper sigmoidal concentration–response curves, and we include the updated data in Version 3 – please see the new Fig 2c and Fig 2d.

We note that these changes do not affect the central conclusions of this study.

pEC50/pIC50 values vary quite a lot and are also not very comparable between assays, etc...

Reviewer 2 again notes that pEC_{50}/pIC_{50} values "vary quite a lot" and are "not very comparable between assays." We agree only with the second part - and this is precisely expected according to pharmacology textbook knowledge. Potency estimates (EC_{50}/IC_{50} and their log forms) are assay- and system-dependent and therefore should not be treated as directly comparable across different assay formats ([7], particularly chapter 4). As such, differences in receptor reserve/system gain, coupling efficiency, signal amplification, kinetics, and cellular context routinely produce substantial shifts in apparent potency between proximal biosensor readouts and downstream functional endpoints. Accordingly, variation in pEC_{50}/pIC_{50} across assays does not indicate a technical flaw; the appropriate

interpretation is whether each assay is internally consistent, well controlled, and mechanistically coherent within its own system parameters.

A typical example of the underdeveloped pharmacology: why use 1 uM of the agonist in the assay to test blockade by 78 and NIBR, while the agonist pEC50 = 9,6?

This is a fair point. For consistency - and to maintain a reliable assay window while avoiding unnecessarily supramaximal agonist concentrations - we have revised the blockade experiments to use 50 nM agonist in the in vitro assays (instead of 1 µM). The updated concentration better matches the agonist potency and provides a more appropriate dynamic range for quantifying inhibition by compound 78 and the reference ligand – please see the new Fig 2d .

We note that these changes do not affect the central conclusions of this study.

And why is 78 here not able to compete with the agonist, like NIBR can? –

Because compound 78 has lower potency (and, in this assay context, a slightly lower apparent efficacy) than the reference ligand NIBR189, it produces a smaller rightward shift of the agonist concentration–response and a less pronounced reduction in the maximal response in the Gi ebBRET assay. In other words, it can still oppose agonist signalling, but - given its lower potency and system-dependent transduction - its competition appears weaker under these conditions, resulting in a smaller change in both potency and Emax compared with the reference compound.

Also, the very small shift observed in the barrestin assays are not quantified.

Reviewer 2's statement is incorrect. The β-arrestin shifts are quantified in the manuscript: we report statistical significance directly in the text (extra sum-of-squares F test), with P = 0.0048 and P = 0.038 for the two independent experimental paradigms (see Fig. 2e in Version 2 and associated text pasted below). Accordingly, the shift is small but measurable and statistically supported, and it is explicitly quantified as such.

this scaffold protein to membrane-embedded and agonist-exposed GPR183. Here, we found that **78** right-shifted the agonist concentration response for β-arrestin2 recruitment in two independent experimental paradigms (extra sum-of-squares F test, P = 0.0048 and P = 0.038, respectively; **Fig. 2e**). The consistency of this small but detectable effect across distinct assay formats and BRET

With 10 uM of 78 used in these assays, one could use the shifts in agonist pEC50 to calculate a pKb values.

As a short side note, we calculated a pK_b value for compound 78 in the GPR183 conformational sensor assay, where the readout is used as a proxy for ligand binding. In contrast, for functional assays in which antagonist potency is inferred from shifts in agonist responses, the appropriate pharmacological parameter is pA_2 rather than pK_b ([1]; Chapter 7.3.1).

Next, it is generally preferable to estimate pA_2 from multiple antagonist concentrations (e.g., via Schild-type analysis), rather than relying on a single competition condition ([15], specifically chapter III. B.1; [1], chapter 7.3.1). Nevertheless, to address this Reviewer's comment, using the 10 µM condition, according to the Gaddum/Schild EC50 shift function in GraphPad Prism, the agonist right-shift allows an approximate estimate of potency, yielding pA_2 values of 5.45 and 5.27 for the Nluc-Venus and Rluc2-

rGFP assay formats, respectively. It can be seen in the ebBRET setup that the apparent efficacy of the agonist in the presence of 78 is lower (the data for Nluc-Venus present itself with lower window which may have precluded these outcomes). As such, the Schild slope deviates from unity which precludes, in addition to lack of more concentrations of 78, a classical Schild analysis.

In the new version of the manuscript, we have replaced that data with new data on a 78-mediated blockage of agonist-induced β-arrestin2 recruitment to an overexpressed GPR183. We have focused on an ebBRET setup given its much higher assay window than the direct Nluc-Venus setup. Here, we demonstrate that 78 shifts both the potency and the efficacy of the agonist. The data are analysed in the Results section, Fig 2f Supplementary Figure 4f.

I think that such numbers will not be over 5 or so and will not match good with other values.

The reviewer's speculation is incorrect, and as the raw data were provided with the submission, this could have been checked directly from the dataset (as stated above: the Gaddum/Schild EC50 shift function in GraphPad Prism, the agonist right-shift allows an approximate estimate of potency, yielding pA_2 values of 5.45 and 5.27 for the Nluc-Venus and Rluc2-rGFP assay formats, respectively).

Or, why use different concentrations of the agonist (50 or 100 nM) to test NIBR or GSK? This gives pIC50 values that can not be compared.

This is a fair point. Using different agonist concentrations (e.g., 50 vs 100 nM) can indeed complicate direct comparison of derived pIC_{50} values for different compounds in the same assay paradigms. For consistency, we have now standardised these inhibition experiments to 50 nM agonist across conditions (including NIBR and GSK), and we present the updated analyses accordingly. Please see new Fig 3C

We note that these changes do not affect the central conclusions of this study.

And, there are unfortunatly other examples of pharmacology that does not really reads well.

We have re-read the manuscript thoroughly and have addressed all specific pharmacology points raised elsewhere by all the Reviewers by updating the experimental design and analyses:

- We used the same 50 nM agonist concentration across all competition assays (with new data)

- We included additional lower concentrations of NIBR189 to ensure that the curves display two distinct plateaus (top and bottom) (new data)

- We adjusted the y-axis ranges to be consistent across identical assay paradigms within the same figures (e.g., Fig. 6).

- We added the unit "M" to the x-axes for logarithmic concentration scales

- We added detailed information about incubation times in the main text

- As mentioned above, we included new data demonstrating 78-mediated blockade of agonist-induced β-arrestin2 recruitment to overexpressed GPR183

- To maintain consistency with the data for $C201A^{5.43}$, $H261A^{6.52}$, and $Y260F^{6.51}$, we also added new data showing GPR183 WT Gi activation in response to agonist ± 10 μM 78. These results are now described and interpreted in the Results section. Importantly, the WT data confirm our previous findings for $C201A^{5.43}$: at higher concentrations of 7α,25-dihydroxycholesterol, compound 78 increases agonist efficacy. See new Fig. 2e and Supplementary Fig. 4d.

We feel that these comments and our subsequent changes and additions have substantially improved the manuscript.

Reviewer #3 (Remarks to the Author):

The revised manuscript addresses some but not all of my concerns from the original review.

The additional structural characterization and selectivity experiments do strengthen the structural conclusions of the manuscript, and provide more evidence that the molecule is binding as expected. I do believe the development of a novel biosensor has value.

We appreciate this assessment and understand it to mean that the key concern raised previously - namely that the manuscript appeared to present "two incomplete stories" - has been fully addressed by the additional structural/selectivity evidence and by clarifying the role and value of the biosensor. Accordingly, we believe the revised version now presents a coherent and well-supported mechanistic narrative, with strengthened support for the proposed binding mode and a clear demonstration that the novel biosensor provides meaningful added value.

I still think role of the bespoke computational pipeline/MD simulations is unclear, and it's general usefulness is overstated in the current manuscript. I'd like to see the language around this toned down a bit.

We would like again to clarify that two distinct computational components were used for different purposes:

- Without the AI-enabled screening pipeline, we would not have identified the chemical starting point that led to compound 78. The AI-enabled virtual screening pipeline was used to prioritize candidates from a large chemical space (on the order of 10 million compounds) for experimental testing against GPR183. This role is explicitly described in the manuscript. Crucially, this approach enabled identification of the initial hit (compound 43), which subsequently guided our experimental follow-up and ultimately led to the characterization of compound 78 as a tool compound.

- Without the MD analysis, we would not have identified $Y260^{6.51}$ as the key mechanistic residue. Molecular dynamics (MD) simulations were used to interrogate and refine binding hypotheses for the experimentally confirmed tool compound. The MD simulations were used to support the docking-derived pose(s) and to generate testable mechanistic hypotheses about ligand–receptor interactions, including identifying residues predicted to interact with compound 78. These predictions directly motivated targeted mutagenesis/functional experiments that underpin the mechanistic conclusions.

Accordingly, the AI screening and MD address different questions - hit identification/prioritization versus mechanistic prediction of binding and residue involvement.

Still, we have decided to change the title of the study to *Structure-based screening and a conformational biosensor reveal a GPR183 inverse agonist and a switch mechanism for receptor activation*.

Additionally, we have revisited all the instances where AI-based ligand screening or MD simulations were mentioned in Version 2:

AI screening and a conformational biosensor reveal a GPR183 inverse agonist and a switch mechanism for receptor activation

various diseases. Here, using an artificial intelligence-driven virtual screening approach combined with biophysical assays, including a BRET-based conformational biosensor, we report the discovery of novel GPR183 inverse agonists. From an initial *in vitro* screen of 70 compounds followed by hit expansion, a selected candidate - compound **78** - demonstrated potent inhibition of both constitutive and agonist-stimulated Gi activation as well as β-arrestin2 recruitment. Compound **78** effectively blocked agonist-induced migration of peripheral blood mononuclear cells *ex vivo*, matching the potency of the commercially available inverse agonist NIBR189. Binding of compound **78** to the receptor core was confirmed using an intramolecular BRET-based conformational sensor, supported by molecular dynamics simulations and site-directed mutagenesis. Additionally, these data enabled a more detailed analysis of the GPR183 activation

To address the critical need for novel chemical scaffolds, we moved beyond conventional discovery pipelines and instead exploited the predictive power of advanced computational chemistry. We hypothesized that an Artificial Intelligence (AI)-powered Structure-Based Virtual Screening (SBVS) could rapidly and effectively identify unique GPR183 modulators that traditional screening campaigns might overlook. In particular, in this study, we implemented the PyRMD2Dock approach developed by some of us, which leverages the classification power of the Random Matrix Discriminant (RMD) algorithm to enhance the throughput of virtual screening campaigns for drug discovery [32]. This approach was coupled with the use of established

To discover novel GPR183 binders, the PyRMD2Dock protocol was employed as depicted in **Fig. 1a**. It combines the Ligand-Based VS (LBVS) tool PyRMD [56] with one of the most widely used docking software, AutoDock-GPU (AD4-GPU) [35]. By implementing PyRMD2Dock, it is possible to rapidly screen large chemical databases and identify those with the highest predicted binding affinity to a target protein. The first step of this workflow was to randomly select 1 million compounds from the screened database (in our case we selected a set of ~10 million compounds from a subset of lead-like purchasable ZINC20 database [57]), and subject them to docking calculations employing the solved Cryo-EM structure of GPR183 in complex with its endogenous ligand 7α,25-dihydroxycholesterol (PDB 7TUZ [2]). For all the compounds, the predicted lowest binding free energy (ΔG_{AD4}) was extracted, and the results were analysed based on their ΔG_{AD4}

values reported above. PyRMD returned a list of compounds classified as possible GPR183 binders with an associated confidence score (RMD score) of its predictions. Subsequently, the best 50 000 GPR183 binders predicted by PyRMD (i.e., the ones with the highest RMD score) were then docked into the GPR183 receptor, and to further refine the selection, we subsequently applied a pose filtering scheme aimed at selecting the ligands that were predicted to directly contact Y116$^{3.37}$, Y260$^{6.51}$, R87$^{2.60}$, and Y112$^{3.33}$ (numbers in the superscript indicate residue positions according to the Ballesteros-Weinstein numbering scheme [2, 58]). These selection criteria, and a subsequent visual inspection, led to the selection of 70 GPR183 candidate binders

*Molecular dynamics simulation and in vitro validation of the compound **78** binding site*

To provide an atomistic view of its inverse agonism, molecular dynamics (MD) simulations were performed on GPR183 in complex with **78**. A central part of this investigation was to understand how **78** is recognized by the active (PDB ID: 7TUZ) and inactive (PDB ID: 7TUY) [2] states of GPR183. These simulations were attained employing the Amber24 software (Amber manual,

equally stable plateau for the rest of the trajectory. The binding poses of **78** in the active and inactive states of the receptor closely resemble those of the experimentally determined 7α,25-dihydroxycholesterol (agonist) and GSK682753A (inverse agonist) (**Fig. 4a, b**), respectively.

In the active conformation, **78** binds deep within the central cavity of the GPR183 transmembrane domain (**Fig. 4a**). It adopts an elongated, parallel orientation (relative to the transmembrane bundle) to establish contacts with TM3 throughout TM7 **(Fig. 4c)**. This pose is stabilized by key aromatic and polar interactions. Specifically, the *p*-bromophenyl moiety forms an extended π-π interaction with the Y116$^{3.37}$ side chain (TM3), which is strengthened by the electron-withdrawing bromine. Simultaneously, the pyrazole moiety forms a hydrogen bond with the Y260$^{6.51}$ side chain, while the compound's carbonyl oxygen forms hydrogen bonds with both C201$^{5.43}$ and H261$^{6.52}$ **(Supplementary Figure 6d-e)**.

To investigate how compound **78** translocates from a parallel to an orthogonal pose thereby inducing receptor inactivation, Targeted Molecular Dynamics (TMD) simulations were employed. As described above, in the starting active conformation, the pyrazole moiety of **78** (**Fig. 5a**) forms a tight hydrogen bond with Y260$^{6.51}$ (TM6). This Y260$^{6.51}$ is, in turn, in contact with Y112$^{3.33}$ (TM3). This Y112$^{3.33}$-Y260$^{6.51}$ inter-helical contact appears to act as a "lock," stabilizing the receptor in its active form by preventing TM3 from relocating (**Supplementary Fig. 8a-b**). As the TMD simulation progressed, **78** moved from the deep cavity towards the outer, inactive-state-binding pose. Crucially, as **78** moved, it did not break its interaction with Y260$^{6.51}$. Instead, it performed a "hand-off", with this residue's polar contacts switching from the ligand's pyrazole to its pyridazinone nitrogen and carbonyl oxygen. This relocation, with **78** effectively dragging Y260$^{6.51}$ along for the movement, led to the loss of the Y112$^{3.33}$-Y260$^{6.51}$ inter-helical constraint. This frees the Y112$^{3.33}$ side chain, allowing TM3 to undergo the pivotal rearrangement necessary for the receptor to adopt its inactive conformation.

According to this model, if Y260$^{6.51}$ remains locked to Y112$^{3.33}$, the TM3 rearrangement is prevented, and the receptor is stabilized in its active state, which should account for a compound's agonist behaviour. The simulations revealed a key role for Y260$^{6.51}$ in enabling the **78**-induced conformational switch toward the inactive receptor state. Interestingly, a similar pattern of consecutive hydrogen bond acceptor atoms is also present in the reported NIBR189 and GSK682753A inverse agonists, and it is tempting to postulate that the same "hand-off" mechanism

is responsible for the receptor inactivation by this ligand. In contrast, the endogenous agonist 7α,25-dihydroxycholesterol, despite having a 25-hydroxyl group, lacks the necessary hydrogen bond acceptors in its predominantly apolar scaffold, thereby being unable to perform this "hand-off" and translocate.

To experimentally validate the binding mode predicted by our MD studies, we sought to confirm the importance of the key hydrogen bonding residues. As the experiments would be performed on

Here, the discovery pipeline leveraged PyRMD2Dock, a hybrid AI-docking platform designed to enrich VS hits with favourable predicted binding profiles and contact maps aligned with key GPR183 residues. This approach proved effective in identifying promising binders from a large chemical space, yielding a ≥20% initial hit rate from the primary 70-compound screen, and subsequently leading to the selection of compound **43** and its more efficacious analogue, compound **78**. To this end, we successfully establish that the PyRMD2Dock workflow is suitable for the identification of GPCR-targeting small molecules.

Next, MD simulations, substantiated by mutagenesis data, further validated the predicted binding mode, highlighting key interactions with residues e.g. $C201^{5.43}$, $Y260^{6.51}$ and $H261^{6.52}$, which appear essential for ligand engagement and receptor stabilisation in an inactive conformation. Moreover, MD simulations allowed to describe the inactivation mechanism induced by **78** when binding to the GPR183 active state outlining the role of specific residues lining the binding pocket in a hand-off routine taking place between the ligand and the receptor counterpart. In addition, this "hand-off" model suggests that for GPR183, a constitutively active GPCR, the development of effective inverse agonists may require a new approach. Rather than focusing solely on affinity for the inactive state, a more promising strategy might involve designing molecules that proficiently

39

bind to the active state and are capable of inducing and triggering this proposed conformational switch.

Specifically, this study reports the discovery of a GPR183 inverse agonist, identified through an integrated AI-driven SBVS strategy and validated using several biophysical tools, including an innovative conformational BRET-based biosensor. GPR183, a Gi-coupled chemotactic receptor

In summary, this work combines computational innovation, chemical biology, and receptor pharmacology to deliver a novel, potent GPR183 modulator and a new biosensor system, as well as novel insights about the GPR183 receptor activation mechanism. Together, they provide the

To summarise, we have identified two words that might be interpreted as potentially overstating the role and usefulness of AI and MD approaches, and we have revised them accordingly (in Version 3) :

In the Discussion part, we have removed "validated" and changed it to "supported"; we have also removed "allowed to describe" and changed to "allowed to propose":

"Next, MD simulations, substantiated by mutagenesis data, further supported the predicted binding mode, highlighting key interactions with residues e.g. C201$^{5.43}$, Y260$^{6.51}$ and H261$^{6.52}$, which appear essential for ligand engagement and receptor stabilisation in an inactive conformation. Moreover, MD simulations allowed to propose the inactivation mechanism induced by 78 when binding to the GPR183 active state, outlining the role of specific residues lining the binding pocket in a hand-off routine taking place between the ligand and the receptor counterpart" – p.42.

References:

1. Kenakin, T., *A Pharmacology Primer: Techniques for More Effective and Strategic Drug Discovery*. 6 ed. 2022: Elsevier. 502.
2. Gratz, L., et al., *NanoBiT- and NanoBiT/BRET-based assays allow the analysis of binding kinetics of Wnt-3a to endogenous Frizzled 7 in a colorectal cancer model.* Br J Pharmacol, 2024. **181**(20): p. 3819-3835.
3. Wright, S.C., et al., *FZD5 is a Galphaq-coupled receptor that exhibits the functional hallmarks of prototypical GPCRs.* Sci Signal, 2018. **11**(559).
4. Zeng, R., et al., *Discovery of a Highly Potent Oxysterol Receptor GPR183 Antagonist Bearing the Benzo[d]thiazole Structural Motif for the Treatment of Inflammatory Bowel Disease (IBD).* J Med Chem, 2024. **67**(5): p. 3520-3541.
5. Liu, C., et al., *Oxysterols direct B-cell migration through EBI2.* Nature, 2011. **475**(7357): p. 519-23.
6. Morales, P., et al., *Progress on the development of Class A GPCR-biased ligands.* Br J Pharmacol, 2025. **182**(14): p. 3249-3300.
7. Vandeputte, M.M., L. Vasudevan, and C.P. Stove, *In vitro functional assays as a tool to study new synthetic opioids at the mu-opioid receptor: Potential, pitfalls and progress.* Pharmacol Ther, 2022. **235**: p. 108161.
8. Kenakin, T.P., *Cellular assays as portals to seven-transmembrane receptor-based drug discovery.* Nat Rev Drug Discov, 2009. **8**(8): p. 617-26.
9. Wright, S.C. and M. Bouvier, *Illuminating the complexity of GPCR pathway selectivity - advances in biosensor development.* Curr Opin Struct Biol, 2021. **69**: p. 142-149.
10. Sajkowska, J.J., C.H. Tsang, and P. Kozielewicz, *Application of FRET- and BRET-based live-cell biosensors in deorphanization and ligand discovery studies on orphan G protein-coupled receptors.* SLAS Discov, 2024. **29**(6): p. 100174.
11. Xi, J., et al., *Discovery of a First-in-Class GPR183 Antagonist for the Potential Treatment of Rheumatoid Arthritis.* J Med Chem, 2023. **66**(23): p. 15926-15943.
12. Preiss, L.C., et al., *Multi-Well Array Culture of Primary Human Hepatocyte Spheroids for Clearance Extrapolation of Slowly Metabolized Compounds.* AAPS J, 2022. **24**(2): p. 41.
13. Preiss, L.C., et al., *Comparison of Human Long-Term Liver Models for Clearance Prediction of Slowly Metabolized Compounds.* Drug Metab Dispos, 2024. **52**(6): p. 539-547.
14. Hannedouche, S., et al., *Oxysterols direct immune cell migration via EBI2.* Nature, 2011. **475**(7357): p. 524-7.
15. Christopoulos, A. and T. Kenakin, *G protein-coupled receptor allosterism and complexing.* Pharmacol Rev, 2002. **54**(2): p. 323-74.

Rebuttal Letter 3

We thank Reviewer 4 for their positive assessment of our study and for their constructive comments. We are pleased that the reviewer found our responses to Reviewer 2 to be scientifically justified and our explanations appropriate.

Finally, we apologize for the confusion regarding Fig. 3. The labelling has now been corrected in the revised manuscript.
